# Comparison of Integrated Outpatient Palliative Care With Standard Care in Patients With Parkinson Disease and Related Disorders: A Randomized Clinical Trial

**DOI:** 10.1001/jamaneurol.2019.4992

**Published:** 2020-02-10

**Authors:** Benzi M. Kluger, Janis Miyasaki, Maya Katz, Nicholas Galifianakis, Kirk Hall, Steven Pantilat, Ryan Khan, Cari Friedman, Wendy Cernik, Yuika Goto, Judith Long, Diane Fairclough, Stefan Sillau, Jean S. Kutner

**Affiliations:** 1 Department of Neurology, Anschutz Medical Campus, University of Colorado, Denver, Aurora; 2 Now with Department of Neurology, University of Rochester Medical Center, Rochester, New York; 3 Department of Neurology, University of Alberta, Edmonton, Alberta, Canada; 4 Department of Neurology, University of California, San Francisco, San Francisco; 5 Division of Palliative Medicine, Department of Medicine, University of California, San Francisco, San Francisco; 6 Department of Biostatistics and Informatics, School of Public Health, University of Colorado, Aurora; 7 Division of General Internal Medicine, Department of Medicine, School of Medicine, University of Colorado, Aurora

## Abstract

**Importance:**

Parkinson disease and related disorders (PDRD) have consequences for quality of life (QoL) and are the 14th leading cause of death in the United States. Despite growing interest in palliative care (PC) for persons with PDRD, few studies are available supporting its effectiveness.

**Objective:**

To determine if outpatient PC is associated with improvements in patient-centered outcomes compared with standard care among patients with PDRD and their caregivers.

**Design, Setting, and Participants:**

This randomized clinical trial enrolled participants at 3 academic tertiary care centers between November 1, 2015, and September 30, 2017, and followed them up for 1 year. A total of 584 persons with PDRD were referred to the study. Of those, 351 persons were excluded by phone and 23 were excluded during in-person screenings. Patients were eligible to participate if they had PDRD and moderate to high PC needs. Patients were excluded if they had urgent PC needs, another diagnosis meriting PC, were already receiving PC, or were unable or unwilling to follow the study protocol. Enrolled participants were assigned to receive standard care plus outpatient integrated PC or standard care alone. Data were analyzed between November 1, 2018, and December 9, 2019.

**Interventions:**

Outpatient integrated PC administered by a neurologist, social worker, chaplain, and nurse using PC checklists, with guidance and selective involvement from a palliative medicine specialist. Standard care was provided by a neurologist and a primary care practitioner.

**Main Outcomes and Measures:**

The primary outcomes were the differences in patient quality of life (QoL; measured by the Quality of Life in Alzheimer Disease scale) and caregiver burden (measured by the Zarit Burden Interview) between the PC intervention and standard care groups at 6 months.

**Results:**

A total of 210 patients with PDRD (135 men [64.3%]; mean [SD] age, 70.1 [8.2] years) and 175 caregivers (128 women [73.1%]; mean [SD] age, 66.1 [11.1] years) were enrolled in the study; 193 participants (91.9%) were white and non-Hispanic. Compared with participants receiving standard care alone at 6 months, participants receiving the PC intervention had better QoL (mean [SD], 0.66 [5.5] improvement vs 0.84 [4.2] worsening; treatment effect estimate, 1.87; 95% CI, 0.47-3.27; *P* = .009). No significant difference was observed in caregiver burden (mean [SD], 2.3 [5.0] improvement vs 1.2 [5.6] improvement in the standard care group; treatment effect estimate, −1.62; 95% CI, −3.32 to 0.09; *P* = .06). Other significant differences favoring the PC intervention included nonmotor symptom burden, motor symptom severity, completion of advance directives, caregiver anxiety, and caregiver burden at 12 months. No outcomes favored standard care alone. Secondary analyses suggested that benefits were greater for persons with higher PC needs.

**Conclusions and Relevance:**

Outpatient PC is associated with benefits among patients with PDRD compared with standard care alone. This study supports efforts to integrate PC into PDRD care. The lack of diversity and implementation of PC at experienced centers suggests a need for implementation research in other populations and care settings.

**Trial Registration:**

ClinicalTrials.gov Identifier: NCT02533921

## Key Points

**Question:**

Is outpatient palliative care associated with improvements in patient or caregiver outcomes compared with current standards of care among persons with Parkinson disease and related disorders?

**Findings:**

In this randomized clinical trial of 210 patients with Parkinson disease and related disorders and 175 caregivers, patients receiving palliative care had better quality of life at 6 months (primary outcome) as well as better symptom burden and rates of advance directive completion. No significant difference was found in caregiver burden at 6 months (coprimary outcome).

**Meaning:**

Outpatient palliative care may improve certain patient and caregiver outcomes associated with Parkinson disease and related disorders.

## Teaser

This randomized clinical trial examines whether outpatient integrated palliative care is associated with patient-centered outcomes and caregiver burden compared with standard care alone among patients with Parkinson disease and related disorders and their caregivers.

## Introduction

The field of palliative care (PC) aims to improve quality of life (QoL) and reduce suffering in persons with serious illness by addressing medical symptoms, psychosocial issues, and advance care planning.^1^ Although PC is frequently equated with hospice care and cancer,^2^ recognition of the potential relevance of PC in other contexts has expanded substantially over the past decade to include earlier deployment,^3^ delivery to noncancer populations,^4^ delivery in outpatient settings,^5^ and delivery by persons not specializing in palliative medicine (primary PC)^6^ or by disease-specific clinics including palliative medicine input (integrated PC).^7^ Despite growing interest in this more comprehensive concept of PC and high projected needs (associated with the growing global burden of neurodegenerative illness^8^ and shortfalls in the palliative medicine workforce^9^), few studies have tested the effectiveness of these approaches.

Parkinson disease (PD) affects 1% to 2% of people older than 65 years and is the 14th leading cause of death in the United States.^8,10^ While traditionally described by its motor symptoms, PD also includes nonmotor symptoms, such as pain and dementia, which are common and associated with mortality, QoL, nursing home placement, and caregiver distress.^11,12^ Other forms of parkinsonism, collectively referred to as PD and related disorders (PDRD), share core features of PD but have additional symptoms and worse prognoses. A growing number of centers now apply PC to patients with PDRD, typically using outpatient integrated PC led by a neurologist with fellowship or informal PC training.^13^ Previous studies have reported that this model of integrated PC is feasible, acceptable, and potentially efficacious in PDRD.^14,15^ Our primary goal in this study was to compare outpatient integrated PC with standard care alone to evaluate its effectiveness on patient QoL, caregiver burden, and other patient-centered outcomes at 6 months (primary time point), with data collected for up to 12 months to understand long-term outcomes.

## Methods

### Study Design

From November 1, 2015, to September 30, 2017, we enrolled patients with PDRD (and their caregivers when available) who had moderate to high PC needs in a nonblinded randomized pragmatic comparative effectiveness clinical trial of outpatient integrated PC vs standard care alone. Pragmatic clinical trial elements included broad inclusion criteria, nonscripted standard care, use of different models of integrated PC, and self-reported outcomes.^16^ The study was conducted at 3 academic tertiary medical centers: the University of Alberta (Edmonton, Alberta, Canada), the University of Colorado (Aurora), and the University of California, San Francisco. Before patient enrollment, the study protocol was approved by the institutional review boards of the 3 medical centers and posted on ClinicalTrials.gov. All participants provided informed consent or, if they lacked the capacity to consent,^17^ provided assent, with informed consent obtained from their designated medical proxy. The complete protocol and statistical analysis plan are available in Supplement 1. This study followed the Consolidated Standards of Reporting Trials (CONSORT) reporting guideline for randomized clinical trials.

Participants were randomized using a 1:1 ratio and stratified by site, presence of a caregiver, and presence of dementia. A randomization list for the sequence of enrolled patients was prepared for each combination of strata, with each sequence divided into blocks of 4, within which 2 patients were randomly chosen for each treatment group. Randomization assignment was revealed to the coordinator after the baseline visit. Participants assigned to the PC intervention group received outpatient PC visits every 3 months for 1 year. Physician and other health care visits for standard care were recorded but not mandated. Patient and caregiver outcomes were recorded at baseline and every 3 months for 12 months. The coprimary outcomes were group differences in patient QoL and caregiver burden at 6 months.

In alignment with the principles of the Patient-Centered Outcomes Research Institute, we engaged a patient and caregiver council (Palliative Care and Parkinson’s Disease Patient Advisory Council),^18^ which was led by an author (K. H.) with established interests in PC.^19^ The council enhanced our study and assisted with study protocols, recruitment, interpretation of results, and preparation of manuscripts.^20^

### Participants

Participants were referred from academic medical centers, community neurologists, regional PD support organizations, and clinical trial websites (ClinicalTrials.gov and foxtrialfinder.org). A total of 584 persons with PDRD were referred to the study. Of those, 351 persons were excluded by phone and 23 were excluded during in-person screenings. Patients were eligible to participate if they were fluent in English, had probable PD,^21^ had another PDRD diagnosis (multiple system atrophy, corticobasal degeneration, progressive supranuclear palsy, or Lewy body dementia), and had moderate to high PC needs based on the Palliative Care Needs Assessment Tool (PC-NAT) modified for PD (eMethods 1 in Supplement 2).^22^ Participants were excluded if they had urgent PC needs based on the clinical judgment of the site investigator, were unable to commit to study procedures, had other illnesses that could require PC, or were already receiving PC.

Caregivers were identified with the answer to the question, could you please tell us the 1 person who helps you the most with your PD outside of the clinic? For patients with dementia, family caregivers could be self-identified to obtain relevant data. Participants self-reported their race and ethnicity to assess diversity in the sample and its association with outcomes.

### Standard Care and PC Intervention

Standard care was provided by the patient’s primary care physician and a neurologist. We considered the involvement of a primary care physician and neurologist to be standard care based on evidence indicating that neurologist involvement is associated with improvements in outcomes and that most patients with PD in the United States receive care from a neurologist.^23^ Standard care for patients with PD, even in academic settings, is rarely team-based; however, practitioners can refer patients to other services at their discretion. Patients who were not established with a neurologist at enrollment were scheduled for an appointment with a neurologist to establish care.

Our intervention consisted of standard care plus outpatient PC. Participants could elect to transfer their neurology care to the PC team to consolidate care. Palliative care visits were performed in person or by telemedicine every 3 months. Visits were supplemented with phone calls at the discretion of the PC team, and participants could contact the PC team as needed. After-visit summaries were provided to the patient, and standard clinic notes were provided to the primary care physician and neurologist. Suggestions for care outside of PC issues were provided to the patient’s standard care team.

The interdisciplinary team consisted of a palliative neurologist with informal training in PC (eg, education through a palliative and end-of-life care workshop); a nurse, social worker, and chaplain with PD experience; and a board-certified palliative medicine physician (eMethods 2 in Supplement 2). Although all academic teams worked within integrated PC models, they varied in clinic flow and their use of the palliative medicine specialist. The University of Alberta team met with patients as a whole team, including the palliative medicine specialist; the University of Colorado team met with patients sequentially, with the palliative medicine specialist primarily involved in informal consultations; and the University of California, San Francisco, team used a mixture of these approaches.^24^ Palliative medicine specialists primarily focused on the complex goals of care discussions and symptom management. The typical visit duration was 2 to 2.5 hours and addressed nonmotor symptoms, goals of care, anticipatory guidance, difficult emotions, and caregiver support. To improve fidelity and enhance the dissemination of information, visits were standardized using checklists for each team member (eMethods 3 in Supplement 2).

### Outcome Measures

Our coprimary outcomes were the group differences in the change in patient QoL, which was QOL measured using the Quality of Life in Alzheimer’s Disease (QoL-AD) scale,^25^ and caregiver burden, which was measured using the 12-item Zarit Burden Interview (ZBI-12), at 6 months.^26^ The QoL-AD is a 13-item scale in which patients (and caregivers, if present) rate items from poor to excellent (score range, 13-52, with 13 indicating poor QoL and 52 indicating excellent QoL). The QoL-AD was chosen for its brevity, validation for use among patients with PD-related dementia, validated proxy reporting, sensitivity to change, and coverage of issues relevant to patients with PD in qualitative interviews.^15,27,28,29,30^ We used the ZBI-12 (score range, 0-48, with 0-10 indicating no to mild caregiver burden, 11-20 indicating mild to moderate caregiver burden, and 20-48 indicating high caregiver burden) because it is the most commonly used measure of distress among caregivers of patients with PD,^31,32^ and it has good clinimetric properties and responsiveness.^33,34,35,36^

Symptom burden was assessed using the Edmonton Symptom Assessment Scale–Revised for Parkinson’s Disease, which is a 14-item scale that measures the severity of symptoms on a scale of 1 to 10 (score range, 0-140, with 0 indicating no symptom burden and 140 indicating high symptom burden).^13^ Health-related QoL was assessed using the 39-item Parkinson’s Disease Questionnaire (score range, 0-100, with lower scores indicating better QoL and higher scores indicating worse QoL).^37^ Patient and caregiver mood was assessed using the Hospital Anxiety and Depression Scale, a 14-item scale with validated subscales for depression and anxiety (score range, 0-21 for each subscale, with 0 indicating little to no likelihood of depression or anxiety and 21 indicating high likelihood of depression or anxiety).^38^ Patient and caregiver grief was assessed using the 12-item Prolonged Grief Disorder questionnaire (score range, 0-44, with 0 indicating minimum symptoms of prolonged grief disorder and 44 indicating maximum symptoms of prolonged grief disorder).^39^ Patient and caregiver spiritual well-being was assessed using the Functional Assessment of Chronic Illness Therapy–Spiritual Well-Being, a 12-item scale (score range, 0-48, with 0 indicating low spiritual well-being and 48 indicating high spiritual well-being).^40^ Patients and caregivers provided their clinical global impression of change on a 7-point scale, with −3 indicating worse, 0 indicating no change, and 3 indicating improved.

Patient and caregiver patterns of health care use were assessed every 6 weeks using surveys drawn or modified from the Ambulatory and Home Care Record (eMethods 4 in Supplement 2).^41^ A trained, unblinded rater (B. M. K., J. M., N. G., or M. K.) assessed motor symptoms using the motor subscale of the Unified Parkinson’s Disease Rating Scale (score range, 0-56, with 0 indicating no motor symptoms and 56 indicating maximum motor symptoms)^42^ and cognitive function using the Montreal Cognitive Assessment (score range, 0-30, with 0 indicating maximum cognitive impairment and 30 indicating no cognitive impairment)^43^ at baseline, 6 months, and 12 months. Completion of advance directives was assessed at baseline, 6 months, and 12 months via self-report.

### Statistical Analysis

The study comprised 210 patients and allowed for the withdrawal of 30 patients, which provided an estimated power to detect a statistically significant between-group difference of 91% for the QoL-AD (90 participants per arm) and 84% for the ZBI-12 (72 participants per arm), with an α of .05. The study had sufficient power to detect a moderate effect size of 0.5 times the within-group SD.

Descriptive statistics were used to estimate frequencies, means, and SDs. Group differences at baseline were assessed using a *t* test for continuous and scale variables and an χ^2^ or Fisher exact test for categorical variables. Longitudinal differences between groups were analyzed using mixed-model regression. The primary analysis was based on intention-to-treat and adjusted models to account for potentially important clinical variables (sex, age, disease duration, baseline Montreal Cognitive Assessment score, Hoehn and Yahr stage, study site, and presence of a caregiver) and for variables associated with missing data that could disturb missing-at-random assumptions, including race (white vs nonwhite), marital status, and educational level (less than a college degree vs college degree or higher). Sensitivity analyses were performed using unadjusted models, models imputing missing data, and as-treated models, with analysis according to the duration of actual treatment received. Potential treatment modifiers were assessed as interaction terms, with interaction models tested against the original model. We applied a Benjamini-Hochberg procedure to all outcomes at 6 and 12 months to control the false discovery rate at α .05. Statistical analyses were performed using SAS software, version 9.4 (SAS Institute). Data were analyzed between November 1, 2018, and December 9, 2019.

## Results

**Figure 1.**
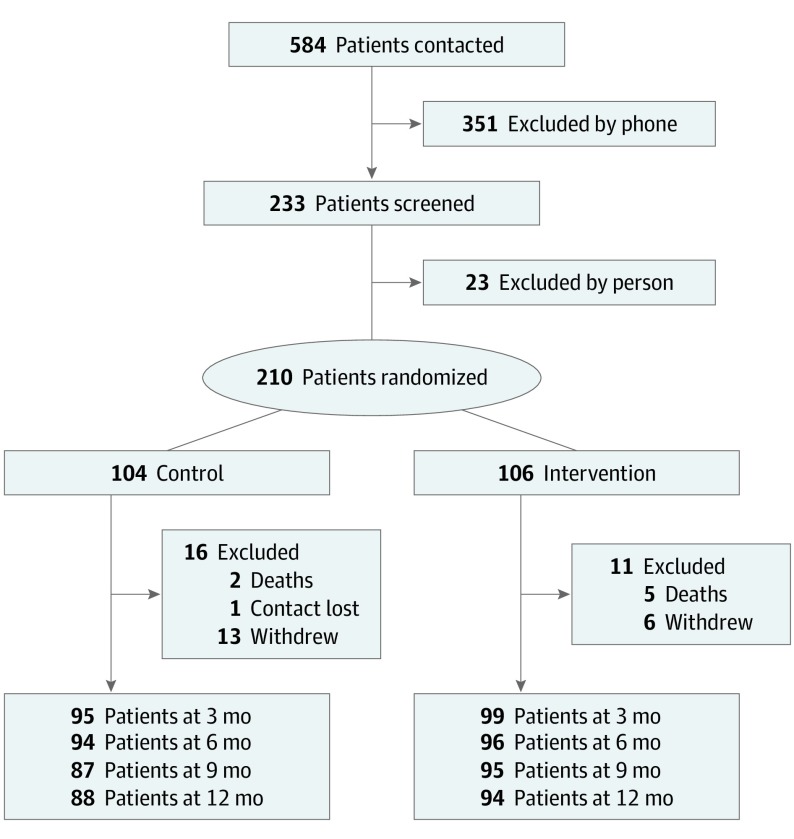
CONSORT Patient Flow Diagram

A total of 210 patients with PDRD (135 men [64.3%]; mean [SD] age, 70.1 [8.2] years) and 175 caregivers (128 women [73.1%]; mean [SD] age, 66.1 [11.1] years) were enrolled in the study. (Figure 1). Of those, 193 (91.9%) were white and non-Hispanic. A total of 106 patients (65 men [61.3%]; mean [SD] age, 69.5 [8.3] years) and 87 caregivers (62 women [71.3%]; mean [SD age, 69.7 [11.7] years) were randomized to the PC intervention group, and 104 patients (70 mean [67.3%]; mean [SD] age, 70.7 [8.0] years) and 88 caregivers (66 women [75.0%]; mean [SD] age, 66.4 [11.1] years) were randomized to the standard care group (Table 1). One hundred participants (94.3%) in the PC intervention group and 93 participants in the standard care group (89.4%) were white and non-Hispanic. Adherence in the PC intervention group was high, with 87 of 106 randomized patients (82.1%) completing all planned outpatient visits. In the standard care group, the estimated rate of neurologist visits per person per year was 3.16, and the estimated rate of primary care physician visits per person year was 4.66. Twelve patients crossed over from the standard care to the PC intervention group, and 2 patients (1 from each group) were referred to hospice care. Telemedicine was used for at least 1 visit by 19 of 106 patients (17.9%) in the PC intervention group, and the palliative medicine physician was directly involved in the care of 48 of 104 patients (46.2%).

**Table 1.** Baseline Characteristics of Participants

Variable	Care Group, No. (%)		*P* Value
	Standard	Palliative	
Patient, No.	104	106	NA
Caregiver, No.	88	87	NA
Patient characteristic			
Age, mean (SD), y	70.7 (8.0)	69.5 (8.3)	.29
Male sex	70 (67.3)	65 (61.3)	.37
Race (by checklist)			
White	93 (89.4)	100 (94.3)	.19
Asian	4 (3.9)	2 (1.9)	.44
Black	2 (1.9)	1 (0.9)	.62
Other, mixed, or no response	4 (4.9)	3 (2.8)	.70
No response	1 (1.0)	0	.49
Hispanic ethnicity	3 (2.9)	3 (2.8)	>.99
Marital status			
Currently married	82 (78.9)	79 (74.5)	.45 (if binary)
Never married	5 (4.8)	5 (4.7)	.93
Separated	1 (1.0)	3 (2.8)	
Widowed	7 (6.7)	7 (6.6)	
Divorced	8 (7.7)	11 (10.4)	
Unknown	1 (1.0)	1 (0.9)	
Educational level			
Grades 1-11	7 (6.9)	6 (5.7)	.006
High school diploma	0 (0.0)	12 (11.3)	
Some college	18 (17.7)	12 (11.3)	
Associate degree	6 (5.9)	9 (8.5)	
Bachelor degree	27 (26.5)	22 (20.8)	
Higher than bachelor degree	44 (43.1)	45 (42.5)	
Annual income, $			
Total No.	90	90	.56
≤29 999	13 (14.4)	12 (13.3)	
30 000-39 999	4 (4.4)	1 (1.1)	
40 000-49 999	8 (8.9)	10 (11.1)	
50 000-59 999	4 (4.4)	10 (11.1)	
60 000-74 999	12 (13.3)	14 (15.6)	
75 000-99 999	23 (23.6)	20 (22.2)	
>100 000	25 (27.8)	23 (25.6)	
Unknown	1 (1.1)	0	
Disease duration, mean (SD), mo	114.3 (79.2)	116.5 (83.7)	.85
Dementia present (by clinical criteria)	30 (28.9)	32 (30.5)	.80
Currently seeing neurologist	103 (99.0)	103 (97.2)	.62
Atypical parkinsonian conditions	12 (11.5)	13 (12.3)	.87
Completed health care proxy	77 (75.5)	78 (75.0)	.94
Completed advance directive	68 (66.7)	61 (58.7)	.23
Caregiver present	88 (84.6)	87 (82.1)	.62
Caregiver shares household with patient	82 (93.2)	77 (88.5)	.28
Caregiver characteristic			
Female sex	66 (75.0)	62 (71.3)	.58
Age, mean (SD), y	66.4 (11.1)	65.7 (11.7)	.69
Caregiving duration, mean (SD), mo	66.3 (50.5)	70.7 (73.2)	.65
Relationship to patient			
Spouse	73 (83.0)	70 (80.5)	.72
Adult child	7 (8.0)	10 (11.5)	
Other	8 (9.1)	7 (8.0)	
Race (by checklist)			
White	77 (87.5)	82 (94.3)	.12
Asian	5 (5.7)	3 (3.5)	.72
Black	1 (1.1)	0	>.99
Other, mixed, or no response	4 (4.5)	2 (2.4)	.68
Pacific Islander	0	0	NA
No response	1 (1.1)	0	>.99
Hispanic ethnicity	3 (3.4)	5 (5.8)	.49
Study site			
University of Colorado	37 (35.6)	36 (34.0)	.97
University of California, San Francisco	34 (32.7)	36 (34.0)	
University of Alberta	33 (31.7)	34 (32.1)	
Assessment score			
MoCA, mean (SD)	23.7 (5.1)	24.0 (4.8)	.67
UPDRS motor subscale, mean (SD)	37.7 (17.6)	42.8 (19.4)	.05
QoL-AD, mean (SD)	34.3 (5.6)	33.9 (5.7)	.61
ZBI-12, mean (SD)	16.8 (7.7)	17.9 (8.0)	.37
Hoehn and Yahr stage			
1	0	0	.17
1.5	0	2 (1.9)	
2	34 (34.0)	25 (24.0)	
2.5	30 (30.0)	24 (23.1)	
3	15 (15.0)	25 (24.0)	
4	12 (12.0)	14 (13.5)	
5	9 (9.0)	14 (13.5)	

Abbreviations: MoCA, Montreal Cognitive Assessment; NA, not applicable; QoL-AD, Quality of Life in Alzheimer’s Disease Scale; UPDRS, Unified Parkinson’s Disease Rating Scale; ZBI-12, Zarit Burden Interview 12-item scale.

**Figure 2.**
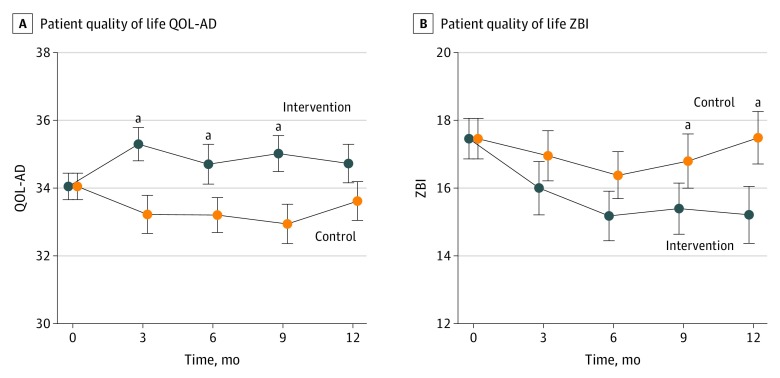
Patient-Reported and Caregiver-Reported Outcomes A, Patient-reported outcomes. QoL-AD indicates Quality of Life in Alzheimer Disease Scale. B, Caregiver-reported outcomes. ZBI-12 indicates Zarit Burden Interview 12-item scale. Error bars indicate the SE. ^a^Points with significant group differences in the primary adjusted model.

Compared with the standard care group, participants in the PC intervention group had better QoL (mean [SD], 0.66 [5.5] improvement vs 0.84 [4.2] worsening; treatment effect estimate, 1.87; 95% CI, 0.47-3.27; *P* = .009; Figure 2A) at 6 months. These effects were similar in the unadjusted model and were increased in models that imputed missing data and accounted for treatment received. The same pattern of statistical significance remained when missing data was filled in with multiple imputation; the 6-month estimated treatment effect was 1.82 (95% CI, 0.16-3.47; *P* = .03), and the 12-month estimated treatment effect was 1.26 (95% CI, −0.20 to 2.72; *P* = .09; eResults and eTables 1, 2, and 3 in Supplement 2). The QoL for patients and caregivers was jointly modeled so they could contribute information to each other. Factoring crossover in treatment made the estimated treatment effects stronger. For crossover models with covariate adjustment (but without missing data imputation of joint modeling), the estimated treatment effects were 2.48 (95% CI, 1.19-3.76; *P* < .001) for 6 months and 1.87 (95% CI, 0.51-3.24; *P* = .007) for 12 months. When the imputed data and joint modeling were added, the treatment effect estimates were 2.00 (95% CI, 0.52-3.49; *P* = .009) for 6 months and 1.48 (95% CI, 0.06-2.91; *P* = .04). Higher PC needs at baseline (assessed by the PC-NAT) were significantly associated with greater benefit from the PC intervention. At 12 months, the treatment effect for women was 2.91 (95% CI, 0.67-5.14; *P* = .01) and for men was 0.47 (95% CI, −1.22 to 2.16; *P* = .58), indicating a 2.43 (95% CI, −0.36 to 5.23; *P* = .09) greater treatment effect for women than men. Other potential treatment effect modifiers (age, mood, caregiver burden, symptom burden, disease severity, and cognition) were not significant. Compared with the standard care group, the PC intervention group had a higher proportion of persons who experienced clinically significant (defined as a change in the QoL-AD of at least 3 points)^44^ benefit (20% in the standard care group vs 35% in the PC intervention group; *P* = .02), and a lower proportion of persons who experienced clinically significant worsening (41% in the standard care group vs 25% in the PC intervention group; *P* = .02).

Although the PC intervention group experienced a statistically significant reduction in caregiver burden as measured by ZBI-12 scores (−2.28 points; 95% CI, −3.38 to −1.18; *P* < .001) compared with the standard care group (−1.08 points; 95% CI, −2.28 to 0.12; *P* = .08) at 6 months, the difference between groups was not statistically significant in our primary analysis (mean [SD], 2.3 [5.0] improvement in the PC intervention group vs 1.2 [5.6] improvement in the standard care group; treatment effect estimate, −1.62; 95% CI, −3.32 to 0.09; *P* = .06). However, the difference between groups was statistically significant at 12 months (treatment effect estimate, −2.60; 95% CI, −4.58 to −0.61; *P* = .01; Figure 2B).

The strengths of these treatment effects were increased in models that imputed missing data and accounted for treatment as received, and they were slightly decreased in unadjusted models. When missing data was filled in with multiple imputation, the treatment effects were strengthened; the 6-month estimated treatment effect was statistically significant at −2.63 (95% CI, −4.46 to −0.80; *P* = .006), and the 12-month estimated treatment effect was −2.89 (95% CI, −4.93 to −0.85; *P* = .006; eResults and eTables 1, 2, and 3 in Supplement 2).). For crossover models with covariate adjustment (but without missing data imputation), the estimated treatment effects were −1.61 (95% CI, −3.23 to 0.01; *P* = .02) for 6 months and −2.72 (95% CI, −4.74 to −0.71; *P* = .008) for 12 months. When the imputed data were added, the treatment effect estimates were −2.64 (95% CI, −4.35 to −0.93; *P* = .003) for 6 months and −3.10 (95% CI, −5.15 to −1.05; *P* = .004). Higher PC-NAT scores, lower Montreal Cognitive Assessment scores, and worse grief were significantly associated with greater caregiver burden benefit (measured by the ZBI-12) at 12 months.

Other effects favoring the PC intervention included symptom burden, health-related QoL, grief, caregiver anxiety, the peace subscale of caregiver spiritual well-being (measured by the Functional Assessment of Chronic Illness Therapy–Spiritual Well-Being), and both patient and caregiver global impressions of change (Table 2). No group differences in patient mood or spiritual well-being and no outcomes favoring standard care alone were observed.

**Table 2.** Differences in Primary and Secondary Outcomes Between Groups

Outcome Measure	Time, mo	Standard Care Group		Palliative Care Intervention Group		Difference Between Groups^a^	
		Estimate (95% CI)	*P* Value	Estimate (95% CI)	*P* Value	Estimate (95% CI)	*P* Value
QOL−AD	6	−0.84 (−1.68 to 0.01)	.05	0.66 (−0.43 to 1.75)	.23	1.87 (0.47 to 3.27)	.009^b^
	12	−0.43 (−1.37 to 0.50)	.36	0.68 (−0.38 to 0.73)	.21	1.36 (−0.01 to 2.73)	.05
QOL−AD caregiver perspective on patient	6	−1.40 (−2.38 to −0.43)	.005	2.09 (0.93 to 3.25)	<.001	2.82 (1.46 to 4.17)	<.001^b^
	12	−0.76 (−1.75 to 0.23)	.13	1.81 (0.72 to 2.90)	.001	1.93 (0.51 to 3.36)	<.001^b^
ZBI	6	−1.08 (−2.28 to 0.12)	.08	−2.28 (−3.38 to −1.18)	<.001	−1.62 (−3.32 to 0.09)	.06
	12	−0.02 (−1.32 to 1.37)	.97	−2.25 (−3.56 to −0.94)	.001	−2.60 (−4.58 to −0.61)	.01^b^
ESAS−PD	6	−0.45 (−3.86 to 2.96)	.80	−6.81 (−10.46 to −3.15)	<.001	−7.15 (−11.89 to −2.41)	.003^b^
	12	−0.73 (−4.97 to 3.51)	.73	−9.66 (−13.52 to −5.80)	<.001	−8.27 (−13.90 to −2.64)	.004^b^
PDQ−39	6	−1.20 (−3.57 to 1.18)	.23	−3.04 (−5.13 to −0.94)	.009	−2.63 (−5.72 to 0.46)	.10
	12	−0.34 (−2.66 to 1.97)	.09	−3.04 (−5.46 to −0.94)	.005	−4.05 (−7.25 to −0.84)	.01^b^
UPDRS motor score	6	2.15 (0.04 to 4.27)	.05	−2.98 (−5.79 to −0.18)	.04	−5.98 (−9.54 to −2.43)	.001^b^
	12	2.45 (−0.36 to 5.26)	.09	−1.38 (−4.78 to 2.02)	.42	−3.91 (−8.38 to 0.56)	.09
MOCA	6	−0.14 (−0.82 to 0.55)	.69	0.17 (−0.55 to 0.90)	.64	0.17 (−0.88 to 1.22)	.75
	12	−1.05 (−1.78 to −0.32)	.005	0.14 (−0.57 to 0.85)	.70	1.36 (0.34 to 2.38)	.01^b^
HADS, depression	6	−0.20 (−0.73 to 0.32)	.44	−0.34 (−0.97 to 0.30)	.29	−0.57 (−1.40 to 0.25)	.17
	12	0.12 (−0.45 to 0.69)	.66	−0.33 (−0.92 to 0.25)	.26	−0.52 (−1.33 to 0.29)	.21
HADS, anxiety	6	−0.73 (−1.35 to −0.11)	.02	−1.19 (−1.71 to −0.68)	<.001	−0.66 (−1.44 to 0.13)	.13
	12	−1.42 (−2.04 to −0.80)	<.001	−1.30 (−1.91 to −0.69)	<.001	0.12 (−0.71 to 0.95)	.78
PG−12	6	−0.68 (−2.05 to 0.68)	.32	−2.63 (−3.91 to −1.35)	<.001	−2.24 (−4.15 to −0.60)	.02
	12	−1.31 (−2.73 to 0.11)	.07	−2.61 (−3.92 to −1.31)	<.001	−1.80 (−3.75 to 0.14)	.07
FACIT−SW	6	1.10 (−0.29 to 2.49)	.12	1.17 (−0.01 to 2.35)	.05	0.71 (−1.12 to 2.55)	.44
	12	2.30 (0.76 to 3.83)	.004	0.61 (−0.83 to 2.04)	.40	−1.65 (−3.69 to 0.40)	.11
FACIT−SW, meaning	6	0.41 (−0.04 to 0.87)	.08	0.23 (−0.26 to 0.71)	.36	0.16 (−0.53 to 0.84)	.65
	12	0.61 (0.08 to 1.14)	.02	0.42 (−0.17 to 1.00)	.16	−0.00 (−0.77 to 0.77)	.99
FACIT−SW, peace	6	0.65 (0.07 to 1.23)	.03	0.57 (0.03 to 1.11)	.04	0.14 (−0.64 to 0.93)	.72
	12	1.09 (0.48 to 1.70)	.001	0.17 (−0.48 to 0.83)	.60	−0.87 (−1.71 to −0.02)	.04
FACIT−SW, faith	6	−0.00 (−0.76 to 0.76)	.99	0.36 (−0.23 to 0.94)	.23	0.50 (−0.48 to 1.48)	.32
	12	0.53 (−0.19 to 1.24)	.15	0.04 (−0.52 to 0.61)	.88	−0.54 (−1.46 to 0.38)	.25
Patient CGIC	6	−0.46 (−0.72 to −0.19)	.001	0.29 (−0.01 to 0.59)	.06	0.85 (0.44 to 1.27)	<.001^b^
	12	−0.59 (−0.87 to −0.30)	<.001	0.41 (0.08 to 0.75)	.02	1.21 (0.78 to 1.64)	<.001^b^
Caregiver HADS, depression	6	−0.20 (−0.68 to 0.29)	.42	−0.36 (−0.99 to 0.28)	.27	−0.49 (−1.32 to 0.34)	.25
	12	0.47 (−0.17 to 1.12)	.15	−0.26 (−0.85 to 0.34)	.40	−0.90 (−1.83 to 0.03)	.06
Caregiver HADS, anxiety	6	−0.52 (−1.21 to 0.16)	.13	−1.21 (−1.90 to −0.52)	.001	−1.06 (−2.11 to −0.02)	.05
	12	−0.40 (−1.13 to 0.34)	.29	−0.68 (−1.37 to 0.02)	.06	−0.43 (−1.46 to 0.61)	.42
Caregiver FACIT−SW	6	−0.27 (−1.42 to 0.89)	.65	0.68 (−0.57 to 1.94)	.28	1.48 (−0.22 to 3.18)	.09
	12	−0.90 (−2.12 to 0.31)	.14	0.42 (−0.81 to 1.66)	.50	1.79 (−0.00 to 3.59)	.05
Caregiver FACIT−SW, meaning	6	−0.05 (−0.47 to 0.38)	.83	0.03 (−0.37 to 0.42)	.90	0.19 (−0.38 to 0.76)	.51
	12	−0.41 (−0.87 to 0.05)	.08	−0.09 (−0.54 to 0.36)	.69	0.41 (−0.25 to 1.07)	.22
Caregiver FACIT−SW, peace	6	0.11 (−0.56 to 0.78)	.75	0.75 (0.15 to 1.34)	.01	1.00 (0.12 to 1.88)	.03
	12	−0.14 (−0.71 to 0.43)	.63	0.67 (0.08 to 1.27)	.03	1.06 (0.21 to 1.90)	.01^b^
Caregiver FACIT−SW, faith	6	−0.24 (−0.78 to 0.31)	.39	−0.09 (−0.74 to 0.56)	.78	0.08 (−0.83 to 0.98)	.86
	12	−0.26 (−0.95 to 0.42)	.44	−0.21 (−0.75 to 0.33)	.43	0.10 (−0.87 to 1.06)	.84
Caregiver CGIC	6	−0.75 (−1.04 to −0.46)	<.001	−0.05 (−0.41 to 0.30)	.76	0.72 (0.27 to 1.17)	.002^b^
	12	−0.81 (−1.11 to −0.50)	<.001	0.36 (−0.07 to 0.79)	.09	1.20 (0.68 to 1.72)	<.001^b^

Abbreviations: CGIC, Clinical Global Assessment of Change; ESAS−PD, Edmonton Symptom Assessment Scale−Parkinson’s Disease; FACIT−SW, Functional Assessment of Chronic Illness Therapy−Spiritual Wellbeing; HADS, Hospital Anxiety and Depression Scale; MOCA, Montreal Cognitive Assessment; PG−12, Prolonged Grief 12−item scale; QOL−AD, Quality of Life Alzheimer’s Disease scale; UPDRS, Unified Parkinson’s Disease Rating Scale Motor Subscore; ZBI, Zarit Burden Inventory.

^a^ Treatment effects and *P* values based on adjusted model.

^b^ Significant under false discovery rate (α = .05) adjustment for 44 treatment effects.

Subgroup analyses for PD vs atypical parkinsonian conditions, dementia vs no dementia, advanced vs mild to moderate disease, and high vs mild to moderate depressive symptoms found no between-group differences (eResults and eTables 1, 2, and 3 in Supplement 2).

A statistically and clinically significant benefit in motor symptoms was observed among participants in the PC intervention group (Table 2). Cognitive function was unchanged at 6 months and statistically, but not clinically, better in the PC group at 12 months. At 6 months, among persons who did not have an advance directive or health care proxy completed at baseline, those randomized to the PC intervention group were significantly more likely to have completed an advance directive (53% [20 of 38] vs 26% [8 of 31] in the standard care group for 6-month visit conditional on not having advanced directive at baseline; *P* = .02) but not a health care proxy (48% [11 of 33] vs 39% [9 of 23] in the standard care group for 6-month visit conditional on not having HCPA at baseline; *P* = .55). Among all participants with completed paperwork, persons in the PC intervention group were more likely to have completed state-specific advance directives (67.0% [59 of 88] vs 30.3% [23 of 76] in the standard care group at 12 months; *P* < .001) and to have filed paperwork with their practitioners (for health care proxy, 67.1% [55 of 82] in the intervention group vs 32.8% [20 of 61] in the standard care group at 12 months; *P* < .001; for advance directive, 83.0% [39 of 47] in the intervention group vs 36.8% [13 of 38] in the standard care group at 12 months; *P* < .001). No significant between-group differences in health care use were found during the study period, although the number of significant events was low (eResults and eTables 3 and 4 in Supplement 2). No adverse events were associated with the PC intervention.

## Discussion

These results show a comparative advantage to outpatient PC compared with standard care in patients with PDRD for several outcomes of interest to patients, families, and other stakeholders. We found that persons randomized to receive integrated PC had better QoL, improved symptom burden, and higher rates and quality of advance directive completion. Our results also suggested a benefit to caregiver burden, although these results were less robust and were only significant in our primary analyses at 12 months. Because the benefits of PC were greatest for those with high PC needs, our results may have underestimated treatment effects because we excluded patients with urgent needs.

Although several studies have reported QoL benefits of standard medical approaches in patients with mild to moderate PD, to our knowledge, little previous research has been conducted regarding interventions to promote QoL in individuals with advanced disease, high nonmotor symptom burden, or dementia. Global symptom burden was improved among participants in the PC intervention group, and we hypothesize that this improvement reflected our systematic approach to the detection of nonmotor symptoms using checklists, as nonmotor symptoms are not frequently mentioned by patients or detected by neurologists.^45^ The benefits observed for caregiver burden were larger at 12 months than at 6 months. Because PDRD are progressive illnesses, it is possible that this delayed benefit reflected the progression of the underlying illness and higher needs at this later time point. The benefits to motor symptoms were clinically significant,^46^ which was unexpected, as our team was not focused on motor symptom management. We hypothesize that motor improvements may have reflected an unanticipated benefit of our PC team’s general goal of encouraging activities that promoted joy, meaning, and connection.

Several novel aspects of this study deserve mention.^20^ First, our inclusion criteria were based on a broad range of potential patient and caregiver PC needs rather than prognoses or definitions of advanced disease. These issues are common reasons for referral to our clinics and reflect a desire to meet patient-centered needs rather than disease-centered markers. Given that persons with higher PC needs based on the PC-NAT experienced greater benefit from the intervention, the modified PC-NAT may be a useful triage tool. Second, our intervention was delivered using an integrated PC model. This model reflects current practice and highlights a need to develop hybrid models of PC that build on the strengths of both disease and PC specialists and that efficiently use our limited pool of palliative medicine experts. The use of structured checklists improves the disseminability of this model.

### Limitations

This study had several limitations. It was conducted at academic centers that had specific interest and experience in providing PC for patients with PDRD. Further study is needed to determine whether this intervention can be implemented in other settings. Notably, although our clinics varied in their specific clinic model, no significant differences were found in effectiveness by site, suggesting that the material covered is more important than clinic logistics. Our comparator condition represented optimized standard care (many patients with PD do not see a neurologist), and the presence of neuropalliative care at academic centers could have contaminated our standard care condition, both of which may have diminished our treatment effects. The population studied was not diverse and may have been biased toward persons interested in receiving PC. It is possible that variables other than the intervention, such as total contact time, were factors in the outcomes. As a pragmatic clinical trial, the study had broad inclusion criteria and covered many domains in our intervention. It is possible that more focused recruitment or interventions could improve certain outcomes. Finally, as this study could not be double-blinded, it is possible that patient-reported outcomes were biased.^47^

## Conclusions

The integration of PC into PDRD care holds the potential to improve outcomes, particularly for persons who are underserved by current models of care (eg, patients with advanced illness and dementia). As a new application of PC, a need exists to optimize the intervention, particularly for caregivers, and to develop models appropriate for implementation in nonacademic settings and among diverse populations. Because the PC intervention is time-intensive and resource-intensive, future studies should optimize triage tools and consider alternative models of care delivery, such as telemedicine or care navigators, to provide key aspects of the intervention at lower cost. Despite these limitations, the study’s results provide a starting point for future studies integrating PC into standard care for patients with PDRD and other chronic illnesses.

## Notes

Supplement 1. Trial Protocol

Supplement 2. **eMethods 1.** Palliative Care Needs Assessment Tool–Parkinson Disease **eMethods 2.** Detailed Description of Outpatient Palliative Care Intervention: Training and Experience of the Integrated Palliative Care Team, Logistics of Integrated Palliative Care Clinic Visits, and Goals of Integrated Palliative Care Visits and Team Member Roles **eMethods 3.** Neurologist, Social Worker, Spiritual Care, and Nurse Visit Checklists **eMethods 4.** Patient Health Care Utilization and Caregiver Health Care Utilization Questionnaires **eResults.** Additional Sensitivity Analyses, Subgroup Analyses, and Health Care Use Analyses eTable 1. Raw Differences Between Groups in Primary and Secondary Outcomes, Summary Statistics eTable 2. Differences Between Groups in Primary and Secondary Outcomes, Inferential Statistics, Unadjusted eTable 3. Snapshot Models for Patients for the 6-Week Periods Proceeding Baseline, 6 Months, and 12 Months eTable 4. Cumulative Models for Patients for 6 Months and 12 Months, Patients With Complete Data Only eReferences

Supplement 3. Data Sharing Statement
