# Supplementary material for: Comparison of Integrated Outpatient Palliative Care With Standard Care in Patients With Parkinson Disease and Related Disorders: A Randomized Clinical Trial
Source: JAMA Neurol. 2020 Feb 10;77(5):1–11. doi: 10.1001/jamaneurol.2019.4992 (PMC7042842; doi:10.1001/jamaneurol.2019.4992)
Supplement: Supplement 1. — Trial Protocol [file jamaneurol-77-551-s001.pdf]

Clinical Trial Protocol for:

Does Outpatient Palliative Care Improve Patient-centered Outcomes in Parkinson's Disease?

Benzi M. Kluger, MD, MS, Study PI. Departments of Neurology and Medicine, University of Colorado School of Medicine, Aurora, Colorado
Jean S. Kutner, MD, MSPH, Palliative Medicine Consultant. Department of Medicine, University of Colorado School of Medicine, Aurora, Colorado
Stefan H. Sillau, PhD, Biostatistician. Department of Neurology, University of Colorado School of Medicine, Aurora, Colorado
Diane L. Fairclough, DrPH, Senior Biostatistician. Department of Biostatistics and Informatics, Colorado School of Public Health, Aurora, Colorado
Jacqueline Jones, PhD, Qualitative and Mixed Methods. College of Nursing, University of Colorado, School of Medicine, Aurora, Colorado
Laura T. Palmer, BS, Project Coordinator. Department of Neurology, University of Colorado School of Medicine, Aurora, Colorado
Janis M. Miyasaki, MD, Site-PI. Department of Neurology, University of Alberta, Edmonton, Alberta, Canada
Maya Katz, MD, Site-PI. Department of Neurology, University of California San Francisco, San Francisco, California
Nicholas B. Galifianakis, MD, MPH, Site-co-PI. Department of Neurology, University of California San Francisco, San Francisco, California
Steven Z. Pantilat, MD, Palliative Medicine Consultant. Department of Medicine, University of California San Francisco, San Francisco, California

University of Colorado Denver, Anschutz Medical Campus

PCORI Grant # IHS-1408-20134

Clinical trials identifier: NCT02533921

TABLE OF CONTENTS **Page**

Note to NEJM Readers	1
A. Background	2
1. Background and Significance	2
2. Research Questions and Gaps	3
3. Specific Aims	3
4. Preliminary Data and Feasibility	3
5. Important Innovations of the Study and Intervention	6
B. Patient and Stakeholder Engagement	7
1. Planning the Study	7
2. Conducting the Study	8
3. Disseminating Study Results	8
C. Methods	8
1. Study Overview	9
2. Study Setting	9
3. Participants	10
4. Interventions and Comparators or Controls	12
5. Study Outcomes	14
6. Sample Size Calculations and Power	17
7. Timeframe for the Study	19
8. Data Collection and Sources	19
9. Analytical and Statistical Approaches	20
10. Changes to the Original Study Protocol	26
D. Study Management across Sites	31
E. Potential Problems and Alternative Strategies	31
F. Future Directions	32
G. Human Subjects	33
1. Risks to Human Subjects	33
2. Adequacy of Protection Against Risks	35

3. Potential Benefits of the Proposed Research to Human Subjects and Others 36
4. Importance of Knowledge to be Gained 36
5. Data and Safety Monitoring Plan 37
6. ClinicalTrials.gov Requirements 37

H. References 37
I. Acknowledgements 41
J. Related Publications 43

LIST OF TABLES

Table 1: Interdisciplinary Palliative Care Visit Checklist 13
Table 2: Outcomes of Interest to Patients, Caregivers & Other Stakeholders 16
Table 3: Interview Domains and Sample Questions 17

LIST OF APPENDICES

Appendix 1: Palliative Care Needs Assessment Tool – Parkinson's Disease 42
Appendix 2: Neurology Checklist – Initial Visit 45

NOTE FOR NEJM READERS

We have included the complete protocol for this study. Some aspects of this study such as qualitative interviews and mixed methods results are not presented in this manuscript due to space limitations and will be published separately.

A. Background

A.1. Background and Significance: Parkinson's disease (PD) is the second most common neurodegenerative illness affecting 1-2% of adults over age 65 representing approximately 1.5 million Americans.[1] With the aging of the US population this figure is expected to double by 2040.[2] While PD is traditionally defined by its motor symptoms (e.g. tremor, slowness), non-motor symptoms including pain, depression, fatigue, and dementia are extremely common and contribute significantly to quality of life (QOL) and disability.[3] For example, over the course of the illness up to 80% of individuals with PD will develop dementia.[4] PD is currently ranked as the 14th leading cause of death in the US and this may be an underestimate as PD contributes to other causes of death such as pneumonia and falls.[5,6] People living with PD are also five times more likely to be placed in a nursing home and die in hospitals significantly more often than their age-matched peers.[7,8] In addition to the direct impact of PD on patients, it also is associated with significant effects on their family caregivers including high rates of stress, increased incidence of depression and increased mortality.[9,10]

Despite these advances in our understanding of the burden of PD on patients and caregivers, clinical care continues to be driven by a chronic illness model predominantly focused on managing motor symptoms through a patient-physician dyad.[11] Potential gaps in this model include addressing psychosocial and spiritual concerns; recognition and management of non-motor symptoms; advance care planning; and caregiver support.[11,12] Palliative care focuses on the relief of suffering through the assessment and treatment of physical, psychosocial, and spiritual issues.[13] While traditionally associated with cancer, palliative approaches have been successfully applied to several chronic progressive illnesses including heart failure and pulmonary disease.[14,15] Several recent systematic review articles suggest palliative care approaches may be beneficial for PD patients and that PD patients and their family caregivers have unique palliative care needs compared with other populations (e.g. cancer).[16,17] In line with these articles, a few centers in the US and Canada have begun offering interdisciplinary outpatient palliative care for PD patients including the three sites heading this proposal.

A.2. Research Questions and Gaps: As noted in recent reviews,[18] there are significant gaps in the evidence needed to support widespread adoption of this care model:

1) A need for randomized controlled comparative effectiveness trials of palliative care interventions in PD to determine whether such care does in fact improve patient-centered outcomes versus standard care.
2) Data to guide referral and selection of appropriate patients for this time and resource intensive intervention.
3) Data to standardize and optimize which services are provided and how they are delivered, optimally with direct input from PD patients and their caregivers.

This research has a significant potential to impact clinical care for persons living with PD and related disorders by providing the first empirical evidence of whether this model of care improves patient or caregiver outcomes. Positive results would influence care by: a) providing greater support for the assertion that PD and related disorders are conditions that merit PC; b) demonstrating that patient quality of life (QOL) and caregiver burden can be improved above current standards of care through a PC approach; c) providing data to optimize this care including characteristics of persons most likely to benefit from this care; and d) identifying a replicable model of care that could be implemented in other sites.

A.3. Specific Aims: Aim 1: Determine whether interdisciplinary outpatient palliative care improves patient QOL or caregiver burden in Parkinson’s disease (PD) patients versus standard care with a neurologist. We *hypothesized* that PC would improve both patient QOL and caregiver burden by systematically addressing issues that contribute to patient and caregiver suffering.

Aim 2: Identify characteristics of PD patients most likely to benefit from a palliative care approach. We *hypothesized* that patients and caregivers with worse QOL would perceive greater benefits from this additional support.

Aim 3: Interview patients and caregivers to elicit their direct input on what palliative care services are most helpful, what additional services may be needed, and preferences for service delivery. We *hypothesized* that patients and caregivers will provide insights to optimize our model of care to better meet patient and caregiver needs.

A.4. Preliminary Data and Feasibility

1) Effectiveness of Outpatient Palliative Care for PD: Dr. Janis Miyasaki (University of Alberta site PI) and colleagues published a case series of 109 PD patients who had received outpatient palliative care from her team.[19] This study showed a significant improvement in symptom burden as measured by the Edmonton Symptom Assessment Scale PD (ESAS-PD) between their baseline (mean $\pm$ STD = 56 $\pm$ 19) and first follow-up visit (40 $\pm$ 17; paired t-test $p < 0.0001$). Significant improvements were seen in many individual symptoms including pain, anxiety, drowsiness, and dysphagia. Although there was no control group, these patients were largely referred by their treating neurologists because of limited success with standard care. Moreover, Dr. Miyasaki and Dr. Benzi Kluger (PI) published data showing that 80% of PD patients participating in outpatient palliative clinics died at home and less than 10% died in a hospital.[20] While there was no control group, historical data shows over 50% of PD patients typically die in hospitals and less than 10% die at home.[8]

2) Patient and Caregiver-Centeredness of Intervention: Dr. Kluger (PI) obtained a Clinical Effectiveness and Patient Safety Grant through the University of Colorado Hospital in July 2013 to optimize our outpatient palliative care clinic with the non-negotiable goal of achieving high quality, patient-centered care. We collected data through surveys and phone interviews with patients and caregivers and made ongoing adjustments in our clinic based on feedback received. Specifically, we revised clinic processes by: 1) Providing written information about palliative care and our clinic to patients prior to their first visit; 2) Clarifying team member roles to reduce redundancy and ensure coverage of key issues; 3) Providing written instructions to patients in real time from each team member; and 4) Following up on visit recommendations with phone calls within two weeks. We tracked patient and caregiver satisfaction through the course of this project and obtained mean scores of at least 9 (on a 10 point scale) on all items by the end of our 1-year improvement period including overall satisfaction, answering their questions, and likelihood of referring other patients to our program.

3) Experience and Standardization of Intervention: Dr. Miyasaki created the first outpatient palliative care program in the world dedicated to PD patients and their families at the University of Toronto in 2008. From the time of its inception this program became a model

for other centers including the University of Colorado Denver (UCD) and University of California San Francisco (UCSF) and an integral part of care for patients with advanced PD in Toronto addressing goals of care, refractory nonmotor symptoms, caregiver support, advance care planning, and spiritual wellbeing. Dr. Nicholas Galifianakis (UCSF Co-I) and colleagues presented findings from the development of their PD palliative clinic and first 25 patients at the 2012 International Congress of Parkinson's Disease and Movement Disorders.[21] Relevant findings from this presentation include a description of their intervention and the most common referral reasons including goals of care, management of non-motor symptoms, and caregiver support. Although our programs have always been similar, we have shared experiences, forms, and other materials in the planning of this proposal to ensure standardization across sites.

4) Palliative Care Needs of PD Patients and Caregivers – Quantitative Data: Dr. Kluger (PI) obtained a pilot grant in June 2013 from the Veterans Affairs Medical Center (VAMC) to perform a cross-sectional study of 90 patients with PD and 45 patients with advanced cancer (inoperable or metastatic) to determine whether palliative issues contributed to QOL in PD and compare palliative needs in PD to those with cancer. Key findings of this study relevant to this proposal include: 1) PD patients across all stages of the disease had similar or higher levels of symptom burden, grief, depression, QOL impairments, and caregiver burden as advanced cancer patients; 2) Palliative specific issues (e.g. grief, spiritual wellbeing) were associated with QOL even when controlled for motor severity and depression; 3) The majority (80%) of PD patients felt advance care planning should begin early in the disease process; 4) Caregiver burden was driven largely by factors not well addressed in current models of care including financial concerns, grief, depression, and patient functional status; 5) Higher medication use was associated with high caregiver burden and reduced patient QOL even when controlling for disease stage; and 6) Components of symptom burden differed significantly between cancer and PD patients (e.g. higher psychiatric symptoms in PD population).[22]

5) Palliative Care Needs of PD Caregivers: Drs. Kluger and Jones (co-I) obtained a grant in September 2013 through the University of Colorado's National Institute on Aging funded

Palliative Care Program (1 K07 AG030337-01A2: PI - Kutner) to perform qualitative interviews and focus groups with PD patients and their caregivers with the goal of creating a patient-centered model of palliative care for PD. Key findings from over 60 hours of interviews from 45 patients, caregivers, and support groups[23,24] relevant to this proposal include: 1) Both patients and caregivers expressed significant concerns about their future ranging from what symptoms to expect to finances; 2) PD challenged many patients' sense of roles and identity; 3) Spirituality was a significant source of strength for many patients; 4) PD patients and caregivers identified many needs not met with current models of care including greater education, particularly regarding what to expect in the future; 5) Patients often under-reported symptoms to their physicians for fear of having their medications increased; 6) Patients and caregivers expressed significant openness to team-based outpatient approaches to addressing currently unmet needs; and 7) Both patients and caregivers felt that key components of palliative care (e.g. spiritual counseling, frank discussions about future needs) would be a welcome addition to current models of care.

6) <u>Clinical Research Experience in PD</u>: Dr. Kluger has experience as PI in PD trials including serving as co-PI in a 3-site investigator-initiated study of rasagaline for fatigue[25] and PI for a single site study of acupuncture for fatigue recruiting 95 participants in 2.5 years.[26] Dr. Katz (UCSF site PI) has experience in PD clinical trials and will work closely with Dr. Jill Ostrem (co-I) an experienced clinical trialist. Dr. Miyasaki (UA site PI) similarly has a long history of serving as a site PI in multisite clinical trials.

Of note, palliative care as prescribed in the "intervention arm" of this trial is already available to and being utilized with PD patients at all sites. The "comparator arm" is meant to reflect the current standard of care in the community at large. Thus this trial represents a comparison of effectiveness of two currently-available standards of care.

A.5. Important Innovations of the Study and Intervention

Important innovations of the study and intervention include:

a) Application of a PC approach in the outpatient setting to a non-cancer population.
b) Participant inclusion is based on perceived need rather than prognosis or disease-centered criteria of advanced illness.

c) Inclusion of a heterogeneous population reflective of real-world practice including patients with/without dementia and with/without caregivers.
d) Use of the Quality of Life: Alzheimer's Disease (QOL-AD)[27] and recently developed Needs Assessment Tool - Parkinson's Disease[28] and Edmonton Symptom Assessment Scale – Parkinson's Disease (ESAS-PD)[29] to measure QOL, PC need, and symptom burden respectively.
e) Use of mixed methods to more fully capture the patient and caregiver experience as well as to find means of optimizing our intervention.
f) Use of checklists to improve the replicability and implementation potential of our intervention.
g) Use of a hybrid model of disease-specific PC that maximizes collaboration and applied expertise of both palliative medicine specialists and neurologists. This model is reflective of current neuropalliative care programs.
h) Pragmatic study design that places higher priority on capturing current practices of care rather than holding providers to an inflexible model of care.

B. Patient and Stakeholder Engagement

B.1. Planning the Study: Our patient advisory committee members were actively involved in planning this study beginning with our research question that arose out of a conversation between the PI and our lead patient advisor, Kirk Hall. Kirk Hall assembled our initial patient and caregiver council that was involved with reviewing our letter of intent prior to its submission, as well as our full grant. Specific feedback was elicited regarding choice of outcome measures, recruitment, study population, and potential impact. Patient advisors tested and gave their perspectives on all study procedures, including intervention and data collection, to optimize procedures and flow prior to participant enrollment.
Regarding other stakeholders, the Parkinson Foundation sponsored two international working group meetings on Palliative Care and Parkinson's Disease (including members of our patient and caregiver council) which provided substantive help in framing the gaps of the field, patient and caregiver needs, and a vision for future implementation.

B.2. Conducting the Study: Each study site had at least one dedicated patient team-member who assisted researchers with recruitment (including speaking at support groups and other events), communication with study subjects, review of study outcomes, review of dropouts, review of any adverse events, and assistance with any study modifications if needed. Patient advisors were involved in reviewing and creating materials provided to patients, including a feature story on our advisory council in one of our study newsletters. The advisory council was also very active in our qualitative study including ongoing review of transcripts and modifications to our interview guide. Our advisory committee, including patients and other stakeholders, was updated on study progress every six months and provided helpful suggestions and confirmation of changes to meet recruitment goals.

Regarding other stakeholders, the Parkinson Association of the Rockies (PAR) was instrumental in supporting recruitment for this study and awareness of our work through their newsletter, support groups and other events.

B.3. Disseminating Study Results: All stakeholders were invited to attend our final study meeting either in person or via teleconference. At this meeting we presented all study results and distributed summaries of these results as well as a list of papers published, submitted, in-progress and in-planning. All stakeholders were reminded that they can gain access to our complete de-identified database and make suggestions for papers or analyses. Our patient and caregiver advisory council actively participated in our qualitative analyses and each of our qualitative papers included at least one patient or caregiver. Patients and caregivers have presented results related to this study and are actively working on talks to give to community support groups, as well as to distribute to other support group leaders.

Regarding other stakeholders, the Parkinson Foundation is open to disseminating educational materials for patients and caregivers based on the results of this study. They are also very interested in using our intervention model as a new standard of care for their centers of excellence, and we are planning on collaborating to submit an implementation/dissemination grant.

<u>C. Methods</u>

C.1. Study Overview: We will recruit 210 PD patients and their family caregivers (if available) with moderate to high palliative care needs across three sites. Patient-caregiver dyads will be randomized in a 1:1 fashion to either: 1) Standard care, defined as including both a primary care provider (PCP) and neurologist; or 2) Standard care augmented by an outpatient interdisciplinary palliative care team. Participants will undergo a battery of measures at their baseline visit to assess QOL, disease severity, mood, symptom burden, caregiver burden, recent ER and hospital visits, and living situation. Outcomes measures were collected at 3, 6, 9, and 12 months.

Aim 1: Determine whether interdisciplinary outpatient palliative care improves patient quality of life (QOL) or caregiver burden in Parkinson's disease (PD) patients versus standard care with a neurologist. For Aim 1 mixed regression models will be used to assess our primary comparative effectiveness outcome: the difference in change on the QOL-AD and ZBI between the intervention and standard care groups at 6 months. We *hypothesize* that PC will improve both patient QOL and caregiver burden by systematically addressing issues that contribute to patient and caregiver suffering.

Aim 2: Identify characteristics of PD patients most likely to benefit from a palliative care approach. For Aim 2 our primary analysis will be built on regression models from Aim 1 to determine what baseline patient and caregiver characteristics are most strongly associated with caregiver and patient improvement in the intervention arm. We *hypothesize* that patients and caregivers with worse QOL will perceive greater benefits from this additional support.

Aim 3: Interview patients and caregivers to elicit their direct input on what palliative care services are most helpful, what additional services may be needed, and preferences for service delivery. For Aim 3 we will perform qualitative interviews on a subset of patients (N = 14) and caregivers (N = 20) in the intervention arm to assess patient/caregiver satisfaction and identify opportunities to improve services provided and delivery methods. We *anticipate* that patients and caregivers will provide valuable insights beyond quantitate surveys to optimize our model of care to better meet patient and caregiver needs.

C.2. Study Setting: The setting for this study will be three academic neurology clinics (the University of Alberta; the University of Colorado Denver, Anschutz Medical Campus - UCD; and

the University of California San Francisco – UCSF) and their surrounding communities. Study sites were chosen on the basis of the following criteria: 1) Experience in providing outpatient palliative care services to PD patients: The three sites chosen included the director of the first PD palliative care clinic in the world (Dr. Miyasaki) and two of the first such clinic sites in the US (Colorado and UCSF); 2) Presence of large PD population for recruitment: UCSF, UCD and the University of Alberta are the leading tertiary hospitals for major metropolitan areas and serve large catchment areas, including rural locations; 3) Willingness to collaborate with community providers, patients, and other stakeholders; 4) Interest and passion in palliative and patient–centered care; and 5) Experience with clinical research and multisite trials in PD.

The populations to be served by this trial are representative of the PD patients we anticipated would be most likely to use this care model, namely patients with moderate to high palliative care needs who are accessing care with general neurologists or movement disorders specialists. The sites chosen provide a broad demographic base, which is similar to the US as a whole in terms of racial and ethnic breakdown and includes persons from inner city and rural environments.

The study sites chosen also provide real-world settings relevant to future implementation efforts. These sites had already acted as models that had served to inspire other palliative clinics for PD in practice or in development. The University of Alberta site had been actively developed by an experienced clinician in this field (Dr. Miyasaki) and provided data on this implementation process potentially important to other new sites. Data from Aims 2 and 3 are specifically being collected with the goals of informing future implementation and dissemination efforts, including the potential for designing less intensive programs meeting essential needs for rural and community settings.

C.3. Participants: Participants will be *identified and recruited* for this study through three main venues: 1) Referral from investigators' clinics or their colleagues at primary study sites; 2) Referral from community physicians who were notified of the study through existing personal contacts and advertisements; and 3) Self-referral by patients who learned of the study through community organizations, talks, advertisements, or websites (e.g. clinicaltrials.gov). To ensure inclusion of community and rural participants, each study site will partner with community

neurologists and regional patient organizations which provide services to patients in rural locations. Study sites also provided services through inner city clinics serving indigent populations, which also tend to provide services to a larger proportion of minority populations. We will *select* participants using the following inclusion and exclusion criteria:

Inclusion Criteria: Potential participants must be fluent English speakers, over age 40, and meet UK Brain Bank criteria for a diagnosis of probable PD[30] or have another neurodegenerative cause of parkinsonism (progressive supranuclear palsy, corticobasal degeneration, Lewy Body Dementia of multiple system atrophy). Although PD and related disorders may be seen in individuals under age 40, it is more frequently related to specific genes and may display different clinical features than typical idiopathic PD. Patients must be at high risk for poor outcomes as identified by the Palliative Care Needs Assessment Tool (PC-NAT)[28] modified for PD, which screens patients on the basis of social factors (e.g. presence of caregiver), disease severity, and symptom burden. Caregivers, when present, will be identified by asking the patient: "Could you please tell us the one person who helps you the most with your PD outside of the clinic?" For patients with severe dementia, family caregivers can be self-identified and can be included in the study, even if the patient has communication limitations, in order to obtain data relevant to these highly vulnerable and underrepresented patients. Notably, our primary patient outcome measure has been used in PD patients with dementia and can be used with proxy caregiver reporting with good intraclass correlations.[31]

Exclusion Criteria: Patients will be excluded if any of the following are present: 1) Immediate and urgent palliative care needs (these patients will not be randomized and will be offered appropriate services immediately); 2) Unable or unwilling to commit to study procedures including randomization, study visits, or addition of neurologist if in the standard care arm if not currently seeing a neurologist; 3) Presence of additional chronic medical illnesses that require palliative services (e.g. metastatic cancer); or 4) Already receiving palliative care and/or hospice. We have purposefully kept our inclusion/exclusion criteria broad to allow for greater generalizability of results and to ensure inclusion of potentially under-represented subgroups. Potential participants will be *first contacted* by phone where they will be informed of the basic purpose of the study, their potential involvement, and inclusion criteria. Participants will

undergo a scripted phone screening that includes queries on source of referral (e.g. from university or community physician, support group talk, website) and, if excluded at this stage, reasons for exclusion. Following phone screening, potential participants will proceed to an in-person visit for purposes of consent, further screening, and baseline data collection. Reasons for exclusion, including potential participants' reasons for declining to participate even if eligible, will be recorded as part of this visit.

Participants will be randomized in a 1:1 ratio to either the palliative or standard care group through a randomized block design stratified by site, presence of dementia (using standard criteria for PD-related dementia[32]), and presence of a caregiver (patients can be enrolled without caregivers). Due to the nature of this study, participants and clinicians cannot be blinded to group assignment. We chose not to add to study expense with blinded assessment as our primary outcomes are self-administered patient-reported outcomes.

C.4. Interventions and Comparators or Controls: Comparator: Our standard care arm will consist of care from the patient's primary care physician (PCP) and a neurologist. We consider this the current standard on the basis of recent evidence showing that PD care with a neurologist results in better outcomes (e.g. falls, mortality) than care with a PCP alone.[33] Patients not currently seeing a neurologist randomized to this arm will have appointments with a neurologist covered by their insurance or patient assistance programs arranged by the study team. These neurologists are board-certified and consist of either academic movement disorders specialists associated with the primary site (but not doing palliative care) or well-established community neurologists, many of whom also had completed movement disorders fellowships. Patients will be asked to see their neurologist at least every three months to match the frequency of visits in the intervention arm and saw their PCP at their typical frequency.

Intervention Arm: Our intervention will consist of Standard Care plus the addition of an outpatient interdisciplinary palliative care team. Palliative care visits will be performed in person at each site's academic university clinic (or telemedicine if needed for rural areas and/or limited transportation) every three months. Visits will last approximately 2 to 2.5 hours broken into one-on-one visits with the neurologist (60 minutes for new and 30 for returns) and 30-minute visits with all other team members (RN, social worker, chaplain). Providers will

communicate in-person or via notes during the clinic visit to increase interdisciplinary coordination and meet at the end of the day for discussion including follow-up plans. In addition, visits will be supplemented by phone calls one week after each visit to confirm understanding of team recommendations and at six weeks to check-in with patient and caregiver. Patients and caregivers can also contact the team as needed. Summaries of visits will be sent to the patient, caregiver, PCP, and neurologist and suggestions for care outside of palliative care issues will be left to the patients' Standard Care team.

The interdisciplinary team will consist of a neurologist with palliative care experience (consisting of direct experience, mentorship, and reading but without formal palliative medicine fellowship), a nurse, social worker, and a chaplain. Of note, the palliative care neurologist will play a consultative role for nonpalliative issues (e.g. PD medications) and will instead make recommendations to the patient's primary neurologist. A board-certified palliative care physician will be available at each site for coaching, phone, or in-person consultations at the discretion of the primary team and for periodic review of charts (after every 10 participants). Visits will be standardized through the use of checklists (see Appendix 2) which will be used to guide all team member visits to ensure consistency across sites as summarized in Table 1. While these checklists will not be directly accessible in the medical record, they will be used to craft documentation related to study visits. All staff except the palliative care physician will be present for in-person visits and participate in summary meetings at the end of each clinic to ensure interdisciplinary care and completion of any needed referrals or medication changes.

Table 1. Interdisciplinary Palliative Care Visit Checklist

Team Member	Issues to Address
Palliative Neurologist	- Medical history, medications and physical examination<br>- Cognitive status and testing<br>- Psychiatric symptoms (e.g. depression, hallucinations)<br>- Pain, sleep, fatigue and other nonmotor symptoms<br>- Swallowing, sialorrhea and falls<br>- Recent hospitalizations, infections or other medical issues

	- PD education relevant to disease stage including prognosis<br>- Goals of Care
Social Worker	- Caregiver distress<br>- Need for help at home/community resources<br>- Financial issues and concerns<br>- Long-term care needs
Chaplain	- Spiritual wellbeing<br>- Sources of support and stress<br>- Fear, anger and guilt<br>- Grief and demoralization
Nurse	- Advance care planning and documentation<br>- Healthcare proxy designation and documentation<br>- Wound care/skin integrity<br>- Nutritional status and diet
Palliative Care Physician	- Coaching and guidance for team<br>- Periodic review of charts from palliative perspective and coach for team<br>- Direct patient care at discretion of other team members

Measuring Content of Care: For both standard care and intervention arms, we will collect data on the frequency of primary care and neurologist visits as well as the use of other services (e.g. psychotherapy, physical therapy, etc.). In the intervention arm we additionally will assess fidelity with our standardized visit checklists and completion of study related phone calls to participants.

C.5. Study Outcomes: Our co-primary outcomes were change in patient QOL, measured using the QOL-Alzheimer's disease (QOL-AD) scale and change in caregiver burden, measured using the Zarit Burden Inventory (ZBI), between the intervention and standard care groups at six months. The primary goal of palliative care in general, and our intervention specifically, is to improve patient QOL and to reduce patient suffering and caregiver distress. QOL refers to a

persons' self-assessment of overall well-being including emotional, social, physical, and spiritual or existential aspects and is one of the cornerstones of the movement towards value-based medicine of which PCORI is a key proponent.[34,35] Patient QOL is clearly important to patients and is recognized by other stakeholders including clinicians, healthcare organizations, and insurance as the single most important patient-reported outcome, in some cases overriding even survival.[34] Caregiver burden refers to adverse effects of caregiving on the caregiver's overall wellbeing and functioning.[36] Caregiver burden is meaningful to caregivers not only as a measure of distress but also a marker for increased mortality and other health issues.[10] Caregiver burden also impacts quality of care for patients, likelihood of nursing home placement and, like QOL, is being increasingly recognized as an important value indicator for interventions among chronic progressive illnesses.[37] Patient QOL and caregiver burden are thus highly relevant and appropriate outcomes for this comparative effectiveness study and should provide relevant data to inform future research, stakeholder decisions, implementation, and dissemination efforts.

The QOL-AD was chosen for this study for several reasons including its brevity, validation in dementia including PD dementia, validated proxy reporting, sensitivity to change, and coverage of issues identified to be relevant to PD patients and caregivers in qualitative interviews, including with our patient advisory council.[24,38-41] While the QOL-AD has been shown to be responsive to disease progression and the effect of interventions, the minimal clinically important difference (MCID) has not been defined for this instrument.[42] The ZBI is the most commonly used self-report measure of caregiver distress, including in PD.[9,43] The ZBI is well validated in terms of psychometric properties and is notably insensitive to variations in age, gender, socioeconomic status, or locale indicating that it is appropriate for use in diverse and mixed populations.[44] It is also sensitive to change with interventions, including palliative care, but the MCID has not been defined.[45]

Tables 2 and 3 summarize outcome measures and relevant domains of interest to PD patients, caregivers, and other stakeholders to be assessed in this proposal. Notably, these outcomes were discussed with our patient advisors and when multiple potential measures were available for a particular domain (e.g. patient QOL) the group reviewed multiple measures with

investigators and came to a consensus on their top choice. For most measures we used the short form when available to minimize burden to participants. To evaluate quality of care we asked all caregivers and patients to complete a satisfaction survey that included items on how helpful visits were, likelihood of recommending the clinic to others, ratings of specific services, and access to care (e.g. waiting time, promptness in returning calls), as well as space for written comments. A subset of patients and caregivers in the intervention arm additionally completed semi-structured interviews to provide more detailed input as well as recommendations for optimizing services provided and delivery methods. Finally, we tracked certain key events (hospitalizations, emergency room visits, nursing home placement, deaths, and place of death) that are of clear importance to patients and caregivers and that could have been impacted by our intervention. We specifically predicted that improved patient and caregiver support may reduce these distressing events and outpatient interventions in other populations have demonstrated some survival benefits.[46] In addition to being of clear interest to patients and caregivers, our chosen outcomes provided critical information regarding the comparative effectiveness of our care model to other stakeholders including national PD organizations, health care providers, insurance companies, hospitals, and patient advocacy groups.

Table 2. Outcomes of Interest to Patients, Caregivers and Other Stakeholders

**Outcome Measure**	**Domain of Interest**
Quality of Life Alzheimer's Disease (QOL-AD)[47]*	Patient Quality of Life (Primary Outcome)
Zarit Burden Interview (ZBI)[43] *	Caregiver Distress (co-Primary Outcome)
Edmonton Symptom Assessment Scale revised for Parkinson's disease (ESAS-PD)[48]*	Patient Overall symptom burden
Hospital Anxiety and Depression Scale (HADS)[49]*	Patient and Caregiver Mood
Parkinson Disease Questionnaire (PDQ-39)[50]*	Patient Health Related Quality of Life

Functional Assessment of Chronic Illness Therapy-Spiritual Wellbeing (FACIT-SW)[51]*	Patient and Caregiver Spiritual Wellbeing
Prolonged Grief Questionnaire (PG-12)[52]*	Patient and Caregiver grief (sense of loss)
Unified Parkinson Disease Rating Scale (UPDRS)[53]	Patient Motor symptom Severity
Semi-structured Qualitative Interview (see Table 3)*	Patient and Caregiver recommendations for optimizing services provided and delivery methods
Hospitalizations, emergency room visits, home health services, nursing home placement	Health Service Utilization Survey
• Indicates patient-reported outcome	

Table 3. Interview Domains and Sample Questions

Interview Domain	Sample Questions
Satisfaction with Services Provided	What services provided by the palliative care team did you find helpful? Were there any services provided you did not need?
Optimization of Services	Were there any services which you did not receive from the palliative care team or others that you think would be helpful?
Care Delivery	How would you prefer (service discussed earlier) to be provided? For example, in person visits versus phone call.
Other Services	What additional services could be provided to improve you quality of life?
Timing of Services	When in the course of PD do you think palliative services should be started?
Who to Refer	Thinking of yourself and other people you know with PD, who do you think would benefit most from these services?

C.6. Sample Size Calculations and Power: Our target sample size was 210 patients and 180 caregivers, we accrued 210 patients and 175 caregivers. For our primary patient outcome of

change in the QOL-AD at six months, allowing for up to 15 dropouts per group (19 total were observed) by the 6-month time point, a two sample T-test with 90 samples per treatment group (180 total) will detect a difference in our continuous outcomes equal to half the standard deviation with 90% power and an alpha level 0.05. A difference of half the standard deviation for QOL in PD has been suggested in prior studies to be clinically important and detectable with change over time or with outpatient interventions.[54-56] For our primary caregiver outcome of change in the ZBI at six months, allowing for up to 12 dropouts per group, a two sample T-test with 78 samples per treatment group (156 total) will detect a difference in our continuous outcomes equal to half the standard deviation with 88% power and an alpha level 0.05. As MCID have not been established for the QOL-AD or ZBI we powered this study to be able to detect a moderate effect size of 0.5 times the within group standard deviation. Notably, prior studies of palliative interventions have found effect sizes of 0.5 SD/mean and greater.[45] The sample size also enabled us to fit models with a large number of parameters and as described below, the longitudinal mixed models and linear time functions increased our efficiency.

For a longitudinal model with non-linear time trajectories, the difference in area under the change from baseline curve can summarize treatment effect across all times. If the true time trajectory is linear, and we model it as a piece-wise linear function, with a common intercept and knots at each time point, and the outcome is difference in area under the change from baseline curve, then a sample size of 25 per treatment group (50 total) would achieve 90% power with an alpha level of 0.05 and an effect size equivalent to 50% of the standard deviation at six months (6 sd*months of area, 0.5 sd on average). A sample size of 90 per treatment group (180 total) would be virtual certain to detect a difference of 50% of standard deviation at six months (6 sd*months of area, 0.5 sd on average) for very small alpha levels, and so a Bonferroni correction could be applied to handle multiple comparisons. A sample size of 90 per treatment group (180 total) would achieve more than 90% power with an alpha level of 0.05 and an effect size equivalent to 30% of the standard deviation at six months (3.6 sd*months area, 0.3 sd on average). For a test of mean difference in change from baseline at six months, a sample size 64 per treatment group (128 total) would achieve 90% power with an alpha level of 0.05 and an effect size equal to 50% of the standard deviation. A sample size of 90 per group

(180 total) would achieve 97% power. The test would have more than 90% power even after a Bonferroni adjustment for four tests. Alternatively, 90 samples per group (180 total) could detect an effect equal to 42% of the standard deviation with 90% power and an alpha level of 0.05. All sample size calculations for longitudinal data models assume a compound symmetric covariance structure and a correlation of 0.5 for repeated measures on a participant which is relatively conservative for QOL measures.[57]

C.7. Time Frame for the Study: Our intervention period was 12 months, with our primary outcome assessed at 6 months and follow-up visits and assessments to occur every three months during the intervention year. We chose one year of follow up for this study and intervention as a reflection of our underlying model of palliative care for a chronic illness whose progression is typically measured in years.[58] We chose the six month time point for our primary outcome as a pragmatic balance of several factors including time needed to build trust and rapport between a new clinical team and patients; our clinical experience and input from patients through quality improvement projects of when both patients and caregivers might experience benefit; a balance between accumulating benefits of the intervention and clinical progression; and a balance between accumulating benefits and study drop-outs. We chose quarterly follow-ups for our intervention as a reflection of current practice patterns in our clinical practices, and other groups who offer outpatient palliative care for PD and chose quarterly outcome assessment to track changes associated with these visits.

C.8. Data Collection and Sources: For participants in the intervention group, we combined data collection visits with clinical care to minimize transportation and time burden. If this was not possible for participants' schedules or if they missed clinical intervention appointments, our coordinators reached out to participants to set up data collection. To minimize missing data and increase participation we offered multiple means of completing study outcomes including in-person visits, online data completion, mailed forms, telephone, and telemedicine visits. These could be combined (e.g. participant could do online data entry for most forms but do telemedicine for clinician administered portions) and, in recognition of the already high time burden for many patients and caregivers in medical-related tasks, we prioritized outcome measures to allow participants to do abbreviated reporting as they were able, including

collecting only our co-primary outcomes. For participants in the standard care group, we followed the same procedure including scheduling data collection around clinic appointments if they were receiving neurology, primary, or other care at our academic facilities.

If participants elected to withdraw from the study we collected their reasons for withdrawal. If participants missed or were out of window for study procedures, attempts were made to contact participants to determine the reason(s) for missing study procedures. If participants could not be reached through multiple means (e.g. phone, email) over a reasonable timeframe and with multiple attempts, they were deemed simply lost to follow-up.

C.9. Analytical and Statistical Approaches

C.9.a. AIM 1: The main objective of the study was to determine the comparative effectiveness of interdisciplinary outpatient palliative care versus standard care with a neurologist for PD regarding effects on patient QOL and caregiver burden. Participants were randomized in a 1:1 ratio to either the palliative or standard care group through a randomized block design stratified by site, presence of dementia (using standard criteria for PD-related dementia[32]) and presence of a caregiver (patients did not require a caregiver for inclusion). This randomization strategy was chosen to minimize potential confounding effects in this relatively small sample based on time of study entry, site differences, and clinical characteristics expected to influence outcome of disease severity and dementia. The primary outcome variables were the QOL-AD scale and the Zarit Burden Interview (ZBI).[50,59] Secondary outcomes (see Table 2) included scales for symptom burden, mood, and spiritual wellbeing, as well as occurrence of key events (e.g. hospitalizations, nursing home placement). For patient outcomes, controlling covariates included: site, gender, age, disease duration, presence of caregiver, MOCA score (baseline), Hoehn and Yahr (baseline) (< 3 vs >= 3), race (Caucasian vs Not Caucasian), Married (yes vs no), Education (less than college degree vs college degree or more). For caregiver outcomes controlling covariates included: site, caregiver gender, caregiver age, caregiver sharing the same residence as patient (yes vs no), duration of caregiving, patient MOCA score (baseline), patient Hoehn and Yahr (baseline) (< 3 vs >= 3), caregiver race (Caucasian vs Not Caucasian), caregiver married (yes vs no), caregiver education (less than college degree vs college degree or more. Explanatory variables were checked for randomization and multicollinearity. Successful

randomization should minimize imbalances between treatment groups, but including them in the model can adjust for lingering confounding effects and account for variance in the outcome variable.

The outcomes were modeled with mixed regression model analysis, with correlation for repeated measures, at baseline and at 3, 6, 9, and 12 months. The empirical covariance estimator may be used to safeguard against model misspecification. The models control for time, interaction between treatment and time, and potential baseline confounders, including gender, age, depression, and disease severity. The default model had linear time trajectories for each treatment group, and linear functions for baseline controlling covariates. The linearity assumptions were checked. Non-linear functions could be approximated with piece-wise linear functions or transforms. The main contrasts will be the difference in areas under the fixed effect change from baseline curves for the palliative and treatment control groups, and the mean difference in change between six months and baseline between the groups. Secondary tests were conducted for the mean difference in change from baseline between the groups at the 3, 9, and 12-month time points. The contrasts, controlled for all covariates, were tested for significance using T-tests and F-tests, with an alpha level of 0.05. The effect estimates, 95% confidence intervals, and p values are reported. In the process, the interaction between time and treatment were tested. If the time trajectories are linear, then all tests are stochastically equivalent to the time by treatment interaction test with one parameter. Binary outcomes and count outcomes were analyzed with logistic regression and Poisson regression respectively. Generalized mixed models account for repeated measures. We performed subgroup analyses for patients with dementia, severe disease, and depression and may perform exploratory subgroup analyses depending on primary results. Missing data approaches are described below**.**

C.9.b. Aim 2 (Covers Heterogeneity of Treatment Effects – HTE): A secondary objective of this proposal is to explore factors modifying the effect of palliative care compared with standard treatment which may serve as clinical indicators for which patients to refer and the design of future studies. This analysis specifically included the following patient characteristics: age, gender, medical comorbidities (Charlson Comorbidity Index[60]), presence of caregiver, cognitive status and depression. We also examined caregiver/socioeconomic characteristics including

baseline caregiver distress, income, race/ethnicity, and enrolling site. Starting from the models in Aim 1, the effect modifiers were introduced as interaction terms, so the time function for each treatment of group can be different for different levels of the interacting variable. The model with the interaction was tested against the no interaction model. Interaction effects at individual time points were also tested. The treatment effects for different levels of the interacting variable are reported. For categorical interacting variables, the treatment effect is reported at all levels of the interacting variable. For continuous interacting variables, the treatment effect is reported at pre-specified values of the interacting variable (e.g. dementia and mild cognitive impairment cut-points for the Montreal Cognitive Assessment[61]), natural boundaries (e.g. decades for age), or quartiles (e.g. for baseline caregiver burden). Categories were collapsed when necessary and justified to prevent sparsely populated cells. An overall test for treatment effect across the different levels of the interacting variable was performed as a multiple comparisons safeguard. Each interaction effect was considered in separate models. Since the study is relatively small for and lacks the power to investigate effect heterogeneities with precision, Aim 2 was largely exploratory and descriptive unless very large effect sizes were detected.

Regarding power, in a situation where the treatment effect interacted with a binary variable and the sample was evenly distributed among the four combination of treatment and the interacting binary variable, achieving 80% power with an alpha level of 0.05 and 44 samples in each combination (176 total) would require a difference in treatment effect equal to 85% of the standard deviation. Alternatively, a sample of 180 total would be able to detect an increase in the r^2 statistic of the regression model of 0.04 with 80% power and an alpha level of 0.05. A test for the treatment effect within a subgroup of half the sample (90 out of 180, 45 palliative care and 45 standard care patients), with an alpha level of 0.05, would require an effect sizes of 60% and 70% of the standard deviation to achieve 80% and 90% power respectively.

Longitudinal mixed models and linear time functions, however, would increase this efficiency. For a situation where the treatment effect interacted with a binary variable and sample was evenly distributed among the four combinations of treatment and the interaction binary variable, a sample size of 45 in each combination (180) total could achieve 90% power with an

alpha level of 0.05 and a difference in treatment effect equal to 40% of the standard deviation. A test for a treatment effect within a subgroup of half the sample could achieve 90% power with an alpha level of 0.05 and a treatment effect equal to 30% of the standard deviation. All sample size calculations for longitudinal data models assume a compound symmetric covariance structure and a correlation of 0.5 for repeated measures on a participant. Adjusting for multiple comparisons would make the individual tests more conservative. However, the linear model eliminates the need for multiple tests across time points.

C.9.c. Aim 3 (Qualitative) Data Analysis: To meet the primary goal of optimizing and evaluating outpatient palliative service models in PD we utilized an iterative, inductive, and deductive toolkit of analytical strategies drawing on field notes and memoing, qualitative content methods of analysis, consultative and reflexive team analysis, and audit and member checking as performed by members of mentorship team and Scientific Advisory Board.[62-64] ATLAS.ti was used for data organization and management. Analysis commenced with the first participant and proceeded alongside data collection, informing, and modifying our interview guide and recruitment. The words participants use, their beliefs, needs and preferences are described. Initial coding was done independently by our qualitative researcher and her research assistant who then discussed codes, established inter-code reliability, and created an initial master code list. Drs. Kluger and/or Kutner were available for adjudication if coding differences persisted. The code list was revisited and revised with continued data collection and with input from the multidisciplinary team (Scientific Advisory Board). Text within and between codes was compared to develop themes. Tables were developed displaying counts of codes to search for patterns, similarities, and differences between caregivers and patients. Secondary analyses developed similar tables to determine whether there were potential differences in needs based on age, gender, race, disease stage, or cognitive status. Through this process we developed themes which were modified based on feedback from our multidisciplinary team of advisors and potentially through feedback at presentations prior to publication. Observer triangulation (using interdisciplinary team, stakeholders and patient advisors), participant triangulation (comparing caregiver and patient perspectives), and member checking (eliciting feedback on themes from subsamples of participants) were employed to increase validity.

C.9.d. Missing Data

C.9.d.1. Methods to prevent and monitor missing data (MD-1)

We took the following steps to prevent and monitor missing data:

1) Study participants were presented with full details of the study and their involvement at the beginning of the trial to avoid false expectations from participation in this study and screen out participants unlikely to be compliant with study visits.
2) Scales were collected in the same order at all visits with primary outcomes performed first to minimize data loss or inaccuracy due to fatigue.
3) Total duration of outcome assessments was 60-90 minutes per visit with allowance for breaks to minimize participant burden.
4) During the trial every effort was made to facilitate study visits and data collection including support of transportation costs and home visits by coordinators if needed.
5) Study visits could have been completed ± 1 week from actual study date (e.g. 12 week visit) to allow greater flexibility for participants regarding travel.
6) All study packets had a checklist on the front page to minimize missing forms.
7) Coordinators reviewed all forms with patient/caregivers during study visits to minimize the chance of missing pages or items.
8) Schedules of study visits were provided to participants at their first visit and reminder calls and emails were provided one week and one day prior to visits.
9) Database was set up with strict limits on values and we replicated study forms to minimize inaccurate entry and ensure consistency across sites.
10) Database was set up to flag missing data in real time.
11) Data was entered within one week of study visits to allow for timely follow-up of missing items if detected.
12) Data was verified by a research assistant independent from staff entering data to minimize missing or inaccurate entries.

The strategy was successful and the dropout rate in patients at 6months was approximately 10% (less than the rate used to determine sample size and power.

C.9.d.2. Statistical methods to handle missing data (MD-2)

Item non-response for the scales was dealt with according to the instructions of the validated scales or using the half rule. The half rule refers imputing missing items using the mean of the answered item when at least half of the items are answered.[57] Descriptive statistics have been compiled for missing scales and assessed for patterns. Logistic regression for missingness were used to demonstrate its dependency on variables. The initial analysis used the maximum likelihood for all available data for a response variable when data exists for all explanatory variables. In mixed models correlation on repeated outcomes provides some information on the times with missing outcome values. The data will be assumed missing at random. As a sensitivity analysis, joint models combined the longitudinal outcomes of patient and caregiver proxy reports on the PDQ-39 and used their correlations to provide additional information when a subset of the outcomes are missing. Finally, joint models for the longitudinal QOL scale outcomes and the time to drop out were fit to incorporate the drop out information. Even if the outcomes for one scale were not missing at random conditional on the observed data for the scale, measurements on other scales at the time, or time to drop out, could serve as proxies. Models for different outcomes and time to drop out were joined through shared covariance structures and/or shared random effects.

C.9.d.3. Plans to use validated methods to deal with missing data that properly account for statistical uncertainty due to missingness (MD-3)

The half rule for scales is a standard method for imputing missing items for a scale when the items are not hierarchical. The scales we used had already been validated, and when available, we uses scale-specific missing data imputation rules. Mixed models by their nature utilize within subject correlation data, even if some participants are missing data for some time points. Joint models connected through random effects is an established method for incorporating missingness including linkage of patient and caregiver proxy data as well as related scales within a participant.[57]

C.9.d.4. Recording and reporting all reasons for dropout and missing data, and account for all patients in reports (MD-4)

See Section **C.3** for inclusion and exclusion criteria. These criteria were chosen to maximize inclusion and generalizability while maintaining sound design and interpretability of results. We

tracked study participants from the time of initial contact and record screening failures (including reasons for screen failure), as well as dropouts. Dropout data included obtainable specific reasons for dropping out, who decided patient would dropout, and whether dropout involved all or only some activities. This data has been reported using standard CONSORT diagram showing flow of participants from initial contact through study completion.[65] Missing data are recorded and methods to handle missing data, including dropping forms, items, or subjects and/or use of imputation methods are reported for analyses in manuscripts as are rates of missingness.

C.9.d.5. Examine sensitivity of inferences to missing data methods and assumptions, and incorporate into interpretation (MD-5)

The covariances and random effects connecting the joint models were tested for statistical significance. The joint model method is sensitive to the structure of the model. Sensitivity analyses compared the results for the different approaches. All methods assumed the data is only missing at random. Missingness conditioned on available data is not dependent on values of the missing variables. Another possible sensitivity analysis is to compare with results from a complete case model, but patients with complete data may differ from patients with incomplete data while the complete case model assumes missing completely at random and will have a smaller sample size. The missing data methodology anticipates two patterns of missingness: intermittent missingness for some scales, but not others, and permanent loss to follow up. The half rule assumes the data for the missing items is missing completely at random conditional on the non-missing items in the scale, but the scales (or subscales) have been validated for consistency. As assumptions for missing data methods are usually difficult, if not impossible to verify, we will plan to include a summary of methods used and potential influences on data in the discussion section of any manuscripts generated from this study.

C.10. Changes to the Original Study Protocol: Since the submission of our original protocol we have made several changes (all discussed with PCORI and approved by IRB's when appropriate) as follows:

1) <u>Outcome Measures</u>: Per suggestions of PCORI Reviewers of our original proposal, through piloting our outcome measures with our patient and caregiver advisory group, and through

reflection by investigators on the purposes of our intervention we made the following changes to our outcome measures to improve responsiveness and patient-centeredness and reduce response burden. These changes were made prior to the collection of any data: a) Changed primary outcome from the PDQ-39 to the QOL-AD. This change reflects an intent to capture general QOL, domains important to overall QOL, and domains which are targeted by PC rather than the more restricted focus on health-related QOL and function captured in the PDQ-39; b) Change of symptom burden capture from the Memorial Symptom Assessment Scale (MSAS) to the shorter and PD-revision of the ESAS; c) Addition of Global Clinical Impression of Change to be assess both global impressions of patient and caregiver as well as to use for anchor-based methods for determining MCID for primary outcomes in this population; d) Addition of McGill QOL and PROMIS-29 scales to allow for fuller assessment of patient QOL and methodological development of QOL assessment in this population; and e) Elimination of clinic satisfaction survey (captured in more detail in qualitative interviews), neuropsychiatric inventory (NPI), and caregiver QOL to balance changes and additions with total length of assessment.

2) Inclusion Criteria: Due to both slow recruitment and theoretical considerations, inclusion criteria were expanded one year into study to allow inclusion of persons with other parkinsonian conditions, including progressive supranuclear palsy, dementia with Lewy bodies, multiple systems atrophy, and corticobasal degeneration. It was felt that this expansion of eligibility would improve generalizability and recruitment and that PD could still be analyzed as a subgroup in final analyses.
3) Intervention Protocol: Interim phone calls were made optional one year through the study due to feedback from patients, caregivers, and the clinical team that they were often not clinically productive and were often perceived as being a greater burden than benefit to participants.
4) Analysis: Linear trajectories over time were not seemingly a good fit. Instead time was treated as a categorical variable, and unrestricted time trajectories were fit, except for imposing a common baseline between the treatment groups. The repeated measures covariance was modeled with unstructured covariance matrices, with different matrices for

the different treatment groups. Satterthwaite degrees of freedom were used. The models allow for maximum flexibility based on the data, and compensate for missing data so long as a subject has some measurements included in the model and the data is not missing "not at random." The common baseline constraint was removed if there was evidence of a baseline treatment group differences. There was no evidence against the baseline assumption for most outcomes, except for QOL-AD (caregiver perspective) and UPDRS III.

The baseline covariates for the patient models were updated to include variables projected to influence QOL and caregiver burden: site, gender, age, disease duration, presence of caregiver, MOCA score (baseline), Hoehn and Yahr (baseline) (< 3 vs >= 3). Depression and UPDRS motor score were dropped from the covariates as we felt that depression could be an important effect modifier and that disease severity might be more accurately and simply captured through overall Hoehn and Yahr disease stage. In looking at patterns of missingness we found the following variables were significantly associated with missingness and were added as covariates to our adjusted model to reduce disruptions to the assumption of data missing at random: race (Caucasian vs Not Caucasian), Married (yes vs no), Education (less than college degree vs college degree or more).

The baseline covariates for caregivers included: site, caregiver gender, caregiver age, caregiver sharing the same residence as patient (yes vs no), duration of caregiving, patient MOCA score (baseline), patient Hoehn and Yahr (baseline) (< 3 vs >= 3), caregiver race (Caucasian vs Not Caucasian), caregiver married (yes vs no), caregiver education (less than college degree vs college degree or more).

The effect of the baseline covariates were allowed to vary with time point, but they were not effect modifiers for the differences between treatments groups unless specified. Race and marriage were included as covariates partly because of their association with discontinuation. Education was a proxy for socio-economic status, but with less missingness than income, and its categories were more collapsible.

For the primary outcomes, QOL-AD and ZBI, treatment effect modifiers were considered, including baseline values of the response. When baseline values were in the model, the outcome modeled was the change from baseline. Other variables tested for treatment effect

modifiers for QOL-AD (patient perspective), adjusted for covariates, included: non-standard PD diagnosis, presence of caregiver, study site, patient gender, patient age, NAT-PD (sum), ZBI at baseline (for patients with caregivers), MOCA score at baseline, HY scale at baseline, HY scale binary (<3 vs >= 3) at baseline, HADS anxiety and depression for patient at baseline, ESAS PD at baseline, PG-12 score at baseline. For ZBI the other tested treatment effect modifiers, adjusted for covariates, included: non-standard PD diagnosis, study site, caregiver gender, caregiver age, QOL-AD (patient perspective) at baseline, NAT-PD (sum), MOCA score at baseline, HY scale at baseline, HY scale binary (<3 vs >= 3) at baseline, HADS anxiety and depression for patient at baseline, ESAS PD at baseline, PG-12 score at baseline. NAT-PD was an ad-hoc created scale, found by summing the NAT-PD survey items. However, its Cronbach's alpha was only 0.73. The treatment effect modifiers were allowed to vary by time point. The study may not have adequate power to demonstrate heterogeneity of treatment effects at a statistically significant level.

There was initially some confusion about whether the QOL-AD proxy scale for the caregivers was meant to evaluate the caregiver or caregiver's perception of the patient. This resulted in a loss of baseline observations of caregiver proxy QOL-AD values for the UCSF site. There are only 155 usable responses to caregiver proxy QOL-AD at baseline (88.6% of patients with caregivers), compared to 171 for ZBI (97.7% of patients with caregivers), and 205 for patient reported QOL-AD (97.6% of patients). This was an issue when baseline conjoint QOL-AD as a covariate model and an effect modifier were used, because then it cost an entire subject. Attempted remedies included both jointly modeling QOL-AD for patients and caregivers and using multiple imputation to fill in the missing data. From the joint model results, composite scores were constructed from weighted combinations of the patient and caregiver perspective. One weighted patient:caregiver in a 2:1 ratio (as recommended in original publication but without statistical justification)[38], and another weighted them equally.

Missing covariates at baseline caused considerable data loss in the longitudinal regression models, so missing baseline covariates were filled in with multiple imputation in the sensitivity analyses.

Partly for computational reasons, joint longitudinal/time to discontinuation models, with shared random effects, were impractical to apply to multi time point categorical time models with unstructured repeated measures covariance matrices. Instead multiple imputation was used to fill in missing variables. Baseline covariates aside, missingness was mostly monotonic, with 27 discontinuations out of 210 patients (12.86%). Missing baseline values for covariates and outcomes were imputed in the fully conditional predictive mean matching models for continuous outcomes, and fully conditional logistic models for categorical variables. MCMC was used to fill in intermittent missingness among longitudinal responses, followed by monotonic predictive mean matching models. Imputation on the longitudinal outcomes was performed separately for the treatment groups. The imputation models included the same variables used in the longitudinal outcome models, along with whether a patient discontinued (excluded at later time points, there was insufficient sample to break down by reason), and previous values of a longitudinal outcome. Imputation models for ZBI included QOL-AD (patient perspective) as a variable, and imputation models for QOL-AD (caregiver perspective) included QOL-AD (patient perspective) and ZBI. Mean match models used the nearest five observations, fully conditional models used 200 burn in iterations, and 200 imputed data sets were created. Results for imputed data sets were combined and tested using standard methods. Calculations were performed in SAS 9.4, using proc mi and proc mianalyze. For proc mianalyze the effective degrees of freedom input used the denominator degrees of freedom from the un-imputed model results

Cross-overs patients, who for medical reasons were switched from controls to palliative care, were accounted for in a sensitivity analyses by assigning to them the same treatment effect corresponding to the amount of time since they started palliative treatment. For example, a decision might be made at the 3-month time point to switch a control patient to palliative care to relieve suffering. The first palliative care may then be administered at 6 months, time = 0 for palliative care treatment. At the 9-month visit the patient would be assigned the palliative care treatment effect for the 3-month visit, since palliative care has been in effect for the patient for three months.

D. Study Management across Sites

Laura Palmer will act as project coordinator across sites and will handle issues relating to budget, regulatory concerns, and conduct of the study in coordination with the PI, Dr. Kluger. This will include annual site visits to review study related procedures and documentation. All data will be entered into a central electronic HIPAA compliant database (REDCAP) housed and managed at the University of Colorado and maintained by our UCD biostatisticians Drs. Stefan Sillau and Diane Fairclough. Dr. Sillau will routinely check database for accuracy and completeness and notify local PIs and coordinators of any issues. Hard copies of all study related documents will be housed at local sites and kept in locked filing cabinets in locked research offices and kept for a minimum of five years following completion of the study. Ongoing and frequent coordination and communication between all three sites will occur with ad hoc, as well as regularly scheduled monthly calls and emails. This will allow for quick response to concerns if/as they arise during the study.

E. Potential Problems and Alternative Strategies

1) *As the intervention is enacted by three experienced teams at tertiary care centers will it be replicable or relevant for typical clinical practice?* We have taken several measures to ensure that our intervention is replicable across study sites and other centers. Our first planned manuscript will include our study protocol and a detailed description of our intervention and clinic related forms to allow dissemination to interested sites. Our study neurologists have not completed formal palliative training; rather the clinic structure itself is the key to providing the time and interdisciplinary skills needed to address patient and caregiver needs.
2) *What if the intervention is not successful in meeting primary or secondary outcomes?* This will be an important, if undesired, result that would still contribute to this field and suggest a need for alternate approaches. The value of Aim 3 will be heightened in this scenario by examining benefits to subgroups (suggesting a need for better screening) and eliciting patient and caregiver input on services and delivery.
3) *What if recruitment is slow or retention below expected?* Our recruitment goals are modest

and similar goals have been achieved by investigators at all study sites. If needed, further recruitment efforts may include adding additional local sites through community partners and/or more aggressive advertising strategies. A high drop-out rate would also be an important data point suggesting a need to revise delivery method.

4) *This population is heterogenous and it is possible that outcomes may not apply to all subgroups or that important outcomes for particular subgroups are missed.* This is a significant challenge facing palliative care for PD which may be one factor hampering palliative research for this disease versus more homogenous illnesses such as amyotrophic lateral sclerosis and Alzheimer's disease.[17] As this heterogeneity is a naturally occurring feature of PD we have made every effort to include the full spectrum of PD in our screening forms and intervention. Aim 3 will provide subgroup analyses to inform future interventions and screening efforts.
5) *Will results be biased without blinding?* It is impossible to truly blind an intervention of this nature. We have used patient-reported outcomes to increase the relevance of results as there are no objective biomarkers relevant to our research questions. Despite this, prior multidisciplinary and palliative intervention trials have reported negative results suggesting that blinding biases do not overwhelm clinical effects.[66]

F. Future Directions

If this intervention is successful in improving patient QOL and/or caregiver distress we would plan to explore alternate delivery methods to improve the efficiency and dissemination of this approach including telemedicine, group visits, smaller teams and mixed approaches (e.g. in person physician visit and telehealth counseling). Cost-effectiveness studies would also be an important follow-up question to influence system level decisions for this model of care. Lastly, application to other progressive neurological illnesses, including mixed populations, may be warranted as patients with advanced chronic neurological illness tend to be similar in terms of symptoms and needs.

If this intervention is not successful, future studies would be guided by the results of Aim 3. Benefits found in specific subgroups would suggest a need for the development of better

screening instruments, while poor responses across the sample would suggest a need for a different model. High drop-out rates or noncompliance with the intervention would suggest a need for alternate delivery methods.

G. Human Subjects

G.1. Risks to Human Subjects

1.a. Human Subjects Involvement, Characteristics and Design

Due to the nature of our research questions regarding clinical, psychosocial, and spiritual needs in PD patients and their family caregivers we feel that involvement of human research subjects is necessary and justified. As we will discuss below, the risks of this research to human subjects are minimal and the potential for benefit for the involved human subjects, other people and family members living with PD, and society is significant.

We will recruit PD patients over age 40 diagnosed with probable PD using UK Brain Bank Criteria[30] or atypical parkinsonian disorders (APD) who are identified to have high potential for palliative care needs using the Palliative Care Needs Assessment Tool.[28] When present their family caregivers will also be included. We plan to recruit a total of 210 PD patients and project that at least 80% will have family caregivers who will also be included in this study.

We will *select* participants using the following inclusion and exclusion criteria described above. PD patients with dementia (PDD) may be considered a vulnerable population but are necessary to answering our research questions. For all patients with cognitive impairment we will formally assess capacity to consent using the University of California Brief Assessment of Capacity to Consent (UBACC) modified for our specific study design where needed.[67] For patients who fail this screen or if investigators have additional concerns we will require that their guardian participate in the consenting process and sign an informed consent. We will also require that PDD subjects give consent, or at minimum assent if fully informed consent is not possible. Our protocol and consent forms will be approved by our local IRBs before approaching any potential subjects.

Caregivers will be consented and enrolled using a caregiver-specific consent form.

After enrollment, screening, and consent, patients will be randomized in a 1:1 ratio to either standard care or standard care supplemented by an outpatient interdisciplinary clinic. The interdisciplinary team will consist of a neurologist with palliative care experience, a nurse, social worker, and a chaplain. For the standard care arm, patients not currently seeing a neurologist randomized to this arm have appointments with a neurologist covered by their insurance or patient assistance programs arranged by the study team. Patients will be asked to see their neurologist at least every three months to match the frequency of visits in the intervention arm and may see their PCP at their typical frequency. In the intervention arm, palliative care visits will be performed in person (or telemedicine if needed for rural areas and/or limited transportation or health issues) every three months. In addition, visits will be supplemented by phone calls one week after each visit to confirm understanding of team recommendations and at six weeks to check-in with patient and caregiver. Patients and caregivers may also contact the team as needed. Summaries of visits will be sent to the patient, caregiver, PCP, and neurologist and suggestions for care outside of palliative care issues will be left to the patient's Standard Care team.

G.1.b. Sources of Materials

Data will be obtained from subjects non-invasively via interviews, questionnaires, cognitive testing, and neurological examination as described above.

This data will be accessible only to the research team and, if requested, by appropriate regulatory bodies such as the IRB and FDA. All documents generated by this study, including consent, will be stored in a locked filing cabinet in the locked office of the Principal Investigator (PI). Electronic data will be de-identified with the exception of a correlational spreadsheet including demographic information and study IDs for scheduling and safety contact purposes. This, and all other data, will be stored on a password protected network drive and backed-up on the hard-drive of the PI, also password protected, and kept in the PI's locked office. Identifying information will not be shared outside of the research team or regulatory bodies (e.g. IRB).

G.1.c. Potential Risks

There are minimal risks involved in the proposed research. There are no known significant risks from the intervention, interviews, or surveys proposed for this study. Experiences from collaborators on this study suggest that most patients and caregivers welcome the opportunity to talk about their experiences, even on these potentially delicate subjects. Our interviewers will be cognizant of signs of emotional distress in participants and will suggest breaks and remind them that participation is voluntary. Cognitive testing and surveys as proposed in this study may induce boredom or restlessness. Any new diagnoses detected during screening (e.g. depression or dementia) will be referred for appropriate treatment, including emergent treatment if indicated. There are no known significant risks to the proposed team-based intervention for addressing palliative care needs. Participants will be reminded of the voluntary nature of this research and informed that we can follow them outside of the intervention or they can completely discontinue the study if they find it excessively burdensome or otherwise troubling.

G.2. Adequacy of Protection against Risks

G.2.a. Recruitment and Informed Consent

Participants will be recruited from the academic medical centers (University of Colorado, University of California San Francisco, University of Alberta), associated clinics, community neurologists, and direct advertisements and contact with patients (e.g. clinicaltrials.gov, community seminars). Protocols will be approved by the relevant regulatory body at each institution before any contact is made with potential participants. We will obtain informed consent before any study related activities are performed. Potential subjects will be specifically told that their participation or lack of participation will not affect the clinical care they receive. For PD Dementia subjects, special procedures will be in place to involve both guardians and patients in the informed consent process, and both guardians and subjects will be required to give consent in person or by phone if performing visit by telehealth (or assent for PDD subjects unable to give full informed consent). The consenting process will use standard IRB terminology and will include a full description of expected subject involvement, procedures, risks, rights, and benefits. Capacity will be formally assessed in any patients with cognitive impairments and guardian consent and patient assent will be required in these cases.[67]

G.2.b. Protections Against Risks

All subjects will be reminded that they can take breaks or discontinue the study at any time if they find it upsetting. PD dementia subjects will be required to give consent (if they have capacity) or assent with caregiver consent to avoid potential manipulation of a vulnerable population. Regarding patient privacy, all research procedures will be performed in a confidential setting. All documents generated by this study, including consent, will be stored in a locked filing cabinet in the locked office of the PI. Electronic data will be de-identified with the exception of a correlational spreadsheet including demographic information and study IDs for scheduling and safety contact purposes. This, and all other data, will be stored on a password protected network drive and backed-up on the hard-drive of the PI, also password protected, and kept in the PI's locked office. Identifying information will not be shared outside of the research team or regulatory bodies (e.g. IRB).

G.3. Potential Benefits of the Proposed Research to Human Subjects and Others

All subjects participating will receive a free neurological examination and history from a movement disorder trained specialist and screening for dementia and depression. These results will be reviewed by a study neurologist and referred for appropriate care if abnormalities are identified. Participants randomized to the standard care arm will have care with a neurologist arranged if they do not already have this in place. Participants randomized to the palliative intervention will have access and care from an interdisciplinary team. This proposal has the potential to further our knowledge of care models in PD and has the potential to influence our ability to address many currently unmet needs for patients and caregivers. As discussed below, the knowledge gained by this proposal has a significant potential to advance our knowledge of palliative needs in PD which has large positive implications for PD patients, the scientific community, and society.

G.4. Importance of Knowledge to be Gained

Parkinson's disease (PD) affects 1-2% of adults over age 65 representing approximately 1.5 million Americans. Although PD is traditionally characterized by motor symptoms such as tremor and slowed movement more recent research shows that PD patients also commonly experience non-motor symptoms such as pain, fatigue, depression and dementia. PD interferes

with patients' quality of life, leads to disability, decreases length of life, and causes significant distress for caregivers. Unfortunately, many of the needs most important to patients and their caregivers (e.g. depression, planning for the future) are poorly addressed under current models of care. The goal of this proposal is to take a patient-centered approach to answering these questions. Our specific objectives are to: 1) Determine whether an outpatient palliative care team improves patient quality of life and caregiver distress compared to standard care with a neurologist; 2) Determine what patient and caregiver characteristics best predict benefit from palliative care services; and 3) Interview patients and caregivers to optimize service delivery and selection. This project is important to patients and their family caregivers because it assesses a new approach to PD care that has the potential to improve quality of life, caregiver burden and other important outcomes (e.g. nursing home placement). By testing the effectiveness of this approach and determining who benefits most we hope to provide a new option to assist those PD patients at highest risk for poor outcomes. Information generated by this proposal will also be important for other stakeholders interested in PD including hospitals, insurers, and patient advocacy organizations.

G.5. Data and Safety Monitoring Plan

We will not assemble a Data and Safety Monitoring Board (DSMB) as this is a minimal risk study comparing the effectiveness of two currently available approaches to the care of PD and APD patients. The site PI will be notified of any adverse events occurring at all sites and will notify the study's central IRB (COMIRB) of all adverse events and any change in our appraisal of the risks and benefits of this study from our data or other published literature.

G.6. ClinicalTrials.gov Requirements

We will register this trial with clinicaltrials.gov prior to enrollment of the first participant at any site.

H. References

1. Dorsey ER, Constantinescu R, Thompson JP, et al. Projected number of people with Parkinson disease in the most populous nations, 2005 through 2030. *Neurology.* 2007;68(5):384-386.

2. Kowal SL, Dall TM, Chakrabarti R, Storm MV, Jain A. The current and projected economic burden of Parkinson's disease in the United States. *Mov Disord.* 2013;28(3):311-318.
3. Bernal-Pacheco O, Limotai N, Go CL, Fernandez HH. Nonmotor manifestations in Parkinson disease. *Neurologist.* 2012;18(1):1-16.
4. Hely MA, Reid WG, Adena MA, Halliday GM, Morris JG. The Sydney multicenter study of Parkinson's disease: the inevitability of dementia at 20 years. *Mov Disord.* 2008;23(6):837-844.
5. Beyer MK, Herlofson K, Arsland D, Larsen JP. Causes of death in a community-based study of Parkinson's disease. *Acta neurologica Scandinavica.* 2001;103(1):7-11.
6. Murphy S XJ, Kochanek K. Deaths: Preliminary Data for 2010. *National Vital Statistics Reports.* 2012;60(4):1-52.
7. Vossius C, Nilsen OB, Larsen JP. Parkinson's disease and nursing home placement: the economic impact of the need for care. *Eur J Neurol.* 2009;16(2):194-200.
8. Snell K, Pennington S, Lee M, Walker R. The place of death in Parkinson's disease. *Age Ageing.* 2009;38(5):617-619.
9. Martinez-Martin P, Arroyo S, Rojo-Abuin JM, Rodriguez-Blazquez C, Frades B, de Pedro Cuesta J. Burden, perceived health status, and mood among caregivers of Parkinson's disease patients. *Mov Disord.* 2008;23(12):1673-1680.
10. Nielsen M, Hansen J, Ritz B, et al. Cause-specific mortality among spouses of Parkinson disease patients. *Epidemiology.* 2014;25(2):225-232.
11. Lanoix M. Palliative care and Parkinson's disease: managing the chronic-palliative interface. *Chronic Illn.* 2009;5(1):46-55.
12. Hu M, Cooper J, Beamish R, et al. How well do we recognise non-motor symptoms in a British Parkinson's disease population? *J Neurol.* 2011;258(8):1513-1517.
13. Bunting-Perry LK. Palliative care in Parkinson's disease: implications for neuroscience nursing. *J Neurosci Nurs.* 2006;38(2):106-113.
14. Howlett JG. Palliative care in heart failure: addressing the largest care gap. *Curr Opin Cardiol.* 2011;26(2):144-148.
15. Heigener DF, Rabe KF. Palliative care concepts in respiratory disease. *Respiration.* 2011;82(6):483-491.
16. Richfield EW, Jones EJ, Alty JE. Palliative care for Parkinson's disease: a summary of the evidence and future directions. *Palliat Med.* 2013;27(9):805-810.
17. Boersma I, Miyasaki J, Kutner J, Kluger B. Palliative care and neurology: time for a paradigm shift. *Neurology.* 2014;83(6):561-567.
18. Kluger BM, Fox S, Timmons S, et al. Palliative care and Parkinson's disease: Meeting summary and recommendations for clinical research. *Parkinsonism Relat Disord.* 2017;37:19-26.
19. Miyasaki JM, Long J, Mancini D, et al. Palliative care for advanced Parkinson disease: an interdisciplinary clinic and new scale, the ESAS-PD. *Parkinsonism & related disorders.* 2012;18 Suppl 3:S6-9.
20. Miyasaki J, Kluger B. Palliative Care for Parkinson’s Disease: Has the time come? *Curr Neurol Neurosci Rep.* In Press.
21. Heath SL, Lanier EM, Glass GA, Talmadge C, Woodward E, Galifianakis NB. Development of a palliative care clinic for advanced Parkinson's disease patients and their caregivers. *Movement Disorders.* 2012;27(Suppl 1):S563.

22. Kluger BM, Shattuck J, Berk J, et al. Defining Palliative Care Needs in Parkinson's Disease. *Mov Disord Clin Pract.* 2019;6(2):125-131.
23. Boersma I, Jones J, Coughlan C, et al. Palliative Care and Parkinson's Disease: Caregiver Perspectives. *J Palliat Med.* 2017.
24. Boersma I, Jones J, Carter J, et al. Parkinson disease patients' perspectives on palliative care needs: What are they telling us? *Neurol Clin Pract.* 2016;6(3):209-219.
25. Lim TT, Kluger BM, Rodriguez RL, et al. Rasagiline for the symptomatic treatment of fatigue in Parkinson's disease: A 3-center, placebo-controlled, pilot study (the REST trial). *Mov Disord.* 2013;28(Suppl 1):461.
26. Kluger BM, Rakowski D, Christian M, et al. Randomized, Controlled Trial of Acupuncture for Fatigue in Parkinson's Disease. *Mov Disord.* 2016;31(7):1027-1032.
27. Thorgrimsen L, Selwood A, Spector A, et al. Whose quality of life is it anyway? The validity and reliability of the Quality of Life-Alzheimer's Disease (QoL-AD) scale. *Alzheimer Dis Assoc Disord.* 2003;17(4):201-208.
28. Waller A, Girgis A, Currow D, Lecathelinais C. Development of the palliative care needs assessment tool (PC-NAT) for use by multi-disciplinary health professionals. *Palliat Med.* 2008;22(8):956-964.
29. Miyasaki JM, Long J, Mancini D, et al. Palliative care for advanced Parkinson disease: An interdisciplinary clinic and new scale, the ESAS-PD. *Parkinsonism Relat Disord.* 2012.
30. Hughes AJ, Daniel SE, Kilford L, Lees AJ. Accuracy of clinical diagnosis of idiopathic Parkinson's disease: a clinico-pathological study of 100 cases. *J Neurol Neurosurg Psychiatry.* 1992;55(3):181-184.
31. Holden SK, Jones WE, Baker KA, Boersma IM, Kluger BM. Outcome measures for Parkinson's disease dementia: a systematic review. *Mov Disord Clin Pract.* 2016;3(1):9-18.
32. Emre M, Aarsland D, Brown R, et al. Clinical diagnostic criteria for dementia associated with Parkinson's disease. *Mov Disord.* 2007;22(12):1689-1707; quiz 1837.
33. Willis AW, Schootman M, Evanoff BA, Perlmutter JS, Racette BA. Neurologist care in Parkinson disease: a utilization, outcomes, and survival study. *Neurology.* 2011;77(9):851-857.
34. Brown GC, Brown MM, Sharma S. Value-based medicine: evidence-based medicine and beyond. *Ocul Immunol Inflamm.* 2003;11(3):157-170.
35. Frank L, Basch E, Selby JV. The PCORI perspective on patient-centered outcomes research. *Jama.* 2014;312(15):1513-1514.
36. Zarit SH, Todd PA, Zarit JM. Subjective burden of husbands and wives as caregivers: a longitudinal study. *Gerontologist.* 1986;26(3):260-266.
37. Adelman RD, Tmanova LL, Delgado D, Dion S, Lachs MS. Caregiver burden: a clinical review. *Jama.* 2014;311(10):1052-1060.
38. Caramelli P, Laks J, Palmini AL, et al. Effects of galantamine and galantamine combined with nimodipine on cognitive speed and quality of life in mixed dementia: a 24-week, randomized, placebo-controlled exploratory trial (the REMIX study). *Arq Neuropsiquiatr.* 2014;72(6):411-417.
39. Logsdon RG, Gibbons LE, McCurry SM, Teri L. Assessing quality of life in older adults with cognitive impairment. *Psychosom Med.* 2002;64(3):510-519.

40. Larsson V, Engedal K, Aarsland D, Wattmo C, Minthon L, Londos E. Quality of life and the effect of memantine in dementia with lewy bodies and Parkinson's disease dementia. *Dement Geriatr Cogn Disord.* 2011;32(4):227-234.
41. Boersma I, Jones J, Coughlan C, et al. Palliative Care and Parkinson's Disease: Caregiver Perspectives. *J Palliat Med.* 2017;20(9):930-938.
42. Hoe J, Hancock G, Livingston G, Woods B, Challis D, Orrell M. Changes in the quality of life of people with dementia living in care homes. *Alzheimer Dis Assoc Disord.* 2009;23(3):285-290.
43. Zarit SH, Reever KE, Bach-Peterson J. Relatives of the impaired elderly: correlates of feelings of burden. *Gerontologist.* 1980;20(6):649-655.
44. Hebert R, Bravo G, Preville M. Reliability, validity and reference values of the Zarit Burden Interview for assessing informal caregivers of community-dwelling older persons with dementia. *Can J Aging.* 2000;19(4):494-507.
45. Higginson IJ, McCrone P, Hart SR, Burman R, Silber E, Edmonds PM. Is short-term palliative care cost-effective in multiple sclerosis? A randomized phase II trial. *J Pain Symptom Manage.* 2009;38(6):816-826.
46. Dahlin CM, Kelley JM, Jackson VA, Temel JS. Early palliative care for lung cancer: improving quality of life and increasing survival. *Int J Palliat Nurs.* 2010;16(9):420-423.
47. Thorgrimsen L, Selwood A, Spector A, et al. Whose quality of life is it anyway? The validity and reliability of the Quality of Life-Alzheimer's Disease (QoL-AD) scale. *Alzheimer Dis Assoc Disord.* 2003;17(4):201-208.
48. Miyasaki JM, Long J, Mancini D, et al. Palliative care for advanced Parkinson disease: an interdisciplinary clinic and new scale, the ESAS-PD. *Parkinsonism Relat Disord.* 2012;18 Suppl 3:S6-9.
49. Zigmond AS, Snaith RP. The hospital anxiety and depression scale. *Acta Psychiatr Scand.* 1983;67(6):361-370.
50. Peto V, Jenkinson C, Fitzpatrick R, Greenhall R. The development and validation of a short measure of functioning and well being for individuals with Parkinson's disease. *Qual Life Res.* 1995;4(3):241-248.
51. Peterman AH, Fitchett G, Brady MJ, Hernandez L, Cella D. Measuring spiritual well-being in people with cancer: the functional assessment of chronic illness therapy--Spiritual Well-being Scale (FACIT-Sp). *Ann Behav Med.* 2002;24(1):49-58.
52. Prigerson HG, Horowitz MJ, Jacobs SC, et al. Prolonged grief disorder: Psychometric validation of criteria proposed for DSM-V and ICD-11. *PLoS medicine.* 2009;6(8):e1000121.
53. S Fahn MotUDC. *Unified Parkinson's Disease Rating Scale.* Vol 2. New York: Macmillan; 1987.
54. Peto V, Jenkinson C, Fitzpatrick R. Determining minimally important differences for the PDQ-39 Parkinson's disease questionnaire. *Age Ageing.* 2001;30(4):299-302.
55. Klotsche J, Reese JP, Winter Y, et al. Trajectory classes of decline in health-related quality of life in Parkinson's disease: a pilot study. *Value Health.* 2011;14(2):329-338.
56. van der Marck MA, Bloem BR, Borm GF, Overeem S, Munneke M, Guttman M. Effectiveness of multidisciplinary care for Parkinson's disease: a randomized, controlled trial. *Mov Disord.* 2013;28(5):605-611.
57. Fairclough DL. *Design and analysis of quality of life studies in clinical trials.* 2nd ed. Boca Raton, Fl: Tarlor and Francis Group, LLC; 2010.

58. Bjornestad A, Pedersen KF, Tysnes OB, Alves G. Clinical milestones in Parkinson's disease: A 7-year population-based incident cohort study. *Parkinsonism Relat Disord.* 2017;42:28-33.
59. Bedard M, Molloy DW, Squire L, Dubois S, Lever JA, O'Donnell M. The Zarit Burden Interview: a new short version and screening version. *Gerontologist.* 2001;41(5):652-657.
60. Charlson ME, Pompei P, Ales KL, MacKenzie CR. A new method of classifying prognostic comorbidity in longitudinal studies: development and validation. *J Chronic Dis.* 1987;40(5):373-383.
61. Dalrymple-Alford JC, MacAskill MR, Nakas CT, et al. The MoCA: well-suited screen for cognitive impairment in Parkinson disease. *Neurology.* 2010;75(19):1717-1725.
62. Crabtree BF MW. *Doing Qualitative Research.* 2 ed. Thousand Oaks, CA: Sage Publications; 1999.
63. Bekelman DB, Nowels CT, Retrum JH, et al. Giving voice to patients' and family caregivers' needs in chronic heart failure: implications for palliative care programs. *J Palliat Med.* 2011;14(12):1317-1324.
64. Jones J, McDermott CM, Nowels CT, Matlock DD, Bekelman DB. The experience of fatigue as a distressing symptom of heart failure. *Heart Lung.* 2012;41(5):484-491.
65. Schulz KF, Altman DG, Moher D. CONSORT 2010 statement: updated guidelines for reporting parallel group randomised trials. *Bmj.* 2010;340:c332.
66. van der Marck MA, Munneke M, Mulleners W, et al. Integrated multidisciplinary care in Parkinson's disease: a non-randomised, controlled trial (IMPACT). *Lancet Neurol.* 2013;12(10):947-956.
67. Jeste DV, Palmer BW, Appelbaum PS, et al. A new brief instrument for assessing decisional capacity for clinical research. *Arch Gen Psychiatry.* 2007;64(8):966-974.

I. Acknowledgments

We would like to acknowledge our patient and caregiver advisory council who were instrumental in all aspects of the study and are continuing to contribute to our ongoing publications. We would specifically like to thank Kirk Hall who has been a leader for many in the Parkinson’s community through his support group, blog, books, manuscripts, talks and advocacy to confront challenging issues head on and specifically to acknowledge the importance and potential of palliative care to help persons living with Parkinson’s disease and related disorders. We would like to thank the Parkinson Foundation (PF) for their ongoing support of this work, including sponsoring two international Parkinson’s disease and Palliative Care working groups, and we look forward to working with them more in the future to implement this model of care more broadly. Finally, we would like to acknowledge Laura Palmer who was an outstanding project coordinator. This study would not have been complete without her diligence, attention to detail and commitment to this work.

APPENDIX 1: Palliative Care Needs Assessment Tool – Parkinson's Disease

STUDY ID#: ______________________________ **DATE:** ________________

Section 1a: Red Flags – If present, be alert for unmet palliative care need:	**Y**	**N**
Red flag symptoms? (Persistent hallucinations / ≥2 Falls / Aspiration / Hoehn and Yahr 3 -see guidance for details)		
Admission to residential or nursing care?		
Section 1b: Priority referral for further assessment:	Y	N
No carer?		

**Section 2: PATIENT WELLBEING** ***("Does the patient have……")***	**Level of Concern**		
Who provided this information?<br>Patient ☐ Carer ☐ Both☐	None	Some/ Potential	Significant
Unresolved physical symptoms? (Motor / Non-motor)			
Unresolved psychological or neuropsychiatric symptoms?			
Problems with daily living activities?			
Spiritual or existential concerns?			
Financial or legal concerns?			
Health beliefs, cultural or social factors making care delivery complex?			
**Information needs**: Prognosis ☐ Diagnosis ☐ Treatment options ☐<br>Financial/legal issues ☐ Support services ☐ Social/emotional issues ☐			

**Section 3: ABILITY OF CARER OR FAMILY TO CARE FOR PATIENT** ***("Is the Carer / Family…….")***			
	**Level of Concern**		
Who provided this information?<br>Patient ☐ Carer ☐ Both☐	None	Some/ Potential	Significant
Distressed about the patient's symptoms? (Motor / Non-motor / neuro-psychiatric)			
Having difficulty providing physical care?			
Having difficulty coping?			
Concerned about financial or legal issues?			
Experiencing problems that are interfering with inter-personal relationships or functioning?			
**Information needs**: Prognosis ☐ The diagnosis ☐ Treatment options ☐<br>Financial/legal issues ☐ Support services ☐ Social/emotional issues ☐			

**Section 4: CARER/FAMILY WELLBEING "Carer or family experiencing……."**	**Level of Concern**		
	None	Some/ Potential	Significant
Problems that are interfering with their wellbeing or functioning?			
Grief over the impending or recent death of the patient?			

Comments:

ISSUES TO CONSIDER WHEN RATING LEVEL OF CONCERN

RED FLAGS – If present consider further assessment by own team +/- SPCS if required

Visual hallucinations –"yes" if hallucinations are formed and persistent. Do not include if associated with inter-current illness or medication change.
Recurrent falls –"yes" if multiple (>2) falls have occurred. Single, isolated falls, even in the context of injury, should not be included here.
Pneumonia / choking – May indicate aspiration and should trigger further palliative review in addition to SLT where appropriate.
Hoehn and Yahr stage 3 (Bilateral disease, mild to moderate disability and impaired postural reflexes) is associated with reduced quality of life.
24 hour care – admission to either residential or nursing care should trigger exploration of further supportive and palliative care needs.
Failure to attend clinic – may indicate physical or social difficulties, change in circumstance or acute admission preventing attendance.

PATIENT WELLBEING

Physical symptoms
• Fatigue, drowsiness, Pain, Constipation, Poor sleep, Urinary urgency, frequency, or incontinence, swallowing difficulty, SOB, Drooling, Spasms.
Activities of daily living
• Is the patient having difficulty with toileting, showering, bathing, or food preparation?
• Do they require more information to maximise their daily function –see below
Neuro-psychiatric / Psychological - "Does the patient have…."
• Thinking or memory problems, which interfere with wellbeing / relationships?
• Hallucination or behavioural issues which require assistance or evidence of psychosis?
• Sustained lowering of mood, tearfulness or guilt? Loss of pleasure in usual activities? Feelings of anxiety, apprehension, anger or fearfulness?
• Is the patient struggling with the implications of, or emotional response to the diagnosis?
• Is the patient requesting a hastened death?
Spiritual/Existential – "Is the patient…."
• Feeling isolated or hopeless?
• Feeling that life has no meaning or that his/her life has been wasted?
• Having difficulty thinking about the future?
• Requiring assistance in finding appropriate spiritual resources or services?
Financial/Legal concerns
• Consider loss of income, costs of treatment, travel expenses, equipment, or future care needs (such as residential care)?
• Is the family socio-economically disadvantaged?
• Is the patient or family aware of the various financial schemes available and do they need assistance in accessing these (e.g. social worker)?
• Are there conflicting opinions between patient and family relating to legal issues such as end-of-life care options and advance care plans?
Health Beliefs, Social and Cultural – "Does the patient or family…"
• Have beliefs or attitudes that make health care provision difficult – for example believing that palliative / hospice care is not available to them?
• Have communication difficulties – consider language and disease related issues (hypophonia / freezing of speech)?
• Feel socially isolated? If so are they avoiding peer support groups due to concerns such as "downward comparison"?
• Need information passed on to a particular member of the family or cultural group?
• Want information about prognosis to be withheld from the patient, or are they reluctant to discuss prognosis? If so, has this been explored?
• Have logistical difficulties accessing services (distance, transport, cost)?
Information – "Is the patient aware of/that…."
• Available services and do they need assistance accessing these? (e.g. financial /legal assistance, psychological services, support groups, pastoral care.)
• Advance Care Planning (ACP) and have their views / attitudes towards it been explored?
• PD is progressive, incurable and shortens life?
• Does the patient want more information about the course and prognosis of the disease and treatment options?

ABILITY OF CARER OR FAMILY TO CARE FOR PATIENT

Physical symptoms
• Are the patient's physical symptoms causing the carer and/or family distress?
Providing physical care
• Is the carer having difficulty coping with activities of daily living, medical regimes or practical issues such as equipment and transport?
• Have they received all the practical information they require?
Neuro-psychiatric / Psychological – "Is the carer / family…"
• Having difficulty coping with the patient's memory problems, hallucinations or behavioural issues?
• Having difficulty coping with the patient's psychological symptoms (esp. anxiety and depression)?
• Requesting a hastened death for the patient?
Family and Relationships
• Is there any communication breakdown or conflict between the patient and family over prognosis, treatment options or care giving roles?
• Is the patient particularly concerned about the impact of the illness on the carer or family?
• Is the disease having an adverse effect on the relationship between patient and carer? (May wish to consider impact of physical, psychological and personal cares, sexual dysfunction and change to previous roles within relationship.)
Information – "Do/are the carer or family…"
• Require more information about the course and prognosis of the disease and treatment?
• Aware of available services/ need assistance accessing these? (e.g. Financial/legal psychological services, support groups, pastoral care.)

• Are the information needs of the patient and family congruous?

**CARER AND FAMILY WELLBEING - “Do the carer or family…”**
**Physical and psychosocial**<br>• Experience physical strain, ill health, fatigue, disturbed sleep? Is there evidence of anxiety, depression or feelings of hopelessness?<br>• Have spiritual/existential issues that are of concern?<br>• Currently feel that caring has a net positive or negative affect for them personally and their relationship with the patient?<br>**Grief (pre and post death)**<br>• Experience intrusive images, severe emotion, denial of implications of loss to self and neglect of necessary adaptive activities at home or work?<br>•Know of the progressive nature of PD? Has future care planning been considered? (If patient not capacitous this may include best interest decisions)<br>• Have access to support services (Such as PD Nurse Specialist, SPC, Local support groups, post bereavement support?)

Appendix 2: Checklists

NEUROLOGIST CHECKLIST – Initial Visit v1.0 11/11/15

REDCap ID/Study ID: ____________ / ___________ **Date: ________________**

- ☐ Complete Medical History
  - ☐ History of Parkinson's disease
    - Recent hospitalizations or other complications
  - ☐ Patient and Caregiver Goals of Care
    - How define quality of life
    - Worries about the future/Fears
    - Sources of support and meaning
    - If caregiver present – any issues of safety, overwhelmed, burnt-out, own health concerns
    - Educational needs
    - Status of Advance Care Plans/Documentation
    - Specific Goals of Care
  - ☐ Review of Systems
    - Psychiatric Symptoms (Anxiety and Depression)
    - Behavioral Issues and Psychosis
    - Edmonton Symptoms Assessment Scale PD
    - Falls, balance and mobility
    - Swallowing, sialorrhea and nutrition (weight changes)
    - Other Symptoms
  - ☐ Review Medications
  - ☐ Allergies and medication side effects
  - ☐ Past Medical and Surgical History
  - ☐ Social History
    - Current Living Situation
  - ☐ Family History
- ☐ Physical Examination
  - ☐ Review orthostatic vital signs and weight
  - ☐ Motor exam (UPDRS)
- ☐ Assessment
  - ☐ Review Prognosis and Disease Stage
  - ☐ Reflect Goals
- ☐ Plan
  - ☐ Safety issues (e.g. home safety, abuse, driving)
  - ☐ Medication changes
  - ☐ Referrals for other services
  - ☐ Heads up to other team members
  - Need for Palliative Care Referral (in clinic or outside)
    - Complex symptom management (e.g. pain)
    - ☐ Follow-up plan including issues for phone call

Signature: ______________________________ **Date: ___________________**

NEUROLOGIST CHECKLIST – Return Visit

v1.0 11/11/15

REDCap ID/Study ID: __________ / __________ Date: ______________

- ☐ Interval Medical History
  - Any interval events (e.g. UTI)
  - Changes in functional status
  - Patient and Caregiver Goals of Care
    - Any changes/updates needed to goals
    - Any new issues from caregiver perspective
  - Review of Systems
    - Psychiatric Symptoms (Anxiety and Depression)
    - Behavioral Issues and Psychosis
    - Edmonton Symptoms Assessment Scale PD
    - Falls, balance and mobility
    - Swallowing, sialorrhea and nutrition
    - Other Symptoms
  - Review Medications
  - Social History
    - Changes in living situation
- ☐ Physical Examination
  - Any change in weight; repeat orthostatic vital signs if relevant
  - Motor exam (UPDRS)
- ☐ Assessment
  - Review Prognosis and Disease Stage
  - Reflect Goals
- ☐ Plan
  - Safety issues (e.g. home safety, abuse, driving)
  - Medication changes
  - Referrals for other services
  - Heads up to other team members
  - Need for Palliative Care Referral (in clinic or outside)
    - Complex symptom management (e.g. pain)
  - Follow-up plan including issues for phone call

Signature: ______________________________ **Date:** ______________

SOCIAL WORKER CHECKLIST: Initial Visit

v1.0 11/11/15

REDCap ID/Study ID: ___________ / __________ **Date: _____________**

☐ **SUPPORT SITUATION FOR PATIENT:**

- ☐ Partner
- ☐ Family
- ☐ Friends
- ☐ Neighbors

☐ **ASSESSMENT OF CURRENT LIVING ENVIRONMENT:**

Independent

- ☐ Address safety in home - Physical, emotional and sexual abuse
- ☐ System to prepare medications, Dr. appointments
- ☐ Adaptive equipment
- ☐ Assistance at home: Home health, R.N., C.N.A., P.T., O.T.
- ☐ Has there been a home safety evaluation
- ☐ Non-medical Care
- ☐ Transportation
- ☐ Mental Health Support

☐ **LONG TERM CARE NEEDS:**

- ☐ Assisted Living/Skilled Nursing
- ☐ Medication plan at facility
- ☐ Response time: Are needs being met
- ☐ Transportation, adaptive equipment
- ☐ Hospice

☐ **CAREGIVER DISTRESS:**

- ☐ Assessment of Partner's physical and emotional health,(abuse by P.W.P)
- ☐ Resources in the community for respite care, Adult Day Care Programs
- ☐ Support group for Caregiver
- ☐ Mental health support system, i.e. Psychologist, counselor
- ☐ Back up plan to care for P.W.P. if unable to.

☐ **FINANCIAL ISSUES AND CONCERNS:**

- ☐ Employed currently - Assess if have short term - long term insurance
- ☐ Health Insurance coverage
- ☐ Medicare
- ☐ Medicaid eligibility
- ☐ Qualifications for Social Security Disability Income or Supplemental Security Income

☐ PLAN:

Referrals for resources in the community:

- ☐ Elder law attorney for Power of Attorney, Guardianship, Conservatorship, finances, Disability Denials
- ☐ Funding assistance for medications, medical equipment
- ☐ Support groups
- ☐ Local Parkinson Association
- ☐ Counselors for chronic illness
- ☐ If needed, local county Adult Protection
- ☐ Provide educational forums on Parkinson's in the community
- ☐ Web sites: Michael J. Fox, Davis Phiney Foundation and National Parkinson Association

☐ **Follow up phone call to address further needs of patient and family.**

Signature: ____________________________ **Date:** __________________

SOCIAL WORK CHECKLIST: Return Visit

v1.0 11/11/15

REDCap ID/Study ID: ___________/ __________ Date: ________________

- ☐ **REVIEW OF SUPPORT SYSTEM CHANGES:**
  - ☐ Partner
  - ☐ Family
  - ☐ Friends
  - ☐ Neighbors

- ☐ **CURRENT LIVING SITUATION:**

  Independent
  - ☐ Address safety in home - Physical, emotional and sexual abuse
  - ☐ System to prepare medications, Dr. appointments
  - ☐ Adaptive equipment
  - ☐ Assistance at home: Home health, R.N., C.N.A., P.T., O.T.
  - ☐ Has there been a home safety evaluation
  - ☐ Non-medical Care
  - ☐ Transportation
  - ☐ Mental Health Support

- ☐ **LONG TERM CARE NEEDS:**
  - ☐ Assisted Living/Skilled Nursing
  - ☐ Medication plan at facility
  - ☐ Response time - Are needs being met
  - ☐ Transportation, adaptive equipment
  - ☐ Hospice

- ☐ **CAREGIVER DISTRESS:**
  - ☐ Assessment of Partner's physical and emotional health,(abuse by P.W.P)
  - ☐ Resources in the community for respite care, Adult Day Care Programs
  - ☐ Support group for Caregiver
  - ☐ Mental health support system Psychologist, counselor
  - ☐ Back up plan to care for P.W.P. if unable to.

- ☐ **FINANCIAL ISSUES AND CONCERNS:**
  - ☐ Employed currently - Assess if have short term - long term insurance
  - ☐ Health Insurance coverage
  - ☐ Medicare
  - ☐ Medicaid eligibility
  - ☐ Qualifications for Social Security Disability Income or Supplemental Security Income

☐ **If moved to Assisted Living, S.N.F., or Hospice since last visit, address eligibility for qualifications for Medicaid.**

☐ **PLAN:**

Referrals for resources in the community:

- ☐ Elder law attorney for Power of Attorney, Guardianship, Conservatorship, finances, Disability Denials
- ☐ Funding assistance for medications, medical equipment
- ☐ Support groups
- ☐ Local Parkinson Association
- ☐ Counselors, Psychologists for chronic illness
- ☐ If needed, local county Adult Protection
- ☐ Provide educational forums on Parkinson's in the community
- ☐ Web sites: Michael J. Fox, Davis Phiney Foundation and National Parkinson Association

Signature: ______________________________ **Date:** ______________

SPIRITUAL CARE CHECKLIST: Initial and Return* Visits v1.0 11/11/15

REDCap ID/Study ID: ___________ / ___________ Date: ________________

☐ **Spiritual Wellbeing**

- ☐ Anger, frustration
- ☐ Fear, anxiety
- ☐ Guilt, feeling like a burden
- ☐ Grief, sadness, depression
- ☐ Demoralization, sense of helplessness

☐ **Sources of Stress**

- ☐ Physical
- ☐ Emotional
- ☐ Social
- ☐ Spiritual

☐ **Sources of Meaning**

- ☐ Purpose, happiness, gratitude, and joy
- ☐ Belief system, values, importance of faith and/or spirituality

☐ **Sources of Support**

- ☐ Primary caregiver
- ☐ Family
- ☐ Other relationships
- ☐ Faith group, friends, and/or community groups
- ☐ Personal strengths and positive qualities
- ☐ Stress reduction practices and activities
- ☐ Spiritual practices and/or rituals

☐ **Hopes**

- ☐ Goals and hopes
- ☐ Life completion tasks
- ☐ Personal and relational life closure

☐ **Care Plan and Recommendations**

- ☐ Continued spiritual support
- ☐ Referral to others: counselor, faith and/or support group
- ☐ Education on practices and activities, coping

***For follow-up/return visits: Review for any changes**

Signature: ______________________________ **Date:** ________________

NURSE CHECKLIST – New and Return Visits

v1.0 11/11/15

REDCap ID/Study ID: __________ / __________ **Date: ________________**

- ☐ **Medication reconciliation at beginning of visit**
- ☐ **Primary concerns for patient** (What should we focus on today?)
- ☐ **Primary concerns for caregiver** (if present)
- ☐ **Health care proxy designation and documentation**
- ☐ **Advanced care planning and documentation**
- ☐ **Home safety and home health care needs**
- ☐ **Assess for home palliative care or hospice needs**
- ☐ **Nutritional status and diet**
- ☐ **MOCA (**for baseline, 6 and 12 month visits)
- ☐ **For high risk patients [e.g., bedbound or incontinent]**
  - ☐ Assess skin integrity
  - ☐ Need for home care for wound or skin care

Signature: ________________________ **Date: ______________**
