# Supplementary material for: Comparison of Integrated Outpatient Palliative Care With Standard Care in Patients With Parkinson Disease and Related Disorders: A Randomized Clinical Trial
Source: JAMA Neurol. 2020 Feb 10;77(5):1–11. doi: 10.1001/jamaneurol.2019.4992 (PMC7042842; doi:10.1001/jamaneurol.2019.4992)
Supplement: Supplement 2. — eMethods 1. Palliative Care Needs Assessment Tool–Parkinson Disease eMethods 2. Detailed Description of Outpatient Palliative Care Intervention: Training and Experience of the Integrated Palliative Care Team, Logistics of Integrated Palliative Care Clinic Visits, and Goals of Integrated Palliative Care Visits and Team Member Roles eMethods 3. Neurologist, Social Worker, Spiritual Care, and Nurse Visit Checklists eMethods 4. Patient Health Care Utilization and Caregiver Health Care Utilization Questionnaires eResults. Additional Sensitivity Analyses, Subgroup Analyses, and Health Care Use Analyses eTable 1. Raw Differences Between Groups in Primary and Secondary Outcomes, Summary Statistics eTable 2. Differences Between Groups in Primary and Secondary Outcomes, Inferential Statistics, Unadjusted eTable 3. Snapshot Models for Patients for the 6-Week Periods Proceeding Baseline, 6 Months, and 12 Months eTable 4. Cumulative Models for Patients for 6 Months and 12 Months, Patients With Complete Data Only eReferences [file jamaneurol-77-551-s002.pdf]

Supplementary Online Content

Kluger BM, Miyasaki J, Katz M, et al. Comparison of integrated outpatient palliative care with standard care in patients with Parkinson disease and related disorders: a randomized clinical trial. *JAMA Neurol*. Published February 10, 2020. doi:10.1001/jamaneurol.2019.4992

eMethods 1. Palliative Care Needs Assessment Tool—Parkinson Disease

eMethods 2. Detailed Description of Outpatient Palliative Care Intervention: Training and Experience of the Integrated Palliative Care Team, Logistics of Integrated Palliative Care Clinic Visits, and Goals of Integrated Palliative Care Visits and Team Member Roles

eMethods 3. Neurologist, Social Worker, Spiritual Care, and Nurse Visit Checklists

eMethods 4. Patient Health Care Utilization and Caregiver Health Care Utilization Questionnaires

eResults. Additional Sensitivity Analyses, Subgroup Analyses, and Health Care Use Analyses

eTable 1. Raw Differences Between Groups in Primary and Secondary Outcomes, Summary Statistics

eTable 2. Differences Between Groups in Primary and Secondary Outcomes, Inferential Statistics, Unadjusted

eTable 3. Snapshot Models for Patients for the 6-Week Periods Proceeding Baseline, 6 Months, and 12 Months

eTable 4. Cumulative Models for Patients for 6 Months and 12 Months, Patients With Complete Data Only

eReferences

This supplementary material has been provided by the authors to give readers additional information about their work.

©2020 Kluger BM et al. *JAMA Neurology*.

eMethods 1. Palliative Care Needs Assessment Tool—Parkinson Disease

STUDY ID#:_______________________________ **DATE:** ________________

Section 1a: Red Flags – If present, be alert for unmet palliative care need:	Y	N
Red flag symptoms? (Persistent hallucinations / ≥2 Falls / Aspiration / Hoehn and Yahr 3 -see guidance for details)		
Admission to residential or nursing care?		
Section 1b: Priority referral for further assessment:	Y	N
No carer?		

**Section 2: PATIENT WELLBEING** (*"Does the patient have……"*)	Level of Concern		
Who provided this information?<br>Patient ☐ Carer ☐ Both☐	None	Some/ Potential	Significant
Unresolved physical symptoms? (Motor / Non-motor)			
Unresolved psychological or neuropsychiatric symptoms?			
Problems with daily living activities?			
Spiritual or existential concerns?			
Financial or legal concerns?			
Health beliefs, cultural or social factors making care delivery complex?			
**Information needs**: Prognosis ☐ Diagnosis ☐ Treatment options ☐<br>Financial/legal issues ☐ Support services ☐ Social/emotional issues ☐			

**Section 3: ABILITY OF CARER OR FAMILY TO CARE FOR PATIENT** (*"Is the Carer / Family……."*)			
	Level of Concern		
Who provided this information?<br>Patient ☐ Carer ☐ Both☐	None	Some/ Potential	Significant
Distressed about the patient's symptoms? (Motor / Non-motor / neuro-psychiatric)			
Having difficulty providing physical care?			
Having difficulty coping?			
Concerned about financial or legal issues?			
Experiencing problems that are interfering with inter-personal relationships or functioning?			
**Information needs**: Prognosis ☐ The diagnosis ☐ Treatment options ☐<br>Financial/legal issues ☐ Support services ☐ Social/emotional issues ☐			

**Section 4: CARER/FAMILY WELLBEING "Carer or family experiencing……."**	Level of Concern		
	None	Some/ Potential	Significant
Problems that are interfering with their wellbeing or functioning?			
Grief over the impending or recent death of the patient?			

Comments:

©2020 Kluger BM et al. *JAMA Neurology*.

ISSUES TO CONSIDER WHEN RATING LEVEL OF CONCERN

RED FLAGS – If present consider further assessment by own team +/- SPCS if required

Visual hallucinations –"yes" if hallucinations are formed and persistent. Do not include if associated with inter-current illness or medication change.
Recurrent falls –"yes" if multiple (>2) falls have occurred. Single, isolated falls, even in the context of injury, should not be included here.
Pneumonia / choking – May indicate aspiration and should trigger further palliative review in addition to SLT where appropriate.
Hoehn and Yahr stage 3 (Bilateral disease, mild to moderate disability and impaired postural reflexes) is associated with reduced quality of life.
24 hour care – admission to either residential or nursing care should trigger exploration of further supportive and palliative care needs.
Failure to attend clinic – may indicate physical or social difficulties, change in circumstance or acute admission preventing attendance.

PATIENT WELLBEING

Physical symptoms
• Fatigue, drowsiness, Pain, Constipation, Poor sleep, Urinary urgency, frequency, or incontinence, swallowing difficulty, SOB, Drooling, Spasms.
Activities of daily living
• Is the patient having difficulty with toileting, showering, bathing, or food preparation?
• Do they require more information to maximise their daily function –see below
Neuro-psychiatric / Psychological - "Does the patient have…."
• Thinking or memory problems, which interfere with wellbeing / relationships?
• Hallucination or behavioural issues which require assistance or evidence of psychosis?
• Sustained lowering of mood, tearfulness or guilt? Loss of pleasure in usual activities? Feelings of anxiety, apprehension, anger or fearfulness?
• Is the patient struggling with the implications of, or emotional response to the diagnosis?
• Is the patient requesting a hastened death?
Spiritual/Existential – "Is the patient…."
• Feeling isolated or hopeless?
• Feeling that life has no meaning or that his/her life has been wasted?
• Having difficulty thinking about the future?
• Requiring assistance in finding appropriate spiritual resources or services?
Financial/Legal concerns
• Consider loss of income, costs of treatment, travel expenses, equipment, or future care needs (such as residential care)?
• Is the family socio-economically disadvantaged?
• Is the patient or family aware of the various financial schemes available and do they need assistance in accessing these (e.g. social worker)?
• Are there conflicting opinions between patient and family relating to legal issues such as end-of-life care options and advance care plans?
Health Beliefs, Social and Cultural – "Does the patient or family…"
• Have beliefs or attitudes that make health care provision difficult – for example believing that palliative / hospice care is not available to them?
• Have communication difficulties – consider language and disease related issues (hypophonia / freezing of speech)?
• Feel socially isolated? If so are they avoiding peer support groups due to concerns such as "downward comparison"?
• Need information passed on to a particular member of the family or cultural group?
• Want information about prognosis to be withheld from the patient, or are they reluctant to discuss prognosis? If so, has this been explored?
• Have logistical difficulties accessing services (distance, transport, cost)?
Information – "Is the patient aware of/that…."
• Available services and do they need assistance accessing these? (e.g. financial /legal assistance, psychological services, support groups, pastoral care.)
• Advance Care Planning (ACP) and have their views / attitudes towards it been explored?
• PD is progressive, incurable and shortens life?
• Does the patient want more information about the course and prognosis of the disease and treatment options?

ABILITY OF CARER OR FAMILY TO CARE FOR PATIENT

Physical symptoms
• Are the patient's physical symptoms causing the carer and/or family distress?
Providing physical care
• Is the carer having difficulty coping with activities of daily living, medical regimes or practical issues such as equipment and transport?
• Have they received all the practical information they require?
Neuro-psychiatric / Psychological – "Is the carer / family…"
• Having difficulty coping with the patient's memory problems, hallucinations or behavioural issues?
• Having difficulty coping with the patient's psychological symptoms (esp. anxiety and depression)?
• Requesting a hastened death for the patient?
Family and Relationships
• Is there any communication breakdown or conflict between the patient and family over prognosis, treatment options or care giving roles?
• Is the patient particularly concerned about the impact of the illness on the carer or family?
• Is the disease having an adverse effect on the relationship between patient and carer? (May wish to consider impact of physical, psychological and personal cares, sexual dysfunction and change to previous roles within relationship.)
Information – "Do/are the carer or family…"
• Require more information about the course and prognosis of the disease and treatment?
• Aware of available services/ need assistance accessing these? (e.g. Financial/legal psychological services, support groups, pastoral care.)
• Are the information needs of the patient and family congruous?

©2020 Kluger BM et al. *JAMA Neurology*.

CARER AND FAMILY WELLBEING - "Do the carer or family…"
**Physical and psychosocial**
• Experience physical strain, ill health, fatigue, disturbed sleep? Is there evidence of anxiety, depression or feelings of hopelessness?
• Have spiritual/existential issues that are of concern?
• Currently feel that caring has a net positive or negative affect for them personally and their relationship with the patient?
**Grief (pre and post death)**
• Experience intrusive images, severe emotion, denial of implications of loss to self and neglect of necessary adaptive activities at home or work?
•Know of the progressive nature of PD? Has future care planning been considered? (If patient not capacitous this may include best interest decisions)
• Have access to support services (Such as PD Nurse Specialist, SPC, Local support groups, post bereavement support?)

©2020 Kluger BM et al. *JAMA Neurology*.

eMethods 2. Detailed Description of Outpatient Palliative Care Intervention: Training and Experience of the Integrated Palliative Care Team, Logistics of Integrated Palliative Care Clinic Visits, and Goals of Integrated Palliative Care Visits and Team Member Roles

In the review of the original version of this manuscript and our PCORI Final Research Report, Reviewers requested additional details on our intervention. Some of these details can be found in a manuscript on the implementation of Neuropalliative care clinics and our study protocol,[1,2] however other relevant details prompted by Reviewer questions merit additional description.

Training and Experience of the Integrated Palliative Care Team

The palliative medicine physician was the only clinician with formal palliative care training and board certification. The chaplain, social worker and nurse had traditional training for their respective professions and had experience working with Parkinson's disease (PD) as well as working in our Neuropalliative care clinics. In general, we found that training related to their field was easily translated into the outpatient palliative care (PC) context and these professions do not typically receive additional formal training before working in other PC contexts.

Our neurologists had all completed movement disorders fellowships but did not have formal or fellowship training in PC. Notably, the growth of each clinic involved input from a palliative medicine physician who assisted the PD team in shaping and formalizing their clinic. This palliative medicine partner provided formal coaching and mentoring as well as less formal education through curbside consults, interdisciplinary discussions, and care of shared patients. Of note, as these clinics evolved, so too did the involvement of the palliative medicine physician such that their time and input could be reduced and/or their efforts more focused as the palliative neurologist developed greater comfort and skills in key aspects of PC for PD.

Informal training for our palliative neurologists also included the following:

- PC workshops offered at national and international neurology meetings (American Academy of Neurology; International Movement Disorders Society)

PC courses for non-palliative medicine physicians (VitalTalk – [https://www.vitaltalk.org], Education in Palliative and End-of-life Care [https://www.bioethics.northwestern.edu/education/epec.html])

- Visits and shadowing other outpatient PC programs (neurology, cancer, heart failure)
- Learning by teaching (e.g. preparing lectures on PC-related topics for residency and fellowships)
- Learning by planning (e.g. preparing checklists for this study, quality improvement projects)
- Learning by doing (e.g. leading goals of care discussions, advance care planning, nonmotor symptom management) and through interactions with other interdisciplinary team members (e.g. learning about caregiver support from social worker, grief from chaplain…)

Logistics of Integrated Palliative Care Clinic Visits

As a pragmatic trial, our goal was to compare the effectiveness of two currently available treatment options, namely standard care from a neurologist and PCP, and outpatient Neuropalliative care which was available at a handful of academic centers at the time this trial started. We thus wanted to strike a balance between acknowledging the unique styles of each academic sites' team model and achieving fidelity, replicability and standards across sites. To find this balance we used Checklists (see Appendix 2) to ensure all teams were covering similar material but allowed flexibility for individual clinicians and teams in how they addressed items on the checklist. Of note, team members at all sites contributed to these checklists and feedback on the utility of checklists in improving the quality and consistency of care was high (They are still being used outside of this study at all sites). Visits typically lasted 2.5 hours for new and 2 hours for return patients. Distinguishing features of each clinic were as follows:

University of Alberta: Visits at the University of Alberta tended to follow a team-visit model where all

©2020 Kluger BM et al. *JAMA Neurology*.

members of the team, including the palliative medicine physician, would see the patient and caregiver at the same time. Scales and other data (e.g. medication reconciliation) would be captured by the nurse, initial history would be led by the palliative neurologist, and other team members would contribute as the visit progressed to make sure that items on their checklists were covered. A key strength of this model was high interdisciplinary interaction and no need for patients to repeat their story; a disadvantage is lower efficiency in terms of individual clinicians' time.

University of Colorado: Visits at the University of Colorado followed a sequential model, where patients/caregivers would stay in a room and the physician, chaplain, social worker and nurse would rotate in and out. Each team member used their own checklist and meetings in the AM, mid-day and end of the day, as well as brief verbal handoffs and written notes on a shared whiteboard allowed for interdisciplinary communication. The palliative medicine physician (JK) very rarely saw patients but did provide input for curbside consults and was highly involved in the development of the clinic. This included reviewing notes and meeting with the team monthly during the first year of the study to provide guidance and feedback. Advantages of this model included efficiency and greater one-on-one time with each discipline; a disadvantage was greater effort needed to maintain interdisciplinary communication and the potential that patients/caregivers may have to repeat certain parts of their story.

University of California San Francisco (UCSF): Visits at UCSF were generally sequential but would occasionally involve more than one member of the team seeing a patient/caregiver. The palliative medicine physician was present in clinic once a month to see patients handpicked by the team as having greater PC Needs, generally complex symptom management, especially pain, or more complex discussions around goals of care. The UCSF team was also the most frequent user of telemedicine as they had developed greater experience with this model of care through research and through their VA Neuropalliative care clinic.

Goals of Integrated Palliative Care Visits and Team Member Roles

Below we list the general goals of the integrated PC team as well as key team members when notable. Please also see Appendix 2 for detailed checklists for each team member.

- Understanding sources of suffering for the patient and caregiver. These sources, based on Cicely Saunder's concept of Total Pain,[3] could include medical/psychiatric symptoms, difficult emotions (commonly grief, guilt, loneliness and worries about the future), spiritual/existential distress, social challenges, or practical/financial concerns.
- Identifying opportunities for joy for the patient and caregiver. This exploration included understanding sources of meaning, love, and pleasure as well as understanding the patient and caregivers' life prior to the illness.
- Establishing goals of care including both priorities for patient and caregiver's current life (e.g. spending more time with family, getting out of the house, engaging in meaningful activities such as church or being in nature) and potential future needs (e.g. staying at home, options for avoiding unwanted procedures and hospital stays, back-up plans should anything affect primary caregivers ability to provide care).
- Providing education regarding the patient's diagnosis, as needed, as well as a road map for future potential challenges and prognosis (this was brought up by the team and patients and caregivers could direct how much detail they wanted)
- Anticipatory guidance, advance care planning and completion of related paperwork (primarily palliative neurologist with assistance from other team members)
- Systematic assessment and treatment of nonmotor symptoms (primarily palliative neurologist with assistance from other team members [e.g. mindfulness techniques to deal with pain from chaplain]); Palliative neurologist would also assess motor symptoms and could provide suggestions for management to primary neurologist if available.
- Referral for other services, including home-based therapies, outpatient therapies, home palliative

©2020 Kluger BM et al. *JAMA Neurology*.

care or hospice as appropriate

- Caregiver wellbeing, needs, self-care and support (primarily social worker, often with help from chaplain regarding difficult emotions)
- Difficult emotions, existential or spiritual concerns and means of building resilience (primarily chaplain)
- Medication reconciliation, initial triage, skin care (if patient chair or bedbound), nutrition, setting up home health services, completion of paperwork, integration of team recommendations (primarily nurse)
- Palliative medicine physician involvement tended to focus on complex symptom management, especially pain, and complex goals of care discussions
- Help with care coordination including sending notes to other members of the patient's healthcare team, calling other members of their team for more urgent matters (e.g. coordinating prescribing for pain and controlled substances), providing patients and caregivers with summaries of care and reaching out to patients/caregivers when relevant with follow-up calls

©2020 Kluger BM et al. *JAMA Neurology*.

NEUROLOGIST CHECKLIST – Initial Visit

v1.0 11/11/15

REDCap ID/Study ID:___________/ _________ **Date: ______________**

- ☐ Complete Medical History
  - History of Parkinson's disease
    - o Recent hospitalizations or other complications
  - Patient and Caregiver Goals of Care
    - o How define quality of life
    - o Worries about the future/Fears
    - o Sources of support and meaning
    - o If caregiver present – any issues of safety, overwhelmed, burnt-out, own health concerns
    - o Educational needs
    - o Status of Advance Care Plans/Documentation
    - o Specific Goals of Care
  - Review of Systems
    - o Psychiatric Symptoms (Anxiety and Depression)
    - o Behavioral Issues and Psychosis
    - o Edmonton Symptoms Assessment Scale PD
    - o Falls, balance and mobility
    - o Swallowing, sialorrhea and nutrition (weight changes)
    - o Other Symptoms
  - Review Medications
  - Allergies and medication side effects
  - Past Medical and Surgical History
  - Social History
    - o Current Living Situation
  - Family History
- ☐ Physical Examination
  - Review orthostatic vital signs and weight
  - Motor exam (UPDRS)
- ☐ Assessment
  - Review Prognosis and Disease Stage
  - Reflect Goals
- ☐ Plan
  - Safety issues (e.g. home safety, abuse, driving)
  - Medication changes
  - Referrals for other services
  - Heads up to other team members
  - o Need for Palliative Care Referral (in clinic or outside)
    - o Complex symptom management (e.g. pain)
    - Follow-up plan including issues for phone call

Signature: ______________________________ **Date:** ___________________

©2020 Kluger BM et al. *JAMA Neurology*.

NEUROLOGIST CHECKLIST – Return Visit

v1.0 11/11/15

REDCap ID/Study ID:___________/ __________ **Date: _______________**

- ☐ Interval Medical History
  - o Any interval events (e.g. UTI)
  - o Changes in functional status
  - o Patient and Caregiver Goals of Care
    - o Any changes/updates needed to goals
    - o Any new issues from caregiver perspective
  - o Review of Systems
    - o Psychiatric Symptoms (Anxiety and Depression)
    - o Behavioral Issues and Psychosis
    - o Edmonton Symptoms Assessment Scale PD
    - o Falls, balance and mobility
    - o Swallowing, sialorrhea and nutrition
    - o Other Symptoms
  - o Review Medications
  - o Social History
    - o Changes in living situation
- ☐ Physical Examination
  - o Any change in weight; repeat orthostatic vital signs if relevant
  - o Motor exam (UPDRS)
- ☐ Assessment
  - o Review Prognosis and Disease Stage
  - o Reflect Goals
- ☐ Plan
  - o Safety issues (e.g. home safety, abuse, driving)
  - o Medication changes
  - o Referrals for other services
  - o Heads up to other team members
  - o Need for Palliative Care Referral (in clinic or outside)
    - o Complex symptom management (e.g. pain)
  - o Follow-up plan including issues for phone call

Signature: ________________________________ **Date**: _______________

©2020 Kluger BM et al. *JAMA Neurology*.

REDCap ID/Study ID:___________/ __________ Date: _____________

☐ **SUPPORT SITUATION FOR PATIENT:**

Partner
Family
Friends
Neighbors

☐ ASSESSMENT OF CURRENT LIVING ENVIRONMENT:

Independent

Address safety in home - Physical, emotional and sexual abuse
System to prepare medications, Dr. appointments
Adaptive equipment
Assistance at home: Home health, R.N., C.N.A., P.T., O.T.
Has there been a home safety evaluation
Non-medical Care
Transportation
Mental Health Support

☐ LONG TERM CARE NEEDS:

Assisted Living/Skilled Nursing
Medication plan at facility
Response time: Are needs being met
Transportation, adaptive equipment
Hospice

☐ CAREGIVER DISTRESS:

Assessment of Partner's physical and emotional health,(abuse by P.W.P)
Resources in the community for respite care, Adult Day Care Programs
Support group for Caregiver
Mental health support system, i.e. Psychologist, counselor
Back up plan to care for P.W.P. if unable to.

☐ FINANCIAL ISSUES AND CONCERNS:

Employed currently - Assess if have short term - long term insurance
Health Insurance coverage
Medicare
Medicaid eligibility
Qualifications for Social Security Disability Income or Supplemental Security Income

©2020 Kluger BM et al. *JAMA Neurology*.

☐ PLAN:

Referrals for resources in the community:

- Elder law attorney for Power of Attorney, Guardianship, Conservatorship, finances, Disability Denials
- Funding assistance for medications, medical equipment
- Support groups
- Local Parkinson Association
- Counselors for chronic illness
- If needed, local county Adult Protection
- Provide educational forums on Parkinson's in the community
- Web sites: Michael J. Fox, Davis Phiney Foundation and National Parkinson Association

☐ Follow up phone call to address further needs of patient and family.

Signature: ______________________________ Date: ___________________

©2020 Kluger BM et al. *JAMA Neurology*.

REDCap ID/Study ID:___________/ __________ Date:_________________

☐ REVIEW OF SUPPORT SYSTEM CHANGES:

Partner
Family
Friends
Neighbors

☐ CURRENT LIVING SITUATION:

Independent

Address safety in home - Physical, emotional and sexual abuse
System to prepare medications, Dr. appointments
Adaptive equipment
Assistance at home: Home health, R.N., C.N.A., P.T., O.T.
Has there been a home safety evaluation
Non-medical Care
Transportation
Mental Health Support

☐ LONG TERM CARE NEEDS:

Assisted Living/Skilled Nursing
Medication plan at facility
Response time - Are needs being met
Transportation, adaptive equipment
Hospice

☐ CAREGIVER DISTRESS:

Assessment of Partner's physical and emotional health,(abuse by P.W.P)
Resources in the community for respite care, Adult Day Care Programs
Support group for Caregiver
Mental health support system Psychologist, counselor
Back up plan to care for P.W.P. if unable to.

☐ FINANCIAL ISSUES AND CONCERNS:

Employed currently - Assess if have short term - long term insurance
Health Insurance coverage
Medicare
Medicaid eligibility
Qualifications for Social Security Disability Income or Supplemental Security Income

☐ If moved to Assisted Living, S.N.F., or Hospice since last visit, address eligibility for qualifications for Medicaid.

©2020 Kluger BM et al. *JAMA Neurology*.

☐ PLAN:

Referrals for resources in the community:

Elder law attorney for Power of Attorney, Guardianship, Conservatorship, finances, Disability Denials
Funding assistance for medications, medical equipment
Support groups
Local Parkinson Association
Counselors, Psychologists for chronic illness
If needed, local county Adult Protection
Provide educational forums on Parkinson's in the community
Web sites: Michael J. Fox, Davis Phiney Foundation and National Parkinson Association

Signature: ______________________________________ **Date:** __________________

©2020 Kluger BM et al. *JAMA Neurology*.

SPIRITUAL CARE CHECKLIST: Initial and Return* Visits

v1.0 11/11/15

REDCap ID/Study ID:___________/ ____________ **Date: ________________**

☐ **Spiritual Wellbeing**

Anger, frustration
Fear, anxiety
Guilt, feeling like a burden
Grief, sadness, depression
Demoralization, sense of helplessness

☐ **Sources of Stress**

Physical
Emotional
Social
Spiritual

☐ **Sources of Meaning**

Purpose, happiness, gratitude, and joy
Belief system, values, importance of faith and/or spirituality

☐ **Sources of Support**

Primary caregiver
Family
Other relationships
Faith group, friends, and/or community groups
Personal strengths and positive qualities
Stress reduction practices and activities
Spiritual practices and/or rituals

☐ **Hopes**

Goals and hopes
Life completion tasks
Personal and relational life closure

☐ **Care Plan and Recommendations**

Continued spiritual support
Referral to others: counselor, faith and/or support group
Education on practices and activities, coping

***For follow-up/return visits: Review for any changes**

Signature: ___________________________________ **Date:** __________________

©2020 Kluger BM et al. *JAMA Neurology*.

NURSE CHECKLIST – New and Return Visits **v1.0 11/11/15**

REDCap ID/Study ID:____________/_____________ Date: ____________________

- ☐ **Medication reconciliation at beginning of visit**
- ☐ **Primary concerns for patient** (What should we focus on today?)
- ☐ **Primary concerns for caregiver** (if present)
- ☐ **Health care proxy designation and documentation**
- ☐ **Advanced care planning and documentation**
- ☐ **Home safety and home health care needs**
- ☐ **Assess for home palliative care or hospice needs**
- ☐ **Nutritional status and diet**
- ☐ **MOCA (**for baseline, 6 and 12 month visits)
- ☐ **For high risk patients [e.g., bedbound or incontinent]**
  - Assess skin integrity
  - Need for home care for wound or skin care

Signature: ______________________________ Date: __________________

©2020 Kluger BM et al. *JAMA Neurology*.

eMethods 4. Patient Health Care Utilization and Caregiver Health Care Utilization Questionnaires

PATIENT HEALTH CARE UTILIZATION – **BASELINE** VISIT Rev 5/17/16

FIRST SECTION: The following questions are about **health care services** received in the last 6 weeks. **(***Questions may be asked of the caregiver or the patient.)*

PT1. During the past 6 weeks, how many times have you gone to a hospital emergency room? (*This includes emergency room visits that resulted in a hospital admission.*)

___None
___One or more
If One or more, **How Many? ______________**
___Declined
___Don't know

PT2. During the past 6 weeks, how many times have you been a patient in a hospital overnight? (*This includes hospital admissions as a result of going to an emergency room.*)

___None **(SKIP: Go to question PT6)**
___One or more
If One or more, **How Many? ______________**
Then Ask questions ***PT3, PT4, and PT5)***
___Declined **(SKIP: Go to question PT6)**
___Don't know **(SKIP: Go to question PT6)**

If PT2 is one or more, ask follow-up questions ***PT3, PT4, PT5:***

PT3. During any of those hospital stays did you spend any time in an intensive care or critical care unit?

___No
___Yes
___Declined
___Don't know

PT4. During any of those hospital stays did you use life support equipment, such as a respirator?

___No
___Yes
___Declined
___Don't know

PT5. During any of those hospital stays did you receive antibiotics to treat pneumonia?

___No
___Yes

___Declined
___Don’t know

PT6. During the past 6 weeks, how many days or nights have you been a patient in a nursing home or other long-term care facility?

___None
___One or more days or nights
If One or more days or night, **How Many? ___________**
___Declined
___Don't know

PT7. During the past 6 weeks, did any medically-trained person come to your home to help you? *We only want to include help given to you by outside medically-trained personnel, not help provided to you by your caregiver. Include hospice care received at home.* (*Definition: medically-trained persons include professional nurses, visiting nurse's aides, physical or occupational therapists, chemo therapists, respiratory oxygen therapists, and hospice caregivers*)

___No **(SKIP: Go to question PT10)**
___Yes ***(If Yes: Ask follow-up questions: PT8 & PT9)***
___Declined **(SKIP: Go to question PT10)**
___Don't know **(SKIP: Go to question PT10)**

If PT7 is Yes, ask follow-up questions ***PT8 and PT9****:*

PT8. Which of the following medical services did they provide?
(Check all that apply)
___Nursing
___Physical therapy
___Occupation therapy
___Respiratory therapy
___Chemo therapy
___Hospice care
___Other-**Describe**:________________
___Don’t know

PT9. On average over the past 6 weeks, how many times did a medically-trained person come to your home to help you during a typical week?
___None
___One or more times
If One or more, **How Many? ______________**
___Declined
___Don't know

©2020 Kluger BM et al. *JAMA Neurology*.

PT10. In the past 6 weeks, did you use any special facility or services that we haven't talked about, such as an adult day care center or an outpatient rehabilitation program?

___No **(SKIP: Go to question PT13)**
___Yes ***(If YES, ask follow-up questions PT11 and PT12)***
___Declined **(SKIP: Go to question PT13)**
___Don't know **(SKIP: Go to question PT13)**

*If PT10 is Yes, ask follow-up questions **PT11 and PT12**:*

PT11. Which special facility or services did he/she use?
(Check all that apply)
___Adult day care center
___Outpatient rehabilitation
___Other (*Record description*:_________________________________)
___Declined
___Don't know

PT12. On average over the past six weeks, how many times did you use the special facility or services to help him/her during a typical week?
____(Record Number) Number of times
___Declined
___Don't know

PT13. During the past 6 weeks, how many times have you seen or talked to a social worker or religious/spiritual counselor *(Include both home and/or office visits)?*

___None
____(***Record number***) Number of times
___Declined
___Don't know

PT14-Question not applicable at Baseline Visit. Next question is PT 15.

NEXT SECTION: The next questions are about **falling down**:

PT15. During the past 6 weeks, have you fallen down?

___No **(SKIP: Go to question PT21)**
___Yes ***(If YES, ask the follow-up questions PT16 and PT17)***
___Declined **(SKIP: Go to question PT21)**
___Don't know **(SKIP: Go to question PT21)**

*If PT14 is Yes, ask follow-up questions **PT16 and PT17:***

PT16. How many times have you fallen down in the past 6 weeks?

___One or more
If One or more, **How Many?_________**
___Declined
___Don't know

PT17. In that fall/In any of these falls, did you injure yourself seriously enough to need medical treatment?

___No **(SKIP: Go to question PT21)**
___Yes (If YES, ask follow-up questions PT18 thru PT20)
___Declined **(SKIP: Go to question PT21)**
___Don't know **(SKIP: Go to question PT21)**

PT18. Did you visit your doctor because you were hurt from the fall?

___No
___Yes
___Declined
___Don't know

PT19. Did you visit the emergency department because you were hurt from the fall?

___No
___Yes
___Declined
___Don't know

PT20. Were you admitted to the hospital because of the fall?

___No
___Yes
___Declined
___Don't know

©2020 Kluger BM et al. *JAMA Neurology*.

NEXT SECTION: The following questions are regarding **hospice care** the patient may have received during the past 6 weeks:

PT21. Other than the overnight stays you just told me about, during the past 6 weeks, how many nights have you spent in an inpatient hospice facility (*Such as a nursing, long-term care, or rehabilitation facility*)?

___None
___One or more nights
If One or more nights, **HowMany?** ___________
___Declined
___Don't know

PT22. Has there been any change in the palliative care services or hospice services that you have received over the last 6 weeks?

___No
___Yes
If yes, please explain:____
____

©2020 Kluger BM et al. *JAMA Neurology*.

NEXT SECTION: The following questions are regarding the patient's **daily activities and current work situation** during the past 7 days, not including today.

Ask ALL Patients to answer PT 28 (whether or not they are working):
(Note that PT 28 intentionally appears out of order to maintain original question numbering in REDCap.)

PT28. During the past 7 days, how much did your health problems affect your ability to do your regular daily activities, **other than work at a job**?

[By regular activities, we mean the usual activities you do, such as work around the house, shopping, childcare, exercising, studying, etc. Think about times you were limited in the amount or kind of activities you could do and times you accomplished less than you would like. If health problems affected your activities only a little, choose a low number. Choose a high number if health problems effected your activities a great deal.]

Consider only how much your health problems affected your ability to do your regular daily activities, other than work at a job.

CIRCLE A NUMBER BETWEEN 0 and 10:

0=Health problems had no effect on my daily activities											10=Health problems completely prevented me from doing my daily activities
	0	1	2	3	4	5	6	7	8	9	10

©2020 Kluger BM et al. *JAMA Neurology*.

PT23. Did you work at a job or business at any time over the past 7 days (*Include any temporary, part-time, or seasonal work if even for a few days)?*

___No **(Ask follow up question PT24.)**

___Yes **(SKIP: Go to questions PT25 thru PT 31)**

If PT 23 is No, ask follow up question PT24:

PT24. What was the main reason you were not working this past week? *(Read list only if respondent is having difficulty answering the question)*

___1 Ill or disabled and unable to work

___2 Taking care of home or family

___3 Going to school

___4 Retired

___5 No work available

___6 Other **(Specify:______________________________)**

---END OF INTERVIEW for patients that have not worked over the past 7 days.

If PT 23 is yes (patient HAS worked over the past 7 days), ask questions PT25-PT31:

PT25. During the past 7 days, how many hours did you actually work?

_____Hours *(Record number of hours)*

_____Don't know

PT26. During the past 7 days, how many hours did you miss from work because of your health problems (*Include hours you missed on sick days, times you went in late, left early, etc., but do not include time you missed to participate in this study)?*

_____Hours *(Record number of hours)*

_____Don't know

PT27. During the past 7 days, how much did your health problems affect your productivity while you were working?

[Think about days you were limited in the amount or kind of work you could do, days you accomplished less than you would like, or days you could not do your work as carefully as usual. If health problems affected your work only a little, choose a low number. Choose a high number if health problems effected your work a great deal.]

©2020 Kluger BM et al. *JAMA Neurology*.

Consider only how much your health problems effected productivity while you were working:

CIRCLE A NUMBER between 0 and 10:

0=Health problems had no effect on my work	0	1	2	3	4	5	6	7	8	9	10	10=Health problems completel prevented me from working

PT28 – Question was moved and appears after PT22.

PT29. Are you employed by government, by a PRIVATE company, a non-profit organization, or are you self-employed or working in a family business?

___Government
___Private for-profit company
___Non-profit organization including tax exempt and charitable organizations
___Self employed
___Working in family business
___Don't know

PT30. What kind of business or industry is this?

(*Select only one*)

___Manufacturing (e.g., food, beverage, wood or paper products, printing, petroleum/chemical)
___Construction (e.g., building, heavy/civil engineering)

___Natural Resources, agriculture and mining (e.g., crop/animal production, forestry/lopping, oil/gas extraction, mining)
___Retail (e.g., motor vehicle, furniture, electronics/appliance, food/beverage, gasoline station)
___Wholesale trade (e.g., durable, nondurable goods)

___Information (e.g., publishing, motion picture & sound, telecommunications, data processing)
___Financial activities (e.g., banking, credit, securities, insurance carriers)

___Education and Health Services (e.g., educational services, health care and social assistance)
___Something else (**Describe**:________________________)

PT31. Please choose the category that best describes your <u>main</u> job. *If none of the categories fit you exactly, please respond with the closest category to your experience.*

(Select only <u>one</u>.)

___Executive, administrator, or senior manager (e.g., CEO, sales VP, plant manager)

___Professional (e.g., engineer, accountant, systems analyst)

___Technical support (e.g., lab technician, legal assistant, computer programmer)

___Sales (e.g., sales representative, stockbroker, retail sales)

___Clerical and administrative support (e.g., secretary, billing clerk, office supervisor)

___Service occupation (e.g., security officer, food service worker, janitor)

___Precision production and crafts worker (e.g., mechanic, carpenter, machinist)

___Chemical/Production Operator (e.g., shift supervisors and hourly employees)

___Laborer (e.g., truck driver, construction worker)

---<u>END OF INTERVIEW</u> for patients that <u>HAVE worked</u> over the past 7 days

©2020 Kluger BM et al. *JAMA Neurology*.

CAREGIVER HEALTH CARE UTILIZATION – **BASELINE** VISIT

FIRST SECTION: The following questions are about your **health and the health care** you {caregiver} have received in the last 6 weeks.

CG1. During the past 6 weeks, how many times have you gone to a hospital emergency room about your own health? (*This includes emergency room visits that resulted in a hospital admission*)

___None
___One or more
If One or more, **How Many?** ______________
___Declined
___Don't know

CG2. During the past 6 weeks, how many times have you been a patient in a hospital overnight? (*This includes hospital admissions as a result of going to an emergency room.*)

___None
___One or more
If One or more, **How Many?** ______________
___Declined
___Don't know

CG3. During the past 6 weeks, how many times have you seen or talked to a medical doctor about your health, including clinic visits or house calls? (*Not counting the hospital or emergency room doctors.*)

___None
___One or more
If One or more, **How Many?** ______________
___Declined
___Don't know

CG4. During the past 6 weeks, how many times have you seen or talked to a mental health professional, such as a psychiatrist, psychologist, psychiatric nurse, or clinical social worker, about your own health? *(Not counting the visits you've already told me about.)*

___None
___One or more
If One or more, **How Many?** ______________
___Declined
___Don't know

©2020 Kluger BM et al. *JAMA Neurology*.

NEXT SECTION: The next questions are about "**non-medical**" services you might have received during the past 6 weeks.

CG5. During the past six weeks, how many times have you seen or talked to a social worker or religious/spiritual counselor? *(Including both home and/or office visits)*

___None
___One or more
If One or more, **How Many?** ______________
___Declined
___Don't know

CG6. Question not applicable at Baseline Visit. Next question is CG7.

CG7. During the past 6 weeks, has the patient used respite care (*which is a short-term temporary stay at a nursing facility to give you a break*)?

___None
___One or more
If One or more, **How Many?** ______________
___Declined
___Don't know

©2020 Kluger BM et al. *JAMA Neurology*.

NEXT SECTION: The next questions are about your {caregiver} **current work situation** during the past 7 days, not including today.

Ask ALL Caregivers to answer CG14 (whether or not they are working):
(Note that CG14 intentionally appears out of order to maintain original question numbering in REDCap.)

CG14. During the past 7 days, how much did the **patient's** health problems affect your ability to do your regular daily activities, other than work at a job?

By regular activities, we mean the usual activities you do, such as work around the house, shopping, childcare, exercising, studying, etc. Think about times you were limited in the amount or kind of activities you could do and times you accomplished less than you would like. If health problems affected your activities only a little, choose a low number. Choose a high number if health problems affected your activities a great deal.

Consider only how much health problems affected your ability to do your regular daily activities, other than work at a job.

CIRCLE A NUMBER between 0 and 10:

0=Health problems had effect on my daily activities	0	1	2	3	4	5	6	7	8	9	10	10=Health completely prevented me from doing my daily activities

CG8. Did you work at a job or business at any time over the past 7 days? (*Include temporary, part-time, or seasonal work even if for a few days)*

___Yes **(SKIP: Go to question CG10)**

___No (**If NO, then go to Follow-up question CG9)**

CG9. What was the main reason you did not working this past week?
(Read list only if respondent is having difficulty answering the question)

___1 Ill, or disabled and unable to work

___2 Taking care of home or family

___3 Going to school

___4 Retired

___5 No work available

___6 Other (**Specify:________________________________**)

(AFTER answering CG9 and caregiver is not currently working, SKIP to CG20.)

©2020 Kluger BM et al. *JAMA Neurology*.

Answer questions CG10 through CG 13 if caregiver IS currently working:

CG10. During the past 7 days, how many hours did you actually work?

_____Hours *(Record number of hours)*

_____Don't know

CG11. During the past 7 days, how many hours did you miss from work because of **your** health problems? (*Include hours you missed on sick days, times you went in late, left early, etc., because of your health problems. Do not include time you missed to participate in this study)*

_____Hours *(Record number of hours)*

_____Don't know

CG12. During the past 7 days, how many hours did you miss from work because of the **patient's** health problems? (*Include hours you missed, times you went in late, left early, etc., because of the patient's health problems or health care needs. Do not include time you missed to participate in this study)*

_____Hours *(Record number of hours)*

_____Don't know

CG13. During the past 7 days, how much did the **patient's** health problems affect **your** productivity **while you were working**?

Think about days you were limited in the amount or kind of work you could do, days you accomplished less than you would like, or days you could not do your work as carefully as usual. If health problems affected your work only a little, choose a low number. Choose a high number if health problems affected your work a great deal.

Consider only how much health problems affected productivity while you were working.

CIRCLE A NUMBER between 0 and 10:

0=Health problems had no effect on my work	0	1	2	3	4	5	6	7	8	9	10	10=Health problems completel prevented me from working

If the caregiver HAS been working in the past 7 days continue to CG15.

If the caregiver HAS NOT been working in the past 7 days go to question CG20.

©2020 Kluger BM et al. *JAMA Neurology*.

CG15. Are you employed by government, by a PRIVATE company, a non-profit organization, or are you self-employed or working in a family business?

___Government
___Private for-profit company
___Non-profit organization including tax exempt and charitable organizations
___Self-employed
___Working for the family business

CG16. What kind of business or industry is this?

(*Select only <u>one</u>*)

___Manufacturing (e.g., food, beverage, wood or paper products, printing, petroleum/chemical)
___Construction (e.g., building, heavy/civil engineering)

___Natural resources, agriculture and mining (e.g., crop/animal production, forestry/lopping, oil/gas extraction, mining)
___Retail (e.g., motor vehicle, furniture, electronics/appliance, food/beverage, gasoline station)
___Wholesale trade (e.g., durable, nondurable goods)

___Information (e.g., publishing, motion picture & sound, telecommunications, data processing)
___Financial activities (e.g., banking, credit, securities, insurance carriers)

___Education and health services (e.g., educational services, health care and social assistance)
___Something else (**Describe**:_______________________)

©2020 Kluger BM et al. *JAMA Neurology*.

CG17. Please choose the category that best describes your main job. *If none of the categories fits you exactly, please respond with the closest category to your experience.*

(Select only one.)

___Executive, administrator, or senior manager (e.g., CEO, sales VP, plant manager)

___Professional (e.g., engineer, accountant, systems analyst)

___Technical support (e.g., lab technician, legal assistant, computer programmer)

___Sales (e.g., sales representative, stockbroker, retail sales)

___Clerical and administrative support (e.g., secretary, billing clerk, office supervisor)

___Service occupation (e.g., security officer, food service worker, janitor)

___Precision production and crafts worker (e.g., mechanic, carpenter, machinist)

___Chemical/Production Operator (e.g., shift supervisors and hourly employees)

___Laborer (e.g., truck driver, construction worker)

The next questions are about your earnings from your current job.

CG18. Are you paid a regular salary or wages?
___Salary **(Ask Follow-up question CG19)**
___Wages **(Ask Follow-up question CG19)**
___Declined
___Don't know

CG19 About how much are you paid before taxes and other deductions?
Amount (**Specify $**______________)
___Declined
___Don't know

---END OF INTERVIEW for caregivers that have worked over the past 7 days.

©2020 Kluger BM et al. *JAMA Neurology*.

Question sequences below are for caregivers that <u>have NOT worked</u> in the past seven days.

The next questions are about the **<u>last</u> job** you had. We're interested in your situation near the end of this job.

CG20. In what month and year did you last work?

Month_____________
Year ______________
____Don't know

CG21. At your last job, were you employed by government, by a PRIVATE company, a non-profit organization, or were you self-employed or working in a family business?

___Government
___Private for-profit company
___Non-profit organization including tax exempt and charitable organizations
___Self employed
___Working for the family business
___Don't know

CG22. What kind of business or industry was this?

(Select only <u>one</u>)

___Manufacturing (e.g., food, beverage, wood or paper products, printing, petroleum/chemical)
___Construction (e.g., building, heavy/civil engineering)

___Natural resources, agriculture and mining (e.g., crop/animal production, forestry/lopping, oil/gas extraction, mining)
___Retail (e.g., motor vehicle, furniture, electronics/appliance, food/beverage, gasoline station)
___Wholesale trade (e.g., durable, nondurable goods)

___Information (e.g., publishing, motion picture & sound, telecommunications, data processing)
___Financial activities (e.g., banking, credit, securities, insurance carriers)

___Education and health services (e.g., educational services, health care and social assistance)
___Something else (**<u>Describe</u>**:________________________)

CG23. Please choose the category that best describes your main job. *If none of the categories fit you exactly, please respond with the closest category to your experience.*

(Select only one.)

___Executive, administrator, or senior manager (e.g., CEO, sales VP, plant manager)

___Professional (e.g., engineer, accountant, systems analyst)

___Technical support (e.g., lab technician, legal assistant, computer programmer)

___Sales (e.g., sales representative, stockbroker, retail sales)

___Clerical and administrative support (e.g., secretary, billing clerk, office supervisor)

___Service occupation (e.g., security officer, food service worker, janitor)

___Precision production and crafts worker (e.g., mechanic, carpenter, machinist)

___Chemical/Production operator (e.g., shift supervisors and hourly employees)

___Laborer (e.g., truck driver, construction worker)

The next two questions are about your earnings from that last job.

CG24. Were you paid a regular salary or wages?
___Salary **(Ask follow-up question CG25)**
___Wages **(Ask follow-up question CG25)**
___Declined
___Don't know

CG25. About how much were you paid before taxes and other deductions?
Amount (specify $_______________)
___Declined
___Don't know

---END OF INTERVIEW for caregivers that have not worked over the past 7 days

©2020 Kluger BM et al. *JAMA Neurology*.

eResults. Additional Sensitivity Analyses, Subgroup Analyses, and Health Care Use Analyses

Additional Sensitivity Analyses

QOL-AD

The same pattern of statistical significance remained when missing data was filled in with multiple imputation; 6 month estimated treatment effect was 1.82 (95% CI: (0.16, 3.47), p = 0.032), and 12 month estimated treatment effect was 1.26 (95% CI: (-0.20, 2.72), p = 0.091). QOL for patients and caregivers was jointly modeled so they could contribute information to each other. Factoring cross over in treatment made the estimated treatment effects stronger. For cross over models with covariate adjustment, but without missing data imputation of joint modeling, the estimated treatment effects were 2.48 (95% CI: (1.19, 3.76), p = 0.0002) for 6 months, and 1.87 (95% CI: (0.51, 3.24), p = 0.0074) for 12 months. When the imputed data and joint modeling is added in, the treatment effect estimates were 2.00 (95% CI: (0.52, 3.49), p = 0.009) for 6 months, and 1.48 (95% CI: (0.06, 2.91), p = 0.041).

ZBI

When missing data was filled in with multiple imputation the treatment effects were strengthened; 6-month estimated treatment effect was statistically significant at -2.63 (95% CI: (-4.46, -0.80), p = 0.006), and 12-month estimated treatment effect was -2.89 (95% CI: (-4.93, -0.85), p = 0.006). For cross over models with covariate adjustment, but without missing data imputation, the estimated treatment effects were -1.61 (95% CI: (-3.23, 0.01), p = 0.052) for 6 months, and -2.72 (95% CI: (-4.74, -0.71), p = 0.008) for 12 months. When the imputed data is added in, the treatment effect estimates were -2.64 (95% CI: (- 4.35, -0.93), p = 0.003) for 6 months, and -3.10 (95% CI: (-5.15, -1.05), p = 0.004).

Subgroup Analyses

PD (N = 185) vs. Atypical Parkinsonism (N = 25)

At 6 months the estimated treatment effect for the normal PD patients was 2.13, 95% CI (0.66, 3.59), p = 0.0046 for QOL-AD (patient perspective), and -1.08, 95% CI (-2.88, 0.72), p =

©2020 Kluger BM et al. *JAMA Neurology*.

0.2367 for ZBI. At 12 months the estimated treatment effect for the normal PD patients was 1.18, 95% CI (-0.24, 2.60), p = 0.1030 for QOL-AD (patient perspective), and -2.21, 95% CI (-4.33, -0.10), p = 0.0403 for ZBI.

At 6 months the estimated treatment effect for the abnormal PD patients was -0.55, 95% CI (-5.04, 3.94), p = 0.8102 for QOL-AD (patient perspective), and -5.39, 95% CI (-10.20, -0.58), p = 0.0285 for ZBI. At 12 months the estimated treatment effect for the abnormal PD patients was 2.90, 95% CI (-1.53, 7.33), p= 0.1977 for QOL-AD (patient perspective), and -5.17, 95% CI (-10.78, 0.43), p = 0.0700 for ZBI.

At 6 months the estimated difference in treatment effect between abnormal PD and normal PD patients was -2.68, 95% CI (-7.40, 2.05), p = 0.2653 for QOL-AD (patient perspective), and -4.31, 95% CI (-9.46, 0.84), p = 0.1001 for ZBI. At 12 months the estimated difference in treatment effect between abnormal PD and normal PD patients was 1.72, 95% CI (-2.93, 6.38), p = 0.4659 for QOL-AD (patient perspective), and -2.96, 95% CI (-8.97, 3.05), p = 0.3312 for ZBI.

Dementia (N = 62) vs. No Dementia (N = 148)

At 6 months the estimated treatment effect for the non-demented patients was 2.01, 95% CI (0.42, 3.60), p = 0.0133 for QOL-AD (patient perspective), and -1.22, 95% CI (-3.24, 0.81), p = 0.2360 for ZBI. At 12 months the estimated treatment effect for the non-demented patients was 1.68, 95% CI (0.11, 3.26), p = 0.0360 for QOL-AD (patient perspective), and -1.45, 95% CI (-3.78, 0.88), p = 0.2209 for ZBI.

At 6 months the estimated treatment effect for the demented patients was 1.33, 95% CI (-1.26, 3.93), p = 0.3122 for QOL-AD (patient perspective), and -2.67, 95% CI (-5.76, 0.42), p = 0.0894 for ZBI. At 12 months the estimated treatment effect for the demented patients was 0.43, 95% CI (-2.20, 3.06), p = 0.7463 for QOL-AD (patient perspective), and -5.21, 95% CI (-8.76, -1.66), p = 0.0044 for ZBI.

At 6 months the estimated difference in treatment effect between demented and non-demented

©2020 Kluger BM et al. *JAMA Neurology*.

patients was -0.68, 95% CI (-3.71, 2.35), p = 0.6584 for QOL-AD (patient perspective), and -1.46, 95% CI (-5.12, 2.21), p = 0.4335 for ZBI. At 12 months the estimated difference in treatment effect between demented and non-demented was -1.25, 95% CI (-4.28, 1.78), p = 0.4158 for QOL-AD (patient perspective), and - 3.76, 95% CI (-7.97, 0.45), p = 0.0798 for ZBI.

Advanced PD (Hoehn and Yahr > 3; N = 89) vs. Mild/Moderate (N = 121)

At 6 months the estimated treatment effect for the non-severe patients was 2.28, 95% CI (0.51, 4.05), p = 0.0199 for QOL-AD (patient perspective), and -1.64, 95% CI (-3.81, 0.53), p = 0.1364 for ZBI. At 12 months the estimated treatment effect for the non-severe patients was 1.66, 95% CI (-0.06, 3.37), p = 0.0588 for QOL-AD (patient perspective), and -2.10, 95% CI (-4.62, 0.43), p = 0.1034 for ZBI.

At 6 months the estimated treatment effect for the severe patients was 1.17, 95% CI (-1.11, 3.45), p = 0.3142 for QOL-AD (patient perspective), and -1.51, 95% CI (-4.24, 1.22), p = 0.2744 for ZBI. At 12 months the estimated treatment effect for the severe patients was 0.88, 95% CI (-1.38, 3.15), p = 0.4407 for QOL-AD (patient perspective), and -3.37, 95% CI (-6.50, -0.24), p = 0.0351 for ZBI.

At 6 months the estimated difference in treatment effect between severe and non-severe patients was - 1.11, 95% CI (-4.01, 1.78), p = 0.4480 for QOL-AD (patient perspective), and 0.13, 95% CI (-3.34, 3.60), p = 0.9404 for ZBI. At 12 months the estimated difference in treatment effect between severe and non- severe patients was -0.77, 95% CI (-3.61, 2.07), p = 0.5929 for QOL-AD (patient perspective), and -1.27, 95% CI (-5.27, 2.73), p = 0.5302 for ZBI.

High depressive symptoms (HADS > 11; N = 35) vs. Mild Depressive symptoms (N = 175)

At 6 months the estimated treatment effect for the non-depressed patients was 2.09, 95% CI (0.59, 3.59), p = 0.0067 for QOL-AD (patient perspective), and -1.89, 95% CI (-3.73, -0.04), p = 0.0451 for ZBI. At 12 months the estimated treatment effect for the non- depressed patients was 1.63, 95% CI (0.16, 3.11), p= 0.0299 for QOL-AD (patient perspective), and -3.50, 95% CI (-5.62, -1.39), p = 0.0013 for ZBI.

©2020 Kluger BM et al. *JAMA Neurology*.

At 6 months the estimated treatment effect for the depressed patients was 0.40, 95% CI (-3.01, 3.80), p = 0.8187 for QOL-AD (patient perspective), and -0.08, 95% CI (-4.31, 4.15), p = 0.9694 for ZBI. At 12 months the estimated treatment effect for the depressed patients was -0.29, 95% CI (-3.75, 3.17), p = 0.8693 for QOL-AD (patient perspective), and 2.24, 95% CI (-2.67, 7.16), p = 0.3685 for ZBI.

At 6 months the estimated difference in treatment effect between depressed and non-depressed patients was -1.69, 95% CI (-5.41, 2.02), p = 0.3693 for QOL-AD (patient perspective), and 1.80, 95% CI (-2.79, 6.40), p = 0.4386 for ZBI. At 12 months the estimated difference in treatment effect between depressed and non-depressed patients was -1.92, 95% CI (-5.66, 1.82), p = 0.3115 for QOL-AD (patient perspective), and 5.75, 95% CI (0.43, 11.06), p = 0.0344 for ZBI.

Health Care Use Analyses

Snapshot models modeled health care utilization as it changed across time, by counting the utilizations during the 6 weeks preceding each visit. Cumulative models added up all healthcare utilizations during the study proceeding the specified time point. Cumulative models could only include patients with complete data across all preceding times. Binary outcomes were analyzed with relative risk models, while count variables were analyzed with negative binomial models, with robust standard errors. For the snapshot models, generalized estimating equations (GEE) were used to account for repeated measures on patients.

©2020 Kluger BM et al. *JAMA Neurology*.

eTable 1. Raw Differences Between Groups in Primary and Secondary Outcomes, Summary Statistics

Outcome Measure	Time (Months)	Usual Care			Intervention		
		N	Mean	SD	N	Mean	SD
QOL-AD	0	101	34.29	5.60	104	33.89	5.69
	6	94	33.29	6.04	91	34.72	6.46
	12	84	34.37	6.38	92	34.69	6.33
QOL-AD Caregiver Perspective on Patient	0	79	34.54	6.03	76	32.94	5.82
	6	76	32.95	5.87	75	34.65	5.85
	12	74	33.62	6.78	75	34.38	6.21
ZBI	0	85	16.84	7.74	86	17.93	8.02
	6	76	15.97	7.22	75	15.04	8.06
	12	72	16.65	7.81	75	14.87	8.85
ESAS-PD	0	100	55.63	28.17	104	56.49	23.45
	6	90	54.30	23.84	93	37.62	18.07
	12	85	52.99	26.45	88	42.92	21.91
PDQ-39	0	100	34.27	16.51	105	35.19	16.82
	6	88	31.96	15.62	91	30.62	15.75
	12	86	32.05	16.08	88	30.01	15.03

©2020 Kluger BM et al. *JAMA Neurology*.

UPDRS Motor Score	0	101	37.67	17.61	104	42.81	19.44
	6	82	37.45	16.72	93	37.62	18.07
	12	79	38.59	18.64	93	39.57	18.87
MOCA Score	0	100	23.66	5.08	102	23.96	4.83
	6	77	24.40	4.81	85	24.73	5.13
	12	74	23.50	5.44	83	24.86	4.12
HADS-Depression	0	102	7.23	3.74	105	7.04	3.55
	6	87	6.77	3.73	93	6.66	4.38
	12	85	6.91	3.94	87	6.44	3.83
HADS-Anxiety	0	102	7.73	4.43	105	7.57	3.78
	6	87	6.78	4.06	93	6.32	3.70
	12	85	5.94	3.76	87	6.01	4.03
PG-12	0	99	24.35	8.61	101	25.32	9.67
	6	87	23.34	8.88	89	22.39	9.53
	12	86	22.28	9.80	87	21.97	8.16
FACIT-Sp	0	100	27.76	9.75	103	28.01	9.58
	6	89	29.05	9.04	93	28.97	9.59
	12	86	30.63	10.08	87	28.99	9.59
FACIT-Sp Meaning	0	100	11.00	3.33	103	11.21	3.08

©2020 Kluger BM et al. *JAMA Neurology*.

	6	89	11.50	3.00	93	11.40	3.50
	12	86	11.80	3.15	87	11.70	3.43
FACIT-Sp Peace	0	100	8.56	3.49	103	917	3.35
	6	89	9.42	3.41	93	9.68	3.43
	12	86	9.93	3.44	87	9.47	3.59
FACIT-Sp Faith	0	98	8.16	4.86	102	7.63	4.91
	6	87	8.10	4.92	93	7.89	4.58
	12	83	8.77	4.87	86	7.87	4.46
Patient CGIC	0	NA	NA	NA	NA	NA	NA
	6	89	-0.44	1.28	93	0.33	1.49
	12	82	-0.55	1.31	91	0.48	1.63
Caregiver HADS - Depression	0	87	4.09	2.79	86	4.50	3.34
	6	73	3.73	2.87	74	4.03	3.50
	12	72	4.31	3.25	74	4.08	3.43
Caregiver HADS - Anxiety	0	87	7.72	3.57	86	7.14	3.58
	6	73	7.08	4.12	74	5.81	3.71
	12	72	6.97	3.99	74	6.36	3.97
Caregiver FACIT-Sp	0	85	34.45	8.89	86	32.60	8.53
	6	73	33.95	8.06	76	33.81	8.80

©2020 Kluger BM et al. *JAMA Neurology*.

	12	73	33.51	7.83	74	33.41	9.42
Caregiver FACIT-Sp Meaning	0	85	13.93	2.38	86	13.59	2.65
	6	73	13.93	2.31	76	13.58	2.63
	12	73	13.58	2.60	74	13.45	2.81
Caregiver FACIT-Sp Peace	0	85	10.33	3.44	86	9.74	3.36
	6	73	10.33	3.33	76	10.66	3.26
	12	73	10.14	3.02	74	10.59	3.40
Caregiver FACIT-Sp Faith	0	83	10.06	4.96	85	9.29	4.45
	6	72	9.61	4.49	75	9.56	4.96
	12	71	9.78	4.58	73	9.40	5.13
Caregiver CGIC	0	NA	NA	NA	NA	NA	NA
	6	73	-0.74	1.28	74	0.01	1.55
	12	67	-0.79	1.29	69	0.42	1.82

©2020 Kluger BM et al. *JAMA Neurology*.

eTable 2. Differences Between Groups in Primary and Secondary Outcomes, Inferential Statistics, Unadjusted

Outcome Measure	Time (Months)	Usual Care		Intervention		Difference Between Groups	
		Estimate (95% CI)	p value	Estimate (95% CI)	p value	Estimate (95% CI)	p value
QOL-AD	6	-0.84 (-1.68, 0.01)	0.048	0.66 (-0.43, 1.75)	0.233	1.50 (0.17, 2.83)	0.027
	12	-0.43 (-1.37, 0.50)	0.361	0.68 (-0.38, 0.73)	0.207	1.11 (-0.27, 2.48)	0.114
QOL-AD Caregiver Perspectiv e on	6	-1.40 (-2.38, - 0.43)	0.005	2.09 (0.93, 3.25)	<0.001	3.49 (1.99, 5.00)	<0.001
	12	-0.76 (-1.75, 0.23)	0.132	1.81 (0.72, 2.90)	0.001	2.57 (1.11, 4.03)	<0.001
ZBI	6	-1.08 (-2.28, 0.12)	0.076	-2.28 (-3.38, - 1.18)	<0.001	-1.20 (-2.76, 0.36)	0.129
	12	0.02 (-1.32, 1.37)	0.971	-2.35 (-3.69, - 1.00)	<0.001	-2.27 (-4.11, - 0.44)	0.016
ESAS-PD	6	-0.45 (-3.86, 2.96)	0.800	-6.81 (-10.46, - 3.15)	<0.001	-6.36 (-11.03, - 1.69)	0.008
	12	-0.73 (-4.97, 3.51)	0.733	-9.66 (-13.52, - 5.80)	<0.001	-8.93 (-14.34, - 3.52)	0.001
PDQ-39	6	-1.20 (-3.57, 1.18)	0.321	-3.04 (-5.13, - 0.94)	0.005	-1.84 (-4.87, 1.19)	0.233

©2020 Kluger BM et al. *JAMA Neurology*.

	12	-0.34 (-2.66, 1.97)	0.768	-3.04 (-5.46, -0.61)	0.015	-2.69 (-5.91, -0.53)	0.101
UPDRS Motor Score	6	2.15 (0.04, 4.27)	0.046	-2.98 (-5.79, -0.18)	0.037	-5.14 (-8.63, -1.65)	0.004
	12	2.45 (-0.36, 5.26)	0.086	-1.38 (-4.78, 2.02)	0.423	-3.83 (-8.21, 0.55)	0.086
MOCA Score	6	-0.14 (-0.82, 0.55)	0.690	0.17 (-0.55, 0.90)	0.637	0.31 (-0.67, 1.30)	0.534
	12	-1.05 (-1.78, -0.32)	0.005	0.14 (-0.57, 0.85)	0.700	1.19 (0.18, 2.20)	0.021
HADS - Depression	6	-0.20 (-0.73, 0.32)	0.439	-0.34 (-0.97, 0.30)	0.296	-0.13 (-0.93, 0.67)	0.728
	12	0.121 (-0.45, 0.69)	0.656	-0.33 (-0.92, 0.25)	0.260	-0.45 (-1.26, 0.34)	0.263
HADS - Anxiety	6	-0.73 (-1.35, -0.11)	0.022	-1.19 (-1.71, -0.68)	<0.001	-0.46 (-1.23, 0.31)	0.237
	12	-1.42 (-2.04, -0.80)	<0.001	-1.30 (-1.91, -0.69)	<0.001	0.118 (-0.71, 0.95)	0.779
PG-12	6	-0.68 (-2.05, 0.68)	0.323	-2.63 (-3.91, -1.35)	<0.001	-1.95 (-3.75, -0.14)	0.035

©2020 Kluger BM et al. *JAMA Neurology*.

	12	-1.31 (-2.73, 0.11)	0.070	-2.61 (-3.92, -1.31)	<0.001	-1.30 (-3.17, 0.57)	0.171
FACIT-Sp	6	1.10 (-0.29, 2.49)	0.118	1.17 (-0.01, 2.35)	0.053	0.07 (-1.68, 1.81)	0.939
	12	2.30 (0.76, 3.83)	0.004	0.605 (-0.828, 2.04)	0.404	-1.69 (-3.73, 0.34)	0.103
FACIT-Sp Meaning	6	0.41 (-0.04, 0.87)	0.077	0.23 (-0.26, 0.71)	0.359	-0.19 (-0.83, 0.46)	0.563
	12	0.61 (0.08, 1.14)	0.024	0.42 (-0.17, 1.00)	0.161	-0.19 (-0.95, 0.56)	0.611
FACIT-Sp Peace	6	0.65 (0.07, 1.23)	0.028	0.57 (0.03, 1.11)	0.037	-0.08 (-0.83, 0.67)	0.839
	12	1.09 (0.48, 1.70)	0.001	0.17 (-0.48, 0.83)	0.598	-0.91 (-1.76, -0.07)	0.035
FACIT-Sp Faith	6	-0.00 (-0.76, 0.76)	0.995	0.36 (-0.23, 0.94)	0.229	0.36 (-0.56, 1.28)	0.441
	12	0.53 (-0.19, 1.24)	0.151	0.04 (-0.52, 0.61)	0.878	-0.48 (-1.36, 0.40)	0.283
Patient CGIC	6	-0.46 (-0.72, -0.19)	0.001	0.29 (-0.01, 0.59)	0.061	0.75 (0.35, 1.15)	<0.001
	12	-0.59 (-0.87, -0.30)	<0.001	0.41 (0.08, 0.75)	0.016	1.00 (0.57, 1.44)	<0.001

©2020 Kluger BM et al. *JAMA Neurology*.

Caregiver HADS - Depression	6	-0.20 (-0.68, 0.29)	0.424	-0.36 (-0.99, 0.28)	0.268	-0.16 (-0.94, 0.62)	0.683
	12	0.47 (-0.17, 1.12)	0.149	-0.26 (-0.85, 0.34)	0.398	-0.73 (-1.58, 0.13)	0.095
Caregiver HADS - Anxiety	6	-0.52 (-1.21, 0.16)	0.133	-1.21 (-1.90, -0.52)	0.001	-0.69 (-1.63, 0.26)	0.153
	12	-0.40 (-1.13, 0.34)	0.285	-0.68 (-1.37, 0.02)	0.057	-0.28 (-1.27, 0.71)	0.576
Caregiver FACIT-Sp	6	-0.27 (-1.42, 0.89)	0.650	0.68 (-0.57, 1.94)	0.283	0.95 (-0.69, 2.59)	0.256
	12	-0.90 (-2.12, 0.31)	0.144	0.42 (-0.81, 1.66)	0.495	1.33 (-0.35, 3.01)	0.121
Caregiver FACIT-Sp Meaning	6	-0.05 (-0.466, 0.375)	0.831	0.03 (-0.37, 0.42)	0.897	0.07 (-0.48, 0.62)	0.801
	12	-0.41 (-0.87, 0.05)	0.083	-0.09 (-0.54, 0.36)	0.694	0.32 (-0.31, 0.95)	0.315
Caregiver FACIT-Sp Peace	6	0.11 (-0.56, 0.78)	0.753	0.75 (0.15, 1.34)	0.014	0.64 (-0.20, 1.48)	0.133
	12	-0.14 (-0.71, 0.43)	0.627	0.67 (0.08, 1.27)	0.027	0.81 (0.04, 1.59)	0.040
Caregiver FACIT-Sp Faith	6	-0.24 (-0.78, 0.31)	0.395	-0.09 (-0.74, 0.56)	0.784	0.15 (-0.68, 0.97)	0.730

©2020 Kluger BM et al. *JAMA Neurology*.

	12	-0.26 (-0.95, 0.42)	0.443	-0.21 (-0.75, 0.33)	0.434	0.05 (-0.81, 0.91)	0.914
Caregiver CGIC	6	-0.75 (-1.04, -0.46)	<0.001	-0.05 (-0.41, 0.30)	0.764	0.70 (0.24, 1.15)	0.003
	12	-0.81 (-1.11, -0.50)	<0.001	0.36 (-0.07, 0.79)	0.086	1.17 (0.65, 1.69)	<0.001

©2020 Kluger BM et al. *JAMA Neurology*.

eTable 3. Snapshot Models for Patients for the 6-Week Periods Proceeding Baseline, 6 Months, and 12 Months

A. Have you gone to an emergency room about your own health (yes or no)?

	Sample (Proportion)		Model (Proportion Estimate (95% CI))	
	Usual	Palliative	Usual	Palliative
Baseline	13/103 (0.1262)	17/106 (0.1604)	0.1435 (0.1031, 0.1999)	
6 Months	8/92 (0.0870)	13/93 (0.1398)	0.0888 (0.0466, 0.1695)	0.1386 (0.0835, 0.2302)
12 Months	15/87 (0.1724)	16/94 (0.1702)	0.1747 (0.1114, 0.2739)	0.1674 (0.1065, 0.2631)

©2020 Kluger BM et al. *JAMA Neurology*.

Comparison to Baseline (Model) (Ratio (95% CI), p value)		Palliative vs Usual, Comparison to Baseline (Model) (Ratio-Ratio (95% CI), p value)
Usual	Palliative	
0.6190 (0.3063, 1.2511), 0.1401	0.9659 (0.5357, 1.7415), 0.9073	1.5604 (0.6876, 3.5413), 0.2846
1.2168 (0.7211, 2.0533), 0.4853	1.1661 (0.6783, 2.0049), 0.5869	0.9583 (0.5079, 1.8081), 0.8956

B. How many times have you gone to an emergency room about your own health?

	Sample (N, Mean (SD))		Model (Mean Estimate (95% CI))	
	Usual	Palliative	Usual	Palliative
Baseline	103, 0.1845 (0.6824)	105, 0.1619 (0.3953)	0.1733 (0.1122, 0.2675)	
6 Months	92, 0.0978 (0.3335)	93, 0.1828 (0.5096)	0.0982 (0.0493, 0.1958)	0.1826 (0.1039, 0.3210)
12 Months	87, 0.1954 (0.4534)	94, 0.2128 (0.5053)	0.1937 (0.1187, 0.3159)	0.2133 (0.1326, 0.3432)

©2020 Kluger BM et al. *JAMA Neurology*.

Comparison to Baseline (Model) (Ratio (95% CI), p value)		Palliative vs Usual, Comparison to Baseline (Model) (Ratio-Ratio (95% CI), p value)
Usual	Palliative	
0.5670 (0.2517, 1.2775), 0.1497	1.0538 (0.5162, 2.1513), 0.8667	1.8585 (0.7623, 4.5310), 0.1849
1.1177 (0.5786, 2.1592), 0.7415	1.2312 (0.6622, 2.2892), 0.5217	1.1015 (0.5569, 2.1787), 0.7809

C. Have you been a patient in a hospital overnight (yes or no)?

	Sample (Proportion)		Model (Proportion Estimate (95% CI))	
	Usual	Palliative	Usual	Palliative
Baseline	6/103 (0.0583)	4/106 (0.0377)	0.0478 (0.0261, 0.0876)	
6 Months	3/92 (0.0326)	4/93 (0.0430)	0.0315 (0.0099, 0.1002)	0.0444 (0.0176, 0.1115)
12 Months	3/87 (0.0345)	7/94 (0.0745)	0.0323 (0.0100, 0.1047)	0.0765 (0.0381, 0.1536)

Comparison to Baseline (Model) (Ratio (95% CI), p value)		Palliative vs Usual, Comparison to Baseline (Model) (Ratio-Ratio (95% CI), p value)
Usual	Palliative	
0.6584 (0.1733, 2.5007), 0.4966	0.9273 (0.3356, 2.5620), 0.8830	1.4084 (0.3214, 6.1710), 0.6385
0.6749 (0.1930, 2.3597), 0.4951	1.5988 (0.6336, 4.0348), 0.3697	2.3690 (0.6053, 9.2724), 0.1989

D. How many times have you been a patient in a hospital overnight?

	Sample (N, Mean (SD))		Model (Mean Estimate (95% CI))	
	Usual	Palliative	Usual	Palliative
Baseline	103, 0.0874 (0.4452)	106, 0.0378 (0.1915)	0.0622 (0.0296, 0.1306)	
6 Months	92, 0.0326 (0.1786)	93, 0.0430 (0.2040)	0.0317 (0.0100, 0.0999)	0.0441 (0.0173, 0.1120)
12 Months	87, 0.0460 (0.2601)	94, 0.0745 (0.2639)	0.0444 (0.0131, 0.1507)	0.0757 (0.0375, 0.1527)

Comparison to Baseline (Model) (Ratio (95% CI), p value)		Palliative vs Usual, Comparison to Baseline (Model) (Ratio-Ratio (95% CI), p value)
Usual	Palliative	
0.5090 (0.1245, 2.0806), 0.3224	0.7085 (0.2310, 2.1728), 0.5406	1.3919 (0.3170, 6.1110), 0.6504
0.7144 (0.1740, 2.9329), 0.6227	1.2170 (0.4399, 3.3666), 0.7123	1.7035 (0.4165, 6.9679), 0.4349

E. Did any medically trained person come to patient's home to help [him/her] (yes or no)?

	Sample (Proportion)		Model (Proportion Estimate (95% CI))	
	Usual	Palliative	Usual	Palliative
Baseline	12/103 (0.1165)	17/106 (0.1604)	0.1388 (0.0990, 0.1945)	
6 Months	13/92 (0.1413)	15/93 (0.1613)	0.1506 (0.0951, 0.2386)	0.1666 (0.1090, 0.2547)
12 Months	19/87 (0.2184)	14/94 (0.1489)	0.2356 (0.1639, 0.3388)	0.1577 (0.1003, 0.2478)

©2020 Kluger BM et al. *JAMA Neurology*.

Comparison to Baseline (Model) (Ratio (95% CI), p value)		Palliative vs Usual, Comparison to Baseline (Model) (Ratio-Ratio (95% CI), p value)
Usual	Palliative	
1.0854 (0.6581, 1.7901), 0.7587	1.2007 (0.7633, 1.8887), 0.4527	1.1062 (0.6061, 2.0188), 0.7465
1.6982 (1.0873, 2.6525), 0.0428	1.1363 (0.6793, 1.9007), 0.6395	0.6691 (0.3782, 1.1837), 0.1738

F. How many times did a medically trained person come to patient's home to help [him/her]?

	Sample (N, Mean (SD))		Model (Mean Estimate (95% CI))	
	Usual	Palliative	Usual	Palliative
Baseline	103, 1.2233 (6.1164)	106, 1.3491 (5.1511)	1.2871 (0.7120, 2.3266)	
6 Months	92, 1.0000 (4.6523)	93, 1.5376 (5.5454)	0.9651 (0.4285, 2.1733)	1.5766 (0.7458, 3.3325)
12 Months	87, 1.9885 (6.3694)	93, 1.3118 (5.8773)	2.0508 (1.0726, 3.9211)	1.2444 (0.4745, 3.2636)

©2020 Kluger BM et al. *JAMA Neurology*.

Comparison to Baseline (Model) (Ratio (95% CI), p value)		Palliative vs Usual, Comparison to Baseline (Model) (Ratio-Ratio (95% CI), p value)
Usual	Palliative	
0.7498 (0.3674, 1.5304), 0.4002	1.2249 (0.5190, 2.8912), 0.6562	1.6336 (0.5822, 4.5841), 0.3617
1.5933 (0.6588, 3.8537), 0.3462	0.9668 (0.3047, 3.0673), 0.9493	0.6068 (0.1898, 1.9398), 0.3746

G. Have you seen or talked to a social worker, therapist, psychologist, or religious/spiritual councilor (outside of the study team; yes or no)?

	Sample (Proportion)		Model (Proportion Estimate (95% CI))	
	Usual	Palliative	Usual	Palliative
Baseline	25/96 (0.2604)	26/100 (0.2600)	0.2595 (0.2050, 0.3285)	
6 Months	23/90 (0.2556)	15/93 (0.1613)	0.2543 (0.1801, 0.3591)	0.1632 (0.1033, 0.2580)

©2020 Kluger BM et al. *JAMA Neurology*.

12 Months	22/82 (0.2683)	20/93 (0.2151)	0.2622 (0.1863, 0.3689)	0.2270 (0.1578, 0.3267)

Comparison to Baseline (Model) (Ratio (95% CI), p value)		Palliative vs Usual, Comparison to Baseline (Model) (Ratio-Ratio (95% CI), p value)
Usual	Palliative	
0.9802 (0.6741, 1.4254), 0.9161	0.6290 (0.3799, 1.0416), 0.0500	0.6418 (0.3635, 1.1330), 0.1228
1.0104 (0.7115, 1.4349), 0.9534	0.8749 (0.5862, 1.3056), 0.5084	0.8658 (0.5330, 1.4065), 0.5649

H. How many times have you seen or talked to a social worker, therapist, psychologist, or religious/spiritual counselor (outside of the study team)?

	Sample (N, Mean (SD))		Model (Mean Estimate (95% CI))	
	Usual	Palliative	Usual	Palliative
Baseline	96, 0.8542 (2.3663)	99, 1.0707 (3.3994)	0.9387 (0.6119, 1.4399)	

©2020 Kluger BM et al. *JAMA Neurology*.

6 Months	90, 0.7556 (2.2451)	93, 0.5269 (2.0726)	0.8087 (0.4733, 1.3818)	0.5076 (0.2346, 1.0985)
12 Months	82, 0.6098 (1.4295)	93, 0.5054 (1.3563)	0.6044 (0.3689, 0.9901)	0.5187 (0.3035, 0.8863)

Comparison to Baseline (Model) (Ratio (95% CI), p value)		Palliative vs Usual, Comparison to Baseline (Model) (Ratio-Ratio (95% CI), p value)
Usual	Palliative	
0.8615 (0.5270, 1.4083), 0.5517	0.5408 (0.2406, 1.2153), 0.1088	0.6277 (0.2557, 1.5410), 0.2869
0.6438 (0.3603, 1.1505), 0.1468	0.5526 (0.2713, 1.1254), 0.1195	0.8582 (0.4148, 1.7759), 0.5653

©2020 Kluger BM et al. *JAMA Neurology*.

eTable 4. Cumulative Models for Patients for 6 Months and 12 Months, Patients With Complete Data Only

A. How many times have you gone to an emergency room about your own health?

	Sample (N, Mean (SD))		Model (Mean Estimate (95% CI))		Palliative vs Usual (Model) (Ratio (95% CI), p value)
	Usual	Palliative	Usual	Palliative	
6 Months	81, 0.3333 (0.7746)	81, 0.5679 (1.1173)	0.3333 (0.2016, 0.5512)	0.5679 (0.3710, 0.8694)	1.7037 (0.8815, 3.2930), 0.1209
12 Months	71, 0.8873 (1.5358)	65, 0.9385 (1.3906)	0.8873 (0.5949, 1.3234)	0.9385 (0.6564, 1.3417)	1.0576 (0.6187, 1.8081), 0.8374

B. How many times have you been a patient in a hospital overnight?

	Sample (N, Mean (SD))		Model (Mean Estimate (95% CI))		Palliative vs Usual (Model) (Ratio (95% CI), p value)
	Usual	Palliative	Usual	Palliative	
6 Months	81, 0.1605 (0.4862)	81, 0.1728 (0.7034)	0.1605 (0.0833, 0.3092)	0.1728 (0.0716, 0.4170)	1.0769 (0.3592, 3.2289), 0.8960

©2020 Kluger BM et al. *JAMA Neurology*.

12 Months	71, 0.2817 (0.7208)	65, 0.4308 (1.4892)	0.2817 (0.1560, 0.5087)	0.4308 (0.1871, 0.9918)	1.5292 (0.5503, 4.2498), 0.4614

C. How many times did a medically trained person come to patient's home to help [him/her]?

	Sample (N, Mean (SD))		Model (Mean Estimate (95% CI))		Palliative vs Usual (Model) (Ratio (95% CI), p value)
	Usual	Palliative	Usual	Palliative	
6 Months	80, 4.1375 (10.7806)	80, 4.6125 (12.9961)	4.1375 (2.3460, 7.2971)	4.6125 (2.4973, 8.5192)	1.1148 (0.4834, 2.5712), 0.8002
12 Months	70, 12.4143 (30.4429)	64, 6.7656 (19.5949)	12.4143 (7.0182, 21.9594)	6.7656 (3.3463, 13.6789)	0.5450 (0.2202, 1.3486), 0.1973

D. How many times have you seen or talked to a social worker, therapist, psychologist, or religious/spiritual councilor (outside of the study team)?

	Sample (N, Mean (SD))		Model (Mean Estimate (95% CI))		Palliative vs Usual (Model) (Ratio (95% CI), p value)
	Usual	Palliative	Usual	Palliative	

©2020 Kluger BM et al. *JAMA Neurology*.

6 Months	78, 2.0000 (5.6866)	81, 1.5309 (3.1507)	2.0000 (1.0685, 3.7437)	1.5309 (0.9806, 2.3899)	0.7654 (0.3547, 1.6516), 0.5200
12 Months	66, 4.2273 (12.1653)	63, 2.5397 (5.8443)	4.2273 (2.1224, 8.4196)	2.5397 (1.4453, 4.4627)	0.6008 (0.2467, 1.4633), 0.3101

©2020 Kluger BM et al. *JAMA Neurology*.

eReferences

1. Kluger BM, Katz M, Galifianakis N, et al. Does outpatient palliative care improve patient-centered outcomes in Parkinson's disease: Rationale, design, and implementation of a pragmatic comparative effectiveness trial. *Contemp Clin Trials.* 2019;79:28-36.
2. Kluger BM, Persenaire MJ, Holden SK, et al. Implementation issues relevant to outpatient neurology palliative care. *Ann Palliat Med.* 2018;7(3):339-348.
3. Clark D. 'Total pain', disciplinary power and the body in the work of Cicely Saunders, 1958-1967. *Soc Sci Med.* 1999;49(6):727-736.

©2020 Kluger BM et al. *JAMA Neurology*.
