# Supplementary material for: Comparison of Integrated Outpatient Palliative Care With Standard Care in Patients With Parkinson Disease and Related Disorders: A Randomized Clinical Trial
Source: JAMA Neurol. 2020 Feb 10;77(5):1–11. doi: 10.1001/jamaneurol.2019.4992 (PMC7042842; doi:10.1001/jamaneurol.2019.4992)
Supplement: Supplement 3. — Data Sharing Statement [file jamaneurol-77-551-s003.pdf]

Data Sharing Statement

Kluger. Comparison of Integrated Outpatient Palliative Care With Standard Care in Patients With Parkinson Disease and Related Disorders. *JAMA Neurol*. Published February 10, 2020. 10.1001/jamaneurol.2019.4992

Data
Data available: Yes
Data types: Deidentified participant data, Data dictionary
How to access data: https://palliativecareresearch.org/corescenters/data-informatics-statistics-core-disc/pcrc-de-identified-data-repository-didr
When available: With publication

Supporting Documents
Document types: Statistical/analytic code
How to access documents: https://palliativecareresearch.org/corescenters/data-informatics-statistics-core-disc/pcrc-de-identified-data-repository-didr
When available: With publication

Additional Information
Who can access the data: researchers whose proposed use of the data has been approved
Types of analyses: for any purpose
Mechanisms of data availability: after approval or proposal
